# Supplementary material for: Consumer risk perception towards pesticide-stained tomatoes in Uganda
Source: PLoS One. 2023 Dec 15;18(12):e0247740. doi: 10.1371/journal.pone.0247740 (PMC10723735; doi:10.1371/journal.pone.0247740)
Supplement: S1 File — (PDF) [file pone.0247740.s001.pdf]

S1 File: Other Demographic characteristics of consumers n=425

Variable	Category	Frequency (%)
Marital status	Single	51 (10.90)
	Married	368 (78.63)
	Separated	30 (6.41)
	widowed	19 (4.06)
Occupation	Housewife	42 (8.99)
	Teacher	09 (1.93)
n=467	Farmer	238 (50.96)
	Gov't	04 (0.86)
	NGO	04 (0.86)
	Self-employed	164 (35.12)
	Other	06 (1.28)
