# Supplementary material for: Consumer risk perception towards pesticide-stained tomatoes in Uganda
Source: PLoS One. 2023 Dec 15;18(12):e0247740. doi: 10.1371/journal.pone.0247740 (PMC10723735; doi:10.1371/journal.pone.0247740)
Supplement: S2 File — (PDF) [file pone.0247740.s002.pdf]

S2 File: Consumer attitudes towards pesticide stained tomatoes for optimism, trust and pessimism

Statements	Frequency (%)					
	Total (n)	Strongly agree	Agree	Not sure	Disagree	Strongly disagree
<u>Optimism</u> I am optimistic about the safety of tomatoes stained with pesticides	468	112 (23.9)	76 (16.2)	21 (4.5)	189 (40.4)	70(15.0)
I am confident that pesticides stained tomatoes sold on market and farm are safe	468	70 (15.0)	64 (13.7)	21 (4.5)	237 (50.6)	76(16.2)
I am satisfied with the safety of tomatoes sold with pesticide residues	467	86 (18.4)	58 (12.4)	38 Text (8.1)	199 (42.6)	86(18.4)
Average (%)	468	89.3(19.1)	66(14.1)	26.7(5.7)	208.3(44.5)	77.3(16.5)
<u>Resized Computed percentages for optimism (3 likert scale)</u>			**33.21**	**5.71**	**61.08**	
<u>Pessimism</u> I worry about the safety of food	468	221(47.2)	146 (31.2)	14 (3.0)	69 (14.7)	18 (3.9)
I feel uncomfortable about the safety of food.	466	189(40.6)	154 (33.1)	11 (2.4)	94 (20.2)	18 (3.9)
I am suspicious about some pesticide sprayed foods like tomatoes	468	201(43.0)	129 (27.6)	8 (1.7)	78 (16.7)	52 (11.1)
Average (%)	467	204(43.6)	143(30.6)	11(2.4)	80.3(17.2)	29.3(6.3)
<u>Resized Computed percentages for pessimism (3 Likert scale)</u>			**74.30**	**2.36**	**23.46**	
<u>Trust</u> Vendors have competence to control the safety of tomatoes.	467	41(8.8)	63 (13.5)	46 (9.9)	219 (46.9)	98 (21.0)
Vendors have enough knowledge to guarantee the safety of tomatoes	466	36 (7.7)	53 (11.4)	47(10.1)	219(47.0)	111 (23.8)
Vendors are honest about the safety of tomatoes sold.	464	17 (3.7)	20 (4.3)	19 (4.1)	251(54.1)	157 (33.9)
Vendors give special attention about the safety of tomatoes.	462	27 (5.8)	34 (7.4)	46 (10.0)	263(56.9)	92 (20.0)
Vendors are sufficiently open about the general safety of tomatoes.	466	17 (3.7)	19 (4.1)	30 (6.44)	23 (50.0)	167 (35.8)
Average (%)	466	27.6(5.9)	37.8(8.1)	37.6(8.1)	237(51.0)	125(26.9)
<u>Resized percentages for trust (3 Likert scale)</u>			**14.05**	**8.09**	**77.7**	
