# Supplementary material for: Consumer risk perception towards pesticide-stained tomatoes in Uganda
Source: PLoS One. 2023 Dec 15;18(12):e0247740. doi: 10.1371/journal.pone.0247740 (PMC10723735; doi:10.1371/journal.pone.0247740)
Supplement: S3 File — (PDF) [file pone.0247740.s003.pdf]

S3 File: Fisher-exact tests for the factors associated with Consumer risk perception

Marital status	Consumer risk perception freq (%)		Fishers-exact p-values
	**High risk**	**Low risk**	0.265
Single	1/48 (2.1)	47/48(97.9)	
Married	14/306(45.8)	292/306(54.2)	
Separated	3/28(7.1)	25/28(92.9)	
Widowed	1 /14(7.1)	13/14(92.9)	
Age groups			0.680
≤30	7/130(5.4)	123/130(94.6)	
31-40	8/129(6.2)	121/129(93.8)	
41-50	3/75(4.0)	72/75(96.0)	
51-60	0/39(0.0)	39/39(100.0)	
61-70	1/17(5.9)	16/17(94.1)	
>70	0/6(0.0)	6/6(100.0)	
Residence			0.462
Rural	9/145(6.2)	136/145(93.8)	
Urban	7/139(5.0)	132/139(95.0)	
Peri-urban	3/112(2.7)	109/112(96.3)	
Gender			0.581
Female	10/207(4.8)	197/207(95.2)	
Male	9/189(4.8)	180/189(95.2)	
Education level			0.975
None	1/29(3.4)	28/29(96.6)	
primary	11/227(48.5)	216/227(51.5)	
secondary	6/109(55.0)	103/109(45.0)	
tertiary	1/31(3.2)	30/31(96.8)	
