# Supplementary material for: Consumer risk perception towards pesticide-stained tomatoes in Uganda
Source: PLoS One. 2023 Dec 15;18(12):e0247740. doi: 10.1371/journal.pone.0247740 (PMC10723735; doi:10.1371/journal.pone.0247740)
Supplement: S4 File — (PDF) [file pone.0247740.s004.pdf]

S4 File: Simple logistic regression of consumer risk perception vs awareness about pesticide residues

	Consumer Risk perception Freq (%)		Odds Ratio	Std. Err.	P>|z|	[95% CI]	
Aware of residues in tomatoes	High risk perception	Low risk perception					
No	6/19(31.6)	4/377(1.1)	1.0				
Yes	13/19(68.4)	371/377(98.4)	42.80769	30.15695	0.000	10.76147	170.2833
Not sure	0/19(0.0)	2/377(0.5)	1	-	-		
