# Supplementary material for: Consumer risk perception towards pesticide-stained tomatoes in Uganda
Source: PLoS One. 2023 Dec 15;18(12):e0247740. doi: 10.1371/journal.pone.0247740 (PMC10723735; doi:10.1371/journal.pone.0247740)
Supplement: S5 File — (PDF) [file pone.0247740.s005.pdf]

S5 File: Questionnaire English Version: Consumers risk perception towards pesticide stained tomatoes in Uganda.

A. General information

Name of Interviewer: ……………………………………………… Questionnaire number: …………

District………………………………...Sub-county……………………..village………………………….

Date of interview: ……../……../ 2019

Write the right code in the code column.

SN	Questions	Responses	Code	Var name
**A.**	**Background characteristics of Respondents**			
1	Respondent Gender	1= Male<br>2= Female		Gender
2	How old are you in completed years?	………………….. years	Na	Age
3	What is your current marital status?	1= Single<br>2= Married<br>3= Separated or divorced<br>4= Widowed		Maristatus
4	Residence	1=rural<br>2=urban<br>3=peri-urban		residence
5	What is your level of education?	1= No formal education<br>2= P1-P4<br>3= P5-P7<br>4= Secondary (O'level)<br>5= Secondary (A'level)<br>6= Tertiary-University<br>7= Tertiary- non-university		Educlevel
6	What is your occupation?	1= Housewife		Occup

		2= Teacher<br>3=Farmer<br>4= Government worker<br>5=NGO employee<br>6=Self employed<br>7=Other<br>specify………………………		
7	What is your monthly salary estimation (in UGX)?	1=<150,000<br>2=150,000-300,000<br>3=310,000-550,000<br>5=560,000-750,000<br>6=760,000-950,000<br>7=>1,000,000<br>8=None		Monthlysalary
8	How many family members do you cater for?			famstructure
9	How many are below 5 years of age?			
10	How many are above 70 years of age?			
11	What active role do you play in food preparation for your household?	1=Buying food from the vendors<br>2=Meeting food expenses<br>3=Preparation; cooking and Serving food<br>4=Others-<br>…………………………..		
**B. Knowledge on health and safety (health literacy):**<br>***Interviewer: I would like us to talk about pesticides and health***				
12	What are pesticides?	………………………………<br>………………………………<br>………………………………<br>………………		Pesticides definition
13	Are pesticides harmful to human health?	1= yes<br>2=no		

		3=I don't know		
14	If Yes, what are some of the negative effects of pesticides?	1=Death<br>2=Chronic diseases like cancers<br>3=Skin rash<br>4=Headaches<br>5=Stomach aches<br>6=Vomiting<br>7= Don't know		Effects
15	Have you ever had any information about tomato safety and pesticides handling?	1= Yes<br>2=No ***If no, skip to 18***		
16	If yes, from which source?	1= Media(radio,TV,)<br>2=Medical professions doctors and health educators etc<br>3= NGO<br>4=Government<br>5=Friends and family members<br>6=others specify<br>………………………………<br>………..……………………<br>…………		Information source
17	Do you think that tomatoes sold at the vendor stall contain pesticides residues?	1=Yes<br>2=No<br>3=Not sure		
18	If yes, do you buy them?	1=Yes<br>2=No		
19	If yes, Why do you buy them?	1=No perceived effect of residues<br>2=Prepare them before cooking<br>3=Have no choice, (**have to**		

		**eat)**<br>Other<br>specify……………………..		
20	How do you prepare tomatoes before cooking them?	1=Wash them with cold water<br>2=Wash them with warm water<br>3=Peel them<br>4=None<br>5=Others<br>specify………………..		
21	Which is better washing and peeling tomatoes?	1=Washing them with cold water<br>2=Washing them with warm water<br>3=Peeling them<br>4=Don't know		
22	Where do you buy your tomatoes?	1=Local stall<br>2=General retailer market<br>3=Supermarket<br>4=Farm<br>5=General wholesaler market		
23	Do you think cooking tomatoes has an effect on the pesticides residues in them?	1=Yes<br>2=No<br>3=Don't know		
**C. risk perception of consumer on pesticides residues on tomatoes sold in markets, supermarkets and local stalls in Uganda**				
**To what extend do you agree or disagree with the following statements as risk to safety of tomatoes stained with pesticides and influencers of your choice for decision making at the vendor/market place.**				
	Optimism			
24	I am optimistic about the safety of tomatoes stained with pesticides	1= Strongly agree<br>2= Agree		

		3= Neither agree nor disagree<br>4= Disagree<br>5= Strongly disagree		
25	I am confident that pesticides stained tomatoes sold on market and farm are safe	1= Strongly agree<br>2= Agree<br>3= Neither agree nor disagree<br>4= Disagree<br>5= Strongly disagree		
26	I am satisfied with the safety of tomatoes sold with pesticide residues	1= Strongly agree<br>2= Agree<br>3= Neither agree nor disagree<br>4= Disagree<br>5= Strongly disagree		
	Pessimism			
27	I worry about the safety of food	1= strongly agree<br>2= Agree<br>3= Neither agree nor disagree<br>4= disagree<br>5= strongly disagree		
28	I feel uncomfortable about the safety of food.	1= strongly agree<br>2= Agree<br>3= Neither agree nor disagree<br>4= Disagree<br>5= Strongly disagree		
29	from previous food safety incidents, I am suspicious about some sprayed foods like tomatoes	1= Strongly agree<br>2= Agree<br>3= Neither agree nor disagree<br>4= Disagree<br>5= Strongly disagree		
	Trust			
30	Vendors have competence to control the	1= Strongly agree<br>2= Agree		

	safety of tomatoes.	3= Neither agree nor disagree<br>4= Disagree<br>5= Strongly disagree		
31	Vendors have enough knowledge to guarantee the safety of tomatoes	1= Strongly agree<br>2= Agree<br>3= Neither agree nor disagree<br>4= Disagree<br>5= Strongly disagree		
32	Vendors are honest about the safety of tomatoes sold.	1= Strongly agree<br>2= Agree<br>3= Neither agree nor disagree<br>4= Disagree<br>5= Strongly disagree		
33	Vendors give special attention about the safety of tomatoes.	1= Strongly agree<br>2= Agree<br>3= Neither agree nor disagree<br>4= Disagree<br>5= Strongly disagree		
34	Vendors are sufficiently open about the general safety of tomatoes.	1= Strongly agree<br>2= Agree<br>3= Neither agree nor disagree<br>4= Disagree<br>5= Strongly disagree		
	Recall of any food safety incident (yes or No response)			
35	Do you recall any food safety incidence in your areas which caused adverse event? if yes, what incident do you recall?	………………………………<br>………………………………<br>………………………………<br>………………………………<br>………………………………<br>………………………………<br>………………………………		

		............***if no, skip to 37.***		
	General Safety of tomatoes on Ugandan markets			
36	How much confidence do you generally have on the safety of tomatoes on Ugandan market (very strong confidence/ very weak confidence)	1= Very strongly confident<br>2= Confident<br>3= Not sure<br>4= Weak confident<br>5= Very weak confidence		
	Other questions on practices			
37	Before buying, I consider the freshness of the tomatoes much more than the stains on them.	1= Strongly agree<br>2= Agree<br>3= Neither agree nor disagree<br>4= Disagree<br>5= Strongly disagree		
38	Before buying, I consider the color/ripeness of the tomatoes rather than the stains on them.	1= Strongly agree<br>2= Agree<br>3= Neither agree nor disagree<br>4= Disagree<br>5= Strongly disagree		
39	Before buying, I consider the sizes of the tomatoes rather than the stains on them.	1= Strongly agree<br>2= Agree<br>3= Neither agree nor disagree<br>4= Disagree<br>5= Strongly disagree		
40	Before buying, I consider the price of the tomatoes than the stains on them.	1= Strongly agree<br>2= Agree<br>3= Neither agree nor disagree<br>4= Disagree<br>5= Strongly disagree		

Thank you very much for your time!
