# Supplementary material for: Consumer risk perception towards pesticide-stained tomatoes in Uganda
Source: PLoS One. 2023 Dec 15;18(12):e0247740. doi: 10.1371/journal.pone.0247740 (PMC10723735; doi:10.1371/journal.pone.0247740)
Supplement: S6 File — (PDF) [file pone.0247740.s006.pdf]

S6 File: Focus Group Discussion guide for Consumers risk perception towards Pesticides stained tomatoes in Uganda

FOCUS GROUP DISCUSSION GUIDE

Fill in the required information accordingly

Name of Interviewee: ……………………………………………………………….

Contact number: ………………………………………………………………….

District…………………...Sub-county………………………...village……………...

Date of interview: ……./……../ 2019

Demographic information

1. Age…………………………….
2. Gender…………………………
3. Occupation…………………….
4. For how long have you been involved in this occupation?
    a. <1 year
    b. 1-2 years
    c. 2-5 years
    d. 5-10 years
    e. More than 10 years

Thank you for filling the questionnaire

Welcome remarks, introduction and instructions to participants

Welcome and thank you for volunteering to take part in this focus group discussion. You have been asked to participate as your point of view is important. I realize you are busy and I appreciate your time.

Introduction: This focus group discussion is designed to assess your knowledge, risk perception and what factors you think drive consumers' risk perception about pesticide stains on tomatoes. This discussion will last for an hour and a half. The discussion will be recorded on audio tape to allow its remembrance.

Anonymity: Despite being taped; I would like to assure you that the discussion will be anonymous. The tapes will be kept safely in a locked facility until they are transcribed word for word, then they will be destroyed. The transcribed notes of the focus group will contain no information that would allow individual subjects to be linked to specific statements. You should try to answer and comment as accurately and truthfully as possible. I and the other focus group participants would appreciate it if you would refrain from discussing the

comments of other group members outside the focus group. If there are any questions or discussions that you do not wish to answer or participate in, you do not have to do so; however please try to answer and be as involved as possible.

Contact information:

If you have any questions regarding this study, call the Principal Investigator; Mr. Sekabojja Daniel on 0784231200 OR Mr. Ssekkadde Peter on 070781443280.

If you have any issues pertaining to your rights and participation in the study please contact Dr. Suzan Kiwanuka, the Chairperson of the Institutional Review Board, Makerere University School of Public Health on telephone number 0772886377. Or the Uganda National Council of Science and Technology, on plot 6 Kimera Road Ntinda, Kampala or call on Tel 0414 705 500

Ground rules

- One person should speak at a time. Respect each other during the discussion
- There are no right or wrong answers
- You do not have to speak in any particular order
- If you have something to say, do not hesitate. All your views are important for this study
- You have your own opinion and you do not have to agree with the views of other participants

  Are there any questions?

Now we can start

Warm up

- It is important that we introduce ourselves (Name and occupation)

Introductory question

Have a few minutes and reflect on pesticide stains on tomatoes. Have you had encounters with tomatoes stained with pesticides? Can any of you share how you felt the first time this occurred?

Guiding questions

- Are you aware of pesticide stains on tomatoes?
- Do you think they (stains) have implications on the consumer health? (if, yes how, and if no, why?)
- How do you perceive these stains? (Likert scale)
- What would do you consider when buying tomatoes?
- Do farmers spray tomatoes shortly (a few days) before harvesting? Why think so?
- Do vendors spray tomatoes when they are on the stall? Why do you think so?

Concluding question

- In all discussed today what do you think is the most important issues raised concerning pesticide residues/stains on tomatoes?

Conclusion

- Your views are valuable to the study
- Hopefully, you found the discussion worth your time
- If you have any complaint, please feel free to contact the PI for this study
- As a reminder, your details (identity) will remain confidential

Thank you for your active participation.
