# Supplementary material for: Consumer risk perception towards pesticide-stained tomatoes in Uganda
Source: PLoS One. 2023 Dec 15;18(12):e0247740. doi: 10.1371/journal.pone.0247740 (PMC10723735; doi:10.1371/journal.pone.0247740)

S7 File: A model for estimating the relationship between consumer perceptions, attitudes and behavioral intentions

FIGURE 1
A Conceptual Framework Depicting the Relationships Among Consumer Perceptions, Attitudes, and Behavioral Intentions

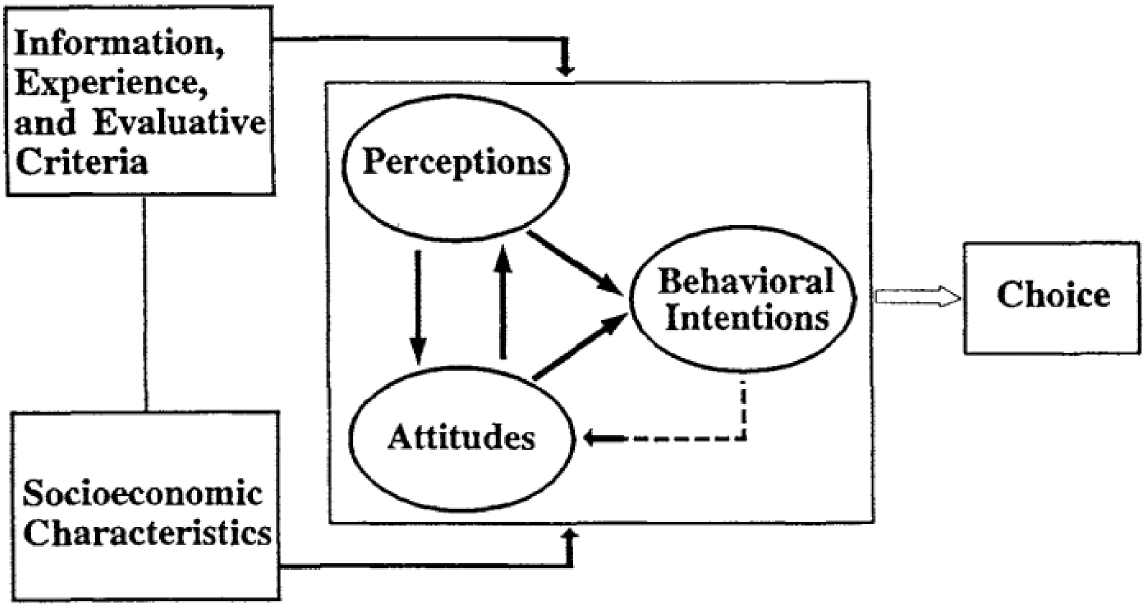

Supplement: S7 File — (PDF) [file pone.0247740.s007.pdf]
