# Supplementary material for: Consumer risk perception towards pesticide-stained tomatoes in Uganda
Source: PLoS One. 2023 Dec 15;18(12):e0247740. doi: 10.1371/journal.pone.0247740 (PMC10723735; doi:10.1371/journal.pone.0247740)
Supplement: Attachment — Submitted filename: PONE-D-21-04826_R1_reviewer_EM.pdf [file pone.0247740.s015.pdf]

PLOS ONE

Consumer risk perception towards pesticide stained tomatoes in Uganda.

--Manuscript Draft--

**Manuscript Number:**	PONE-D-21-04826R1
**Article Type:**	Research Article
**Full Title:**	Consumer risk perception towards pesticide stained tomatoes in Uganda.
**Short Title:**	Risk perception towards pesticide stained tomatoes
**Corresponding Author:**	Daniel Sekabojja<br>Uganda National Association of Community and Occupational Health<br>Kampala, UGANDA
**Keywords:**	Consumer Risk Perception; Food safety; Pesticide residues
**Abstract:**	Background: Tomatoes are consumed daily. Unfortunately, abuse of pesticides application by vegetable growers in Uganda increases risks to pesticide residues exposure among consumers, as they may be above European Union Maximum Residue Limits (used as a standard in Uganda). This study was aimed to determine consumer attitudes and risk perception towards pesticide stained tomatoes in Uganda.<br><br>Methods: A mixed methods cross-sectional study sampled 468 household consumers in four regions of Uganda, in each region selecting one district (project interventional area). In each district, 60 household members from three randomly selected Sub Counties were interviewed in addition to 9 tomato handlers (3 tomato farmers, 3 tomato retailers and 3 tomato wholesalers) who participated in Focus Group Discussion (FGDs) per district. Collected data was entered into MS-Excel 13 and exported into STATA SE 14.0 for cleaning and analysis at 5% level of significance and 95% Confidence Intervals (CI). Proportion of risk perceptions and attitudes were computed and presented as percentages while factors associated with risk perception were determined using Fisher exact test. Qualitative data was analyzed using thematic content analysis.<br><br>Results: More than half, 54.2% (253/468), of the respondents were females, mean age was 37 years (SD=13.13, ranging from 18 to 88 years). Half of the respondents, 50.9% (238/467), were farmers by occupation and 40.3% (188/468) had completed upper primary education. Only 5.0% (20/396) of consumers reported a high risk perception towards tomatoes stained with pesticide residues, the rest, 95.0% (376/396), were buying pesticide stained tomatoes despite their awareness about the possible health effects. The main reason for buying the pesticide stained tomatoes was that a majority, 59.0% (230/390), lacked an alternative to stained tomatoes. However, consumers generally had a negative attitude towards pesticide stained tomatoes, with 67.0% (313/468) of the consumers disagreeing to a statement that tomatoes sold on the market are safe. Consumer risk perception was significantly associated with consumer awareness about residues in the tomatoes; where the proportion of consumers who were aware of the risk of pesticide stained tomatoes were 42.8 times more likely not to buy stained tomatoes compared to the proportion of those who were not aware. OR, 42.8 (95% CI: 10.76-170.28). However, after Fisher-Exact tests analysis, level of education P(0.975), gender P(0.581) and age-group P(0.680) were not associated with consumer risk perception (95% CI and 5% level of significance).<br>Conclusion: Consumer risk perception on pesticide stained tomatoes among Ugandan consumers ranked low with majority of the consumers buying tomatoes stained with pesticide residues due to lack of an alternative.
**Order of Authors:**	Daniel Sekabojja
	Aggrey Atuhaire
	Victoria Nabankema
	Deogratias Sekimpi

Powered by Editorial Manager® and ProduXion Manager® from Aries Systems Corporation

	Erik Jórs
**Response to Reviewers:**	We Thank the reviewers for the wonderful comments and insights, they really important in improving this piece of research.
**Additional Information:**	
**Question**	**Response**
**Financial Disclosure**<br><br>Enter a financial disclosure statement that describes the sources of funding for the work included in this submission. Review the submission guidelines for detailed requirements. View published research articles from *PLOS ONE* for specific examples.<br><br>This statement is required for submission and **will appear in the published article** if the submission is accepted. Please make sure it is accurate.<br><br>**Unfunded studies**<br>Enter: *The author(s) received no specific funding for this work.*<br><br>**Funded studies**<br>Enter a statement with the following details:<br>• Initials of the authors who received each award<br>• Grant numbers awarded to each author<br>• The full name of each funder<br>• URL of each funder website<br>• Did the sponsors or funders play any role in the study design, data collection and analysis, decision to publish, or preparation of the manuscript?<br>• **NO** - Include this sentence at the end of your statement: *The funders had no role in study design, data collection and analysis, decision to publish, or preparation of the manuscript.*<br>• **YES** - Specify the role(s) played.<br><br>* typeset	The study received funding from Dialogos a Danish NGO under the Pesticides Use Health and Environment Project (2017-2020). Funding was not received by an individual, but directed and received by the Uganda National Association of Community and Occupational Health as an organization. The study was part of the project to assess the pesticide exposures through food and in no way did the funder influence the carrying out of the study to suit his agendas.<br><br>NO - The funders had no role in study design, data collection and analysis, decision to publish, or preparation of the manuscript.
**Competing Interests**<br><br>Use the instructions below to enter a competing interest statement for this submission. On behalf of all authors, disclose any competing interests that could be perceived to bias this work—acknowledging all financial support	The authors have declared that no competing interests exist.

Powered by Editorial Manager® and ProduXion Manager® from Aries Systems Corporation

and any other relevant financial or non-financial competing interests.<br><br>This statement **will appear in the published article** if the submission is accepted. Please make sure it is accurate. View published research articles from *PLOS ONE* for specific examples.<br><br>**NO authors have competing interests**<br><br>Enter: *The authors have declared that no competing interests exist.*<br><br>**Authors with competing interests**<br><br>Enter competing interest details beginning with this statement:<br><br>*I have read the journal's policy and the authors of this manuscript have the following competing interests: [insert competing interests here]*<br><br>* typeset	
**Ethics Statement**<br><br>Enter an ethics statement for this submission. This statement is required if the study involved:<br><br>• Human participants<br>• Human specimens or tissue<br>• Vertebrate animals or cephalopods<br>• Vertebrate embryos or tissues<br>• Field research<br><br>Write "N/A" if the submission does not require an ethics statement.<br><br>General guidance is provided below. Consult the submission guidelines for detailed instructions. **Make sure that all information entered here is included in the Methods section of the manuscript.**	This study was submitted to the ethical review board of Makerere University school of public health (Higher Degree Ethical Research Committee -HDERC) and approved with registration number.<br>Informed consent was obtained from all participants verbally, but also written on the informed consent forms.

Powered by Editorial Manager® and ProduXion Manager® from Aries Systems Corporation

**Format for specific study types**<br><br>**Human Subject Research (involving human participants and/or tissue)**<br>• Give the name of the institutional review board or ethics committee that approved the study<br>• Include the approval number and/or a statement indicating approval of this research<br>• Indicate the form of consent obtained (written/oral) or the reason that consent was not obtained (e.g. the data were analyzed anonymously)<br><br>**Animal Research (involving vertebrate animals, embryos or tissues)**<br>• Provide the name of the Institutional Animal Care and Use Committee (IACUC) or other relevant ethics board that reviewed the study protocol, and indicate whether they approved this research or granted a formal waiver of ethical approval<br>• Include an approval number if one was obtained<br>• If the study involved *non-human primates*, add *additional details* about animal welfare and steps taken to ameliorate suffering<br>• If anesthesia, euthanasia, or any kind of animal sacrifice is part of the study, include briefly which substances and/or methods were applied<br><br>**Field Research**<br><br>Include the following details if this study involves the collection of plant, animal, or other materials from a natural setting:<br>• Field permit number<br>• Name of the institution or relevant body that granted permission	
**Data Availability**<br><br>Authors are required to make all data underlying the findings described fully available, without restriction, and from the time of publication. PLOS allows rare exceptions to address legal and ethical concerns. See the PLOS Data Policy and FAQ for detailed information.	Yes - all data are fully available without restriction; Yes - all data are fully available without restriction

A Data Availability Statement describing where the data can be found is required at submission. Your answers to this question constitute the Data Availability Statement and **will be published in the article**, if accepted.<br><br>**Important:** Stating 'data available on request from the author' is not sufficient. If your data are only available upon request, select 'No' for the first question and explain your exceptional situation in the text box.<br><br>Do the authors confirm that all data underlying the findings described in their manuscript are fully available without restriction?	
**Describe where the data may be found in full sentences. If you are copying our sample text, replace any instances of XXX with the appropriate details.**<br><br>• If the data are **held or will be held in a public repository**, include URLs, accession numbers or DOIs. If this information will only be available after acceptance, indicate this by ticking the box below. For example: *All XXX files are available from the XXX database (accession number(s) XXX, XXX.*).<br>• If the data are all contained **within the manuscript and/or Supporting Information files**, enter the following: *All relevant data are within the manuscript and its Supporting Information files.*<br>• If neither of these applies but you are able to provide **details of access elsewhere**, with or without limitations, please do so. For example:<br><br>*Data cannot be shared publicly because of [XXX]. Data are available from the XXX Institutional Data Access / Ethics Committee (contact via XXX) for researchers who meet the criteria for access to confidential data.*<br><br>*The data underlying the results presented in the study are available from (include the name of the third party*	All relevant data are within the manuscript and its Supporting Information files. All relevant data are within the manuscript and its Supporting Information files.

Powered by Editorial Manager® and ProduXion Manager® from Aries Systems Corporation

and contact information or URL).<br>• This text is appropriate if the data are owned by a third party and authors do not have permission to share the data.<br><br>* typeset	
Additional data availability information:	;

Powered by Editorial Manager® and ProduXion Manager® from Aries Systems Corporation

Manuscript

Click here to access/download;Manuscript;Revised manuscript (clean copy).docx

Consumer risk perception towards pesticide stained tomatoes in Uganda.

[1*]Daniel Sekabojja, [1]Aggrey Atuhaire, [1]Victoria Nabankema, [1]Deogratias Sekimpi, [2,3,4]Erik Jors

[1]Uganda National Association of Community and Occupational Health (UNACOH)

[2]NGO Diálogos, Copenhagen, Denmark

[3]Clinical Institute, Faculty of Medicine, University of Southern Denmark

[4]Clinic of Occupational Medicine Odense University Hospital, Denmark

*Corresponding author

Email: sdaniel199@gmail.com (DS)

Author contribution

[1*]Designed the study, collected data, analyzed data and merged the manuscript together,

[1&234] contributed equally to; conceptualization of the study, methodology, analysis plan, manuscript review and editing.

Membership list of supporters is provided in the acknowledgement section

Abstract

Background: Tomatoes are consumed daily. Unfortunately, abuse of pesticides application by vegetable growers in Uganda increases risks to pesticide residues exposure among consumers, as they may be above European Union Maximum Residue Limits (used as

a standard in Uganda). This study was aimed to determine consumer attitudes and risk perception towards pesticide stained tomatoes in Uganda.

Methods: A mixed methods cross-sectional study sampled 468 household consumers in four regions of Uganda, in each region selecting one district (project interventional area). In each district, 60 household members from three randomly selected Sub Counties were interviewed in addition to 9 tomato handlers (3 tomato farmers, 3 tomato retailers and 3 tomato wholesalers) who participated in Focus Group Discussion (FGDs) per district. Collected data was entered into MS-Excel 13 and exported into STATA SE 14.0 for cleaning and analysis at 5% level of significance and 95% Confidence Intervals (CI). Proportion of risk perceptions and attitudes were computed and presented as percentages while factors associated with risk perception were determined using Fisher exact test. Qualitative data was analyzed using thematic content analysis.

Results: More than half, 54.2% (253/468), of the respondents were females, mean age was 37 years (SD=13.13, ranging from 18 to 88 years). Half of the respondents, 50.9% (238/467), were farmers by occupation and 40.3% (188/468) had completed upper primary education. Only 5.0% (20/396) of consumers reported a high risk perception towards tomatoes stained with pesticide residues, the rest, 95.0% (376/396), were buying pesticide stained tomatoes despite their awareness about the possible health effects. The main reason for buying the pesticide stained tomatoes was that a majority, 59.0% (230/390), lacked an alternative to stained tomatoes. However, consumers generally had a negative attitude towards pesticide stained tomatoes, with 67.0% (313/468) of the consumers disagreeing to a statement that tomatoes sold on the market are safe. Consumer risk perception was significantly associated with consumer awareness about residues in the tomatoes; where the proportion of consumers who were aware of the risk of pesticide stained tomatoes were

42.8 times more likely not to buy stained tomatoes compared to the proportion of those who were not aware. OR, 42.8 (95% CI: 10.76-170.28). However, after Fisher-Exact tests analysis, level of education P(0.975), gender P(0.581) and age-group P(0.680) were not associated with consumer risk perception (95% CI and 5% level of significance).

Conclusion: Consumer risk perception on pesticide stained tomatoes among Ugandan consumers ranked low with majority of the consumers buying tomatoes stained with pesticide residues due to lack of an alternative.

Key words: Consumer Risk Perception, Food Safety, Pesticide Residues.

Introduction:

Globally, there has been an increase in the inquiry on the knowledge of dangers of chemicals in food which has aroused consumer concerns on food safety(1, 2). This follows from consumer reports on health effects of pesticides from their inappropriate use, exposing consumers to high amounts of pesticide residues in harvested foods (3-5). Pesticide residues in food are directly related to the irrational application of pesticides on growing crops and to a lesser extent from residues remaining in the soil. Accumulated pesticide residues in food products are associated with human health hazards ranging from acute to chronic toxic effects (4, 6-8).

In the developing countries, almost all fruits and vegetables grown on a commercial scale are sprayed with pesticides to combat pests and diseases. A study done in 2014 at the two largest horticultural produce markets in Africa showed that 91% of the fruit and vegetable samples collected between 2012 and 2014 had pesticide residues although these were compliant with the Maximum Residue Limits (MRLs) (9).

For numerous decades, the use of pesticides has intensified globally in agriculture, homes and industries aiming at increasing productivity and reducing on the losses (4, 10-13). In the Sub Saharan Africa with tropical climate that favors the growth and rapid multiplication of pests, pesticides are usually used at all levels of agricultural production including on farms to shield plants from pest attack and damage, to control weeds and parasites in livestock as well as in post-harvest control measures. It is now nearly impossible to produce food in tropical regions without using agro-chemicals, however, considerations have been made in the climate change mitigation strategies (14).

In most low-income countries like Uganda, pesticide regulation enforcement and support of the agricultural extension staff to guide farmers on pesticide application standards and dosage, is essentially lacking. Fresh produce sold at local markets is not usually analyzed for agricultural chemical residues unlike export products. This raises concerns about the perceived safety levels of local food supplies in contrast to exported products (9). For instance, a study in Uganda showed that 24.5% of farmers were not aware of any health risks of spraying tomatoes close to harvest time, almost 50% of farmers (45.8%) sprayed their tomatoes less than a week to harvest time, 29.2% sprayed their tomatoes on harvesting, with intentions to extend the shelf-life while 50% did so to attract consumers (15, 16).

Another study in 2015, shows how farmers sprayed tomatoes 6 times the manufacturer recommended dosage, and harvested these tomatoes 2-3 days after last spraying session compared to recommended pre-harvest interval of 4-7days (16). These phyto-sanitary practices increase pesticide residues in tomatoes, if there are no control measures in place as Uganda lacks a pesticide residue monitoring plan for conventionally grown food, and given that the health sector has not prioritized prevention and control measures for pesticides poisoning (17).

Although developed countries use 75% of global pesticides; these countries apply them with strict regulations compared to developing countries which lack the regulation enforcement. Although developing countries use the least quantities of pesticides, they use the most toxic ones (18, 19) resulting in increased risks of acute poisoning. The inappropriate use of pesticides in developing countries increases pesticide exposure and health risks to the consumers. Approximately 25% of developing countries lack regulations and 50% of the WHO-region countries lack sufficient resources to enforce their pesticide-related regulations (20-22). Also, under existing international laws, highly toxic, banned or

unregulated pesticides are always exported to developing countries (23-26), posing health risks to consumers.

Although Uganda is in its transition of establishing a pesticide residue monitoring program, this has moved on a slow pace and made protection of public health unrealized. This study therefore aimed at understanding the risk perception of consumers towards pesticide stained tomatoes, the associated factors, as well as their attitudes towards pesticide stained tomatoes to justify the need for a pesticide residue monitoring program in Uganda.

Methods

Study area and population

This study was conducted in 4 districts as project interventional areas, each selected from one of the four regions of Uganda namely; Northern (Nebbi), Western (Masindi), Eastern (Bugiri) and Central (Sembabule). From each district, three sub counties were randomly selected and consumers sampled systematically at household level for interviews. The above districts were Pesticide Use, Health and Environment (PHE) Project intervention areas with a tropical climate where tomatoes, cabbages, passion fruits, oranges, mangoes, okra, green pepper, amaranths, eggplant, among others, are commonly grown and intensively sprayed with pesticides and agriculture was a day to day business. The commonly used method of pesticide application is a knapsack sprayer which is worn as a backpack and mechanically operated with a hand pump.

The Uganda National census 2014 estimates average population for the above districts as follows; Nebbi (385,220), Masindi (94,622), Bugiri (426,000) and Sembabule (219,600).

Study design

A cross-sectional study design conducted in June 2019 employed mixed methods of both qualitative and quantitative data gathering. Working through already existing branches of the District Farmers Association (DFA) in each of the four districts, three sub counties were randomly sampled clustered into urban, peri-urban and rural. In each of the districts, at household level, at total of 468 participants were randomly selected and interviewed from the four districts as follows; 117 participants from Nebbi District, 102 participants from Sembabule district, 119 participants from Bugiri district and 130 participants from Masindi district. In addition, purposive sampling for Focus Group Discussions (FGDs) was done by the DFA focal person, forming a total of 9 participants per group per district. Each FGD was composed of 3 tomato farmers, 3 tomato retail vendors and 3 tomato wholesalers totaling to 36 participants for the four districts.

Materials

Pretested and standardized structured questionnaires adopted from a survey "*A monitor on consumer confidence in food safety*" developed (by Janneke de Jorge, 2008) for monitoring consumer safety in a Canadian population were modified and used for data collection (details provided in the S5 File). All questionnaires were translated into the participant local languages and translated back into English for quality assurance purposes. Focus Group Discussion Guides were used to collect the qualitative data and also administered in the

local language by trained Research Assistants (RAs). (Details of the guides are provided in S6 File).

Consumer risk perception was assessed using a series of three questions in the order; 1) Are pesticide residues harmful to human health? 2) Are you aware that tomatoes sold on local markets contain pesticide residues? 3) Do you buy pesticide stained tomatoes? Attitudes were measured on a three-Likert scale (responses ranging from agree, not sure and disagree). Questionnaire included three sections on optimism, pessimism and trust. Under the optimism section, questions assessed the safety, confidence and satisfaction about the pesticide residues on tomatoes. Section on pessimism assessed the worrisomeness, suspicion and discomfort caused by the pesticides residues on the tomatoes, while section on trust assessed the consumer's trust in whether the tomato vendors had the characters of trust such as the competence to control safety of tomatoes, the knowledge to guarantee tomato safety, honesty about the safety of the tomatoes, sufficiently open about tomato safety and giving special attention to control the safety of tomatoes (Assessment results provided in S2 File).

Data collection and analysis

Research Assistants (RAs) were trained on the objectives of the study in a one day training per district, questionnaire were pretested with the RAs and supervision done on a daily basis, every filled-in questionnaire was reviewed to ensure that they were fully filled in and that ethical considerations were not bleached.

Trained RAs from the respective District Farmer Associations were enumerated to gather quantitative data in each of the 3 sub counties per district, and one RA per district assisted in doing translations during FGDs for qualitative data collection. A total of 36 respondents

were involved in the FGDs, 9 per district, all their responses recorded on audio and data gathered on tapes. Sample size for the FGDs was based on the level of saturation of the responses.

Quantitative Collected data was gathered and entered into Microsoft Excel Version 2013 and exported into Stata Corp version14 for cleaning and analysis. A total of 468 entries were achieved. Categorical variables like Risk Perception (measured as a binary outcome, high risk or low risk perception) and Attitude, age group, occupation, gender, and level of education were presented as frequencies with their respective percentages, while continuous variable such as age presented as means with their respective (SD) and ranges.

Bivariable analysis for risk perception (measured as a binary outcome), was computed by gender, level of education, age categories, residence (rural, urban and peri-urban) and the chi-square plus the respective p-values reported under (95% CI, 5% Level of significance). Awareness about the pesticide residues was computed by level of education and by the practice of buying tomatoes and their Chi-square, p-values under (95% CI and 5% Level of significance) reported. Fisher exact test was used to determine the factors associated with consumer risk perception and the factors for buying of pesticide stained tomatoes under (95% CI, 5% Level of significance), details provided in the S3 File. Finally, simple logistic regression was used to compute the odds of high risk perception by consumers who were aware of pesticide residues on the tomatoes and their respective Odds Ratio with the p-values reported with 95% CI and 5% Level of significance.

Qualitative data collected among the 36 participants in the four districts was transcribed and analysis done thematically based on the study objectives and conclusions based on participant responses. These conclusions were later used to support a discussion with quantitative data findings.

Ethical consideration

This study sought ethical approval from Makerere University School of Public Health, Higher Degree Research and Ethics Committee (MakSPH HDREC) with an approval reference registration number 686. Informed consent was sought from all participants before the interviews, for anonymity, participants initials were used instead of their names on the questionnaire and participants were free to withdraw from the study at any point when they felt like not continuing with the interviews.

Results

Demographic characteristics of consumers

The study registered all response from consumers, equally sampled by residence (rural, urban and peri-urban). From the three sub counties in each of the four districts of Uganda; (Northern region: Nebbi district, Eastern Region: Bugiri District, Central Region: Sembabule district and Western region: Masindi district).

As indicated in Table 1, Slightly more than half, 54.1% (253/468), of respondents were females, a majority, ≈51.0% (238/468) practiced farming as an occupation (*refer to S1* File), a majority, 84.4% (273/468), had attained lower level of education. The mean age of participants was 37.7years (SD±13.1, ranging from 18-88) with majority 54.7% (256/468) belonging to the age group below the mean age. From the qualitative results, interviews involved categories of tomato farmers 33.3% (12/36), tomato retail vendors 33.3% (12/36), and tomato wholesalers 33.3% (12/36) sampled in equal proportions in the 4 districts. i.e. three persons, per category, per district.

Table 1: Demographic characteristics of consumers

Variable	Category	Frequency (%)
Gender	Male	214 (45.8)
(n=468)	Female	253 (54.2)
Age group	Below mean Age	256 (54.7)
	Above mean Age	212 (45.3)
	**Mean Age**	37.7 (SD±13.1)

Level of education<br><br>(n=468)	No formal education<br><br>Lower level<br><br>upper level	39 (8.3)<br><br>395 (84.4)<br><br>34 (7.3)

General consumer attitudes towards pesticide stained tomatoes

Consumer negative attitude (pessimism) towards pesticide stained tomatoes.

Basing on the percentages of pessimism, measured on a 3-Likert scale, a majority, 74.3%(347/467) of the consumers had a negative attitude (on the agreement side) towards the stains on the tomatoes compared to 2.4%(11/467) who were not sure and 23.5% (109.6/467) who felt positive about the stains on the tomatoes in terms of worrisomeness, discomfort and suspicion caused by the residues. (Details provided in S2 File and Fig1).

Fig 1. Average negative perceptions of consumers on pesticide stained tomatoes in percentage.

Consumer positive attitude (optimism) towards pesticide stained tomatoes.

Basing on the percentages of optimism, measured on a 3-Likert scale, Consumer positive attitude towards the pesticide stains was low. On average, only 33.2% (155.3/468) of the consumers agreed to the statement that tomatoes sold on the Ugandan market are safe,

compared to a majority 61.1%(285.6/468) who disagreed with the statement while 5.7% (26.7/468) were not sure as provided in Fig2. and indicated S2 File.

Fig 2: Average positive perceptions of consumers on pesticide stained tomatoes in percentages.

Consumer level of trust in pesticide stained tomatoes

In terms of trust, based on the 3-scale resized averages for trust, about 77.7 % (362/466) of consumers lacked trust and disagreed that tomato vendors have the characters of trust such as the competence to control safety of tomatoes, the knowledge to guarantee tomato safety, honesty about the safety of the tomatoes, sufficiently open about tomato safety and gave special attention to control the safety of tomatoes compared to 8.1% (37.6/466) who were not sure and 14.1%(65.4/466) who trusted tomato vendors (details provided in the S2 File).

Consumer risk perception level towards pesticide stained tomatoes

Consumer risk perception was measured using a model with questions as provided in Fig 3.

Fig 3. Model for determining consumer risk perception

Consumer risk perception

Among consumers who were aware and knowledgeable about sold tomatoes containing pesticide residues, ≈95.0% (376/396) of them bought these pesticide stained tomatoes (i.e. had a low risk perception) compared to only 5.0% (20/396) who perceived tomatoes to be of high risk to their health and withdrew from buying them (i.e. had a high risk perception) as provided in Fig 4.

Fig 4. Level of consumer risk perception towards pesticide stained tomatoes

Reasons for buying pesticide stained tomatoes.

The main reasons for buying pesticide stained tomatoes was that, 59.0% (230/390) of the consumers had no alternative choice to buying pesticide stained tomatoes, followed by 27.2% (106/390) who had to prepare these tomatoes to reduce on the residues, 9.2% (36/390) who perceived no health risks of buying stained tomatoes and 4.6% (18/390) falling in the other categories which included the preference of tomato attributes like size, ripeness among other than the pesticide residues.

Consumer confidence on the safety of tomatoes sold on Ugandan Market.

On a general scale, more than half, 66.9% (313/468), of the consumers disagreed to the fact that tomatoes sold on the Ugandan markets are safe, consumers' general confidence on the safety of tomatoes sold on Ugandan Markets outweighed their counterparts with nearly half, 49.6%(231/466), of the consumers being confident about the safety of tomatoes sold on the Ugandan markets while only 14.4%(168/466) were not confident and 14.4% (167/466) were not sure.

Factors associated with consumer risk perception and buying of stained tomatoes.

From the Fisher-exact tests, Consumer risk perception was not associated with the level of education P(0.975), Residence P(0.462), gender P(0.581), age-group P(0.680) and marital status P(0.581), as indicated in the S3 File. Further simple logistic regression analysis revealed that consumer risk perception was significantly associated with consumer awareness about residues in the sold tomatoes; where the proportion of consumers who were aware that tomatoes contain pesticide residues being 42.8 fold more of a high risk perception compared to the proportion of those who were not aware of the residues as provided in the S4 File.

In table 2, the consumer awareness about Pesticide residues was not associated with age-group and level of education but significantly associated with gender, where male consumers were 1.77 time more likely to be aware of the pesticide residues in the tomatoes compared to counter parts; the second factor was whether consumers ever obtained pesticide safety information where consumers who had never obtained pesticide safety information being 61% less likely to be aware of the pesticide residues compared to consumers who had obtained information on pesticide safety, OR 0.39 (95% CI: 0.24-0.64)

Table 2: Logistic Regression for Consumer awareness about pesticide residues on tomatoes and some consumer demographics (crude Odds ratio)

	**Aware of pesticide residues**<br>**No (%) Yes (%)**	**Odds Ratio**	**Std. Err.**	**P>|z|**	**[95% CI]**	
**Gender**						
Female	54(21.3) 200(78.7)	1.0				

Male	28(13.2) 184(86.8)	1.77	.451	**0.024**	1.078	2.921
**Age category**						
Below mean age	46(18.0) 210(82.0)	1.0				
above mean age	36(17.1) 174(82.9)	1.06	.259	0.816	.655	1.711
**Education category**						
None	10(25.6) 29(74.4)	1.0				
Lower level Education	69(17.6) 324(82.4)	1.62	.631	0.217	.754	3.477
Upper level Education	3(8.8) 31(91.2)	3.56	2.52	0.072	.891	14.249
**Ever obtained information about pesticide safety**						
Yes	34(12.1) 247(87.9)	1.0				
No	48(25.9) 137(74.1)	0.39	.098	**0.000**	.244	.639

Consumer awareness about the pesticide stained tomatoes was not associated with consumer level of education P(>0.05) but significantly associated with consumer risk perception P(<0.05) and the practice of buying stained tomatoes P(<0.05) as indicated in table 3 below.

Table 3: Showing the fisher-exact tests for awareness about pesticide residues in tomatoes verses the consumer level of education and practice of buying stained tomatoes.

**Buy stained tomatoes**	**Aware of pesticide residues in the tomatoes Freq (%)**			**Fishers-exact p-values**
	**Yes**	**No**	Not sure	**0.000**
No	14/20(70.0)	6/20(30.0)	0 /20(0.0)	

Yes	370/376(98.4)	4/376(1.1)	2/376(0.5)	
**Consumer risk perception**				**0.000**
High risk perception	13/19(68.4)	6/19(31.6)	0/19(0.0)	
Low risk perception	371/377(98.4)	4/377(1.1)	2/377(0.5)	
**Education level**				0.095
None	29/39(74.4)	7/39(17.9)	3/39(7.7)	
primary	216/272(79.4)	35/272(12.9)	21/272(7.7)	
secondary	108/121(89.3)	7/121(5.8)	6/121(5.0)	
tertiary	31/34(91.2)	1/34(2.9)	2/34(5.9)	

Qualitative findings

Theme	Main claim	Underlying issue	Preposition	Argument/disagreement
**Pesticide effects on human health**	From our qualitative findings, most participants, 41.2% (7/17) claimed that pesticide stained tomatoes are poisonous, harmful to human health and create fear for the consumer's health. *"To me it is real poison because even on the pesticide label the manufacturer cautions the users to put on protective gears. This puts me at risk as a farmer and puts fears to the final consumer that is not safe", one of the Farmers from Masindi district said. These residues are harmful to our health because from the time of planting to harvesting a farmer uses over 10 chemical types in order to bring out good results meaning that these chemicals get dissolved inside the tomato juice", one of the wholesaler from Sembabule district said.*	Some participants claimed that spraying tomatoes with pesticides was fine as long as they are washed before eating. However, some claimed that spraying high dosages leads to more residues/stains on tomatoes which may affect the consumers. Some participants were of the opinion that residues indicate high doses of pesticide spraying before sale to the market rendering tomatoes unsafe for consumption.	Participants mainly suggested that tomatoes need to be washed before sale in the market and consumption to ensure safety, while other participants argued that tomatoes can be soaked in water for one (1) hour before consumption or the outer cover peeled off in order to reduce exposure in consumed food.	However, there was a diversion in perception about the harmfulness of pesticides, Some Participants argued that Pesticides are only harmful to pests on plants and not humans, they protect tomatoes from damage, keep them safe and ready for consumption, "*Since the pesticides are used to kill pests, the chemical is only harmful to the pests affecting tomatoes.*" narrated by a farmer from Olyeko village, Nebbi municipality.<br><br>Some participants were more interested in the appearance of pesticide residues/stains on tomatoes claiming that it assured them of the market since customers preferred such tomatoes. Participants discussed that the residues are both on top and inside, preserve the tomatoes and reduce losses and customers had to wash the tomatoes clean of pesticides. Participants also

				mentioned that they spray because of environmental related issues claiming that the environment can no longer support proper tomato growth without use of pesticides and Pesticides are used to increase yields. Unfortunately, participants were aware that farmers don't follow instructions on labels and rarely use any protective equipment as these are not easily accessible and affordable.

**Perception of residue and impact on consumer health**	Most of the Participants 47.1% (8/17) said that pesticides negatively affect consumer health. This is mainly because most of them had experienced a sign of pesticide poisoning, for instance some claimed itching, irritations, stomachaches, and restlessness. Some participants were aware of the effects from the pesticide labels while some claimed that the smell of pesticides indicates that they have a negative effect on health.<br><br>Most participants 70.6% (12/17) claimed pesticides were harmful due to the health problems they cause whereas some, 29.4% (5/17) were somehow optimistic and argued that pesticide residues are not very harmful because the effects are observed after a very long time.	Some participants proposed that Consumers need to be sensitized on pesticide dangers, including the effects of the residues.<br>Some participants argued that due to the low market prices and limited resources, farmers tend to delay harvesting tomatoes by spraying them to keep them longer until there is a better market price.	Despite the impact, some participants argued that Farmers overdose tomatoes with pesticides to meet of customers' demand of storing tomatoes for a longer time. Customers in market shun pesticides without pesticide residues; they demand those with residues because they believe they are healthy and last longer.<br>Some attributed this practice to the tomato varieties on market which require a lot of spraying throughout the growth stages to maximize yields.<br>Participants argued that farmers can't interpret the labels on pesticide containers because most of them are not educated and therefore may overdose or under dose the tomatoes with pesticides.<br>In addition, some participants claimed that pesticides are not very bad if used properly but farmers don't observe pre-harvest interval.<br><br>Some participants recommended that the government plays its role to obliterate some pesticides including counterfeits from the market, while others recognized that farmers also have a role to play by following the prescription made by the manufacturer from the labels.	
**Considerations before**	Some participants claimed they don't pay attention to pesticide residues and buy	Participants recommend that		Some participants claimed that tomatoes are

**buying tomatoes**	tomatoes with pesticides because they last longer. Some participants consider tomato size (prefer big to small), tomato ripeness (prefer not so ripe tomatoes), freshness and customer needs.<br>"*We look for general appearance of tomato but at sight I look at the pesticide residue on tomato not the size. I would better go with small tomato with pesticide than a big one.*" answered a farmer from Masindi district.<br>However, a few wholesalers consider Place of tomato selling. They say that usually tomatoes from non-mulched gardens spoil faster especially during rainy seasons. So they would like to make loses by discarding most of the damaged tomatoes. "*Sure deal, traders consider the pesticide residues on the tomato, and the quality of the tomatoes is determined by the life expectancy of that tomato. A good tomato turns red not yellow in appearance and doesn't have disease spots when ready. Traders consider the glittering cover, and size. For tomatoes sprayed from the store, the residue is just on top and can easily be rubbed off with your mere hands but a tomato which has been sprayed earlier can't rub off the residues even if they are being seen.*" explained a tomato whole seller from Masindi district.<br>"*When the buyers are many in his garden they don't*	Researchers share information with local people to understand the health impact of pesticides and how to reduce exposure.		sprayed shortly before harvesting to stores, because some buyers may book the tomatoes and fail to pick up on the exact promised day.<br>All Participants however, claimed no further spraying is done at the stalls because they are already sprayed well and need to preserve quality and harden skin.

	consider or mind anything they just collect all except the damaged ones" said a farmer from Nebbi district.			

Discussion

Very little information is available about the pesticide residue in tomatoes in Uganda and consequently the risk of exposures to this. The focus of this study was to determine the consumer risk perception towards pesticide stained tomatoes and pesticide residues, and the attitudes (pessimism vs. optimism) towards the safety of the pesticide stained tomatoes. It employed a cross-sectional study design with a total of 468 consumers as respondents equally sampled by residence and interviewed from each of the four districts (Northern region: Nebbi district, Eastern Region: Bugiri District, Central Region: Sembabule district and Western region: Masindi district), thus a good representation of Ugandan consumers.

Characteristics of the respondents show that slightly more than half of respondents were females as expected since the majority of these stay home to take care of home chores, similar to findings by (27) where 58% of women were involved in the purchase of meat, more than three quarters were married women as interviews were conducted at homesteads and consent sought from adults making it more likely for the married women to be interviewed. Half of the respondents were farmers given that Uganda as a country has nearly three quarters of its population engaged in farming, and also this study was carried out in a rural setting, involving vendors, buyers and tomato growers. A majority had completed lower level of Education and a majority belonged to age group <30 years as the Ugandan population is composed of 75% youths as the majority(28), 80% of these residing in

rural areas(29). From these characteristics we can definitely report that the consumers interviewed in this study were a good representation of tomato consumers in Uganda(28). Respondents of this study represent majority adult female consumers experienced with tomato farming and with adequate level of education (majority with Ordinary level of education) to express well their risk perception towards pesticide stained tomatoes.

Consumer risk perception ranked low with a majority of consumers (95%) buying tomatoes what is well known to be stained with pesticide residues, a majority giving reasons that they have no alternative. Results from this study highly deviate from similar studies conducted in developed countries such as among Californian consumers where 80% were safety cautious and checked the food items to see if they were opened or damaged (30). While in Georgia, 89% considered testing of pesticide residues in food to be very important or somewhat important(31) and in Boston, consumers had a high risk perception of conventionally grown produce compared to public health hazards(32), most of these were mainly triggered by the health effects of the contaminants such as pesticide residues and biological contaminants in the sold produce.

From the Turkish perspective, consumers' willingness to pay for reduced pesticide residues in tomatoes was mainly determined by their risk perception about the residues which is explained by the label on the purchased apples(33). In this case due to the different situations, in Uganda unfortunately tomatoes are not labeled with the residual contents and benefits of low pesticide residual levels. This could be the reason why a majority of the consumers in this study bought the tomatoes. The vegetables on the market lack such information which could have triggered consumers to make their choices. This is a gap to be closed by authorities in charge of food safety. Uganda lacking a stand-alone and comprehensive food safety policy puts public health at stake for the pesticide exposures.

Results from this study are a vivid evidence to be used as part of the advocacy statements in finding ways of establishing a stand-alone National Food Safety Policy.

A study on consumers' willingness to pay for pesticide-free vegetables indicated how consumer awareness about the residues in vegetables and the residual effects on human health greatly influenced their willingness to pay for these vegetables. Consumers in this study were willing to pay 50% more for the pesticide-free vegetables (34). In the Ugandan context, the consumer's low risk perception on pesticide-stained tomatoes indicates a risk of increasing exposures to pesticide residues if these lack an alternative. As reported in other findings (35), our qualitative results from vendors and tomato farmers from the FGDs, indicate that the highly stained tomatoes are due to poor hybrid tomato seeds that need frequent spraying and vendors demand to spray these tomatoes to spray tomatoes before sell. But also a low level of literacy to understand the pesticide label information coupled with a wrong perception of tomato farmers that, Mancozeb pesticide can harden the outer skin and increase tomato shelf life. It is from this misconception that tomato vendors only buy stained tomatoes presuming that these tomatoes will stay long on shelf, are healthy & free from the microbial contaminants.

Unlike other studies(31) from Georgia, where consumers prioritize microbial contamination followed by pesticide residues as the first consideration before making a choice to buy vegetables, tomato consumers in Uganda partly have a feeling that pesticide stained tomatoes are free from pathogens and thus healthy, giving not much priority to pesticide residues. As reported some think that pesticides are selective and only meant to kill plant pests and cure plant diseases. However, our logistic regression analysis indicates how consumer awareness about pesticide residues increases chances of a high risk perception, protecting consumers from residue exposure, although this is just for a few individuals.

From our focus group discussion of vendors and farmers, all farmers claimed that vendors would only buy tomatoes with pesticide residues as these are thought healthy. On the other hand, vendors attest that stained tomatoes are healthy, look good, take long to go stale and have a high resale value on the market.

From these findings, Ministry of Agriculture Animal Industry and Fisheries needs to sensitize farmers as well as improve coordination and regulations on the sale and use of agro-inputs. Agro-input dealers who are the immediate information providers to the farmers, need to be trained in Pesticide safe-use training. A recent unpublished survey done by UNACOH in 2020, reports that only 6% of the agro-input dealers in 12 districts to have obtained the safe-use training, a training required to be undertaken by all agro-input distributors before opening up their business.

Tomato residues are given less attention by the consumers probably because consumers lack knowledge of the dangers that the residues may impact on their health which in most cases takes time. From a model by Huang Chung (36) estimating the relationship between consumer perceptions, attitudes and behavioral intentions (S7 File), choices (behavioral intentions) on buying food is influenced by perceptions and attitudes, which influence each other in addition to knowledge (information) from personal experience, evaluative criteria and social demographics. All based on the consumer awareness about the pesticide's potential ill effects but also on a larger extent the social economic status of the consumer. Consumers of a higher social economic status are most likely to have a high educational level and consequently easily access all the necessary information about the effects of pesticide residues. These will tend to have a high risk perception about the pesticide stained tomatoes and not likely to buy tomatoes which are stained with pesticides. However, this

was not so with our finding. Consumer risk perception was not directly associated with level of education although associated with awareness about pesticide residues, where consumers who were aware of pesticide residues were 42.8 times more likely to be of high risk perception compared to those who were not aware. From our results the low proportions (5%) of high risk perception consumers may be largely attributed to our sample containing low percentages of highly educated consumers (upper level education, table 1).

On the other hand, pesticide residue knowledge among the general public in Uganda is a new topic and studies conducted along these lines are few, most of the time not intended for creating awareness among the public on the potential ill effects of residues in food. From other related findings in this study, consumers have no access to sources of information on pesticide residues in food with most of the information on pesticide residues acquired through radio and television media, followed by health professionals, depicting a big gap in information accessibility, but also its availability. Basing in the results about misconception that the pesticides residues increases shelf life of a tomato, new studies on the origin of this misconception need to be conducted to challenge the practices of overdosing that pose risks to public health.

Conclusion

Consumer risk perception among Ugandan consumers was ranked low with a majority of tomato consumers buying tomatoes stained with pesticides regardless of their level of education, age and gender, this all linked to lack of alternative organic tomatoes on the market. However awareness that tomatoes contain pesticide residues was associated with consumer risk perception where the proportion of consumers who were aware of tomatoes

containing pesticide residues was 42.8 fold more of high risk perception than consumers who were not aware.

There is a need by the government through its line of Ministry of Agriculture Animal Industry and Fisheries (MAAIF) and other health information dissemination Civil Society Organization to sensitize the Ugandan population on the effects of pesticide residues, the farmers on the dosage and the pre-harvest intervals as well as train Agro-input Dealers from which farmers buy pesticides.

MAAIF should also hasten the establishment of the National Pesticide Residue Monitoring Program to protect Public Health from this chronic exposure to pesticide residues in agricultural produce.

Acknowledgments

The author would like to acknowledge the support from Diálogos participants and all efforts rendered by the Pesticides use, Health and Environment (PHE) Project team, and the respective District Framers' Association with which the study was conducted. DFAs supported highly in questionnaire translation into the respective local languages and in data collection.

Supportive information

Data-set used to yield the above results is available upon request from the author. However, separate supportive information has been attached as highlighted below.

S1 File. Other Demographic characteristics of consumers. PDF

S2 File. Consumer attitudes towards pesticide stained tomatoes. PDF

S3 File. Fisher-exact tests for the factors associated with consumer risk perception. PDF

S4 File. Simple logistic regression of consumer risk perception vs. awareness about pesticide residues. PDF

S5 File. Questionnaire: Consumers risk perception towards pesticide stained tomatoes in Uganda. PDF

S6 File. Focus Group Discussion guide for Consumers risk perception towards Pesticides stained tomatoes in Uganda. PDF

S7 File. A model for estimating the relationship between consumer perceptions, attitudes and behavioral intentions. PDF

Reference

1. Winter CK. Pesticide residues in imported, organic, and “suspect” fruits and vegetables. Journal of agricultural and food chemistry. 2012;60(18):4425-9.
2. Winter CK, Katz JM. Dietary exposure to pesticide residues from commodities alleged to contain the highest contamination levels. Journal of toxicology. 2011;2011.
3. Codex Pesticides Residues in Food Online Database [Internet]. © FAO/WHO, 2019. 2016 [cited 22/02/2019]. Available from: http://www.fao.org/fao-who-codexalimentarius/codex-texts/dbs/pestres/en/.
4. WHO. Pesticide residues in food 2018 [16/04/2019]. Available from: https://www.who.int/en/news-room/fact-sheets/detail/pesticide-residues-in-food.

5. Hamilton D, Ambrus Á, Dieterle R, Felsot A, Harris C, Petersen B, et al. Pesticide residues in food—acute dietary exposure. Pest Management Science: formerly Pesticide Science. 2004;60(4):311-39.
6. Kim K-H, Kabir E, Jahan SA. Exposure to pesticides and the associated human health effects. Science of the Total Environment. 2017;575:525-35.
7. Jones JL, Hanson DL, Dworkin MS, Alderton DL, Fleming PL, Kaplan JE, et al. Surveillance for AIDS-defining opportunistic illnesses, 1992-1997. MMWR CDC Surveill Summ. 1999;48(2):1-22.
8. Hansen MRH, Jørs E, Sandbæk A, Sekabojja D, Ssempebwa JC, Mubeezi R, et al. Exposure to cholinesterase inhibiting insecticides and blood glucose level in a population of Ugandan smallholder farmers. Occupational and Environmental Medicine. 2020.
9. Mutengwe MT, Chidamba L, Korsten L. Monitoring pesticide residues in fruits and vegetables at two of the biggest fresh produce markets in Africa. Journal of food protection. 2016;79(11):1938-45.
10. Wilson C, Tisdell C. Why farmers continue to use pesticides despite environmental, health and sustainability costs. Ecological economics. 2001;39(3):449-62.
11. Zhang W, Jiang F, Ou J. Global pesticide consumption and pollution: with China as a focus. Proceedings of the International Academy of Ecology and Environmental Sciences. 2011;1(2):125.
12. WHO Suicide Fact Sheet [Internet]. 2018 [cited 11/08/2018]. Available from: http://www.who.int/en/news-room/fact-sheets/detail/suicide.
13. Cooper J, Dobson H. The benefits of pesticides to mankind and the environment. Crop Protection. 2007;26(9):1337-48.
14. Majaliwa J, Mukwaya P, Isubikalu P, editors. Climate change adaptation strategies in the semi-arid region of Uganda. 2 nd Ruforum Biennial meeting; 2010.
15. Atuhaire A, Ocan D, Jørs E. Knowledge, Attitudes, and Pratices of Tomato Producers and Vendors in Uganda. Advances in Nutrition and Food Science. 2016;1(1):1-7.
16. Kaye E, Nyombi A, Mutambuze IL, Muwesa R. Mancozeb residue on tomatoes in Central Uganda. Journal of Health Pollution. 2015;5(8):1-6.
17. Sekabojja D, Atuhaire A, Nabankema V, Sekimpi D, Bainomugisa C, Jørs E. Acute Pesticide Poisoning Case Registration in Uganda's Health Care Facilities. Journal of Environmental & Analytical Toxicology. 2020;10(2).
18. Oesterlund AH, Thomsen JF, Sekimpi DK, Maziina J, Racheal A, Jørs E. Pesticide knowledge, practice and attitude and how it affects the health of small-scale farmers in Uganda: a cross-sectional study. African health sciences. 2014;14(2):420-33.
19. Binukumar B, Bal A, Sunkaria A, Gill KD. Mitochondrial energy metabolism impairment and liver dysfunction following chronic exposure to dichlorvos. Toxicology. 2010;270(2-3):77-84.
20. Matthews G, Zaim M, Yadav RS, Soares A, Hii J, Ameneshewa B, et al. Status of legislation and regulatory control of public health pesticides in countries endemic with or at risk of major vector-borne diseases. Environmental health perspectives. 2011;119(11):1517-22.
21. Handford CE, Elliott CT, Campbell K. A review of the global pesticide legislation and the scale of challenge in reaching the global harmonization of food safety standards. Integrated environmental assessment and management. 2015;11(4):525-36.
22. Ogada DL. The power of poison: pesticide poisoning of Africa's wildlife. Annals of the New York Academy of Sciences. 2014;1322:1-20.
23. Phung DT, Connell D, Miller G, Rutherford S, Chu C. Pesticide regulations and farm worker safety: the need to improve pesticide regulations in Viet Nam. Bulletin of the World Health Organization. 2012;90:468-73.

24. Dinham B. Growing vegetables in developing countries for local urban populations and export markets: problems confronting small-scale producers. Pest management science. 2003;59(5):575-82.
25. Ecobichon DJ. Pesticide use in developing countries. Toxicology. 2001;160(1-3):27-33.
26. Smith C, Root D. The export of pesticides: shipments from U.S. ports, 1995-1996. International journal of occupational and environmental health. 1999;5(2):141-50.
27. Spais GS, Vasileiou KZ. An ordinal regression analysis for the explanation of consumer overall satisfaction in the food-marketing context: The managerial implications to consumer strategy management at a store level. Journal of Database Marketing & Customer Strategy Management. 2006;14(1):51-73.
28. UNFPA. UGANDA’S YOUTHFUL POPULATION. 2020.
29. USAID. COMMUNITY HEALTH SYSTEMS CATALOG
COUNTRY PROFILE: UGANDA 2017.
30. Bruhn CM, Schutz HG. Consumer food safety knowledge and practices. Journal of food safety. 1999;19(1):73-87.
31. Misra SK, Huang CL, Ott SL. Consumer willingness to pay for pesticide-free fresh produce. Western Journal of Agricultural Economics. 1991:218-27.
32. Williams PR, Hammitt JK. Perceived risks of conventional and organic produce: pesticides, pathogens, and natural toxins. Risk analysis : an official publication of the Society for Risk Analysis. 2001;21(2):319-30.
33. Akgungor S, Miran B, Abay C. Consumer willingness to pay for reduced pesticide residues in tomatoes: the Turkish case. 1999.
34. Coulibaly O, Nouhoheflin T, Aitchedji C, Cherry A, Adegbola P. Consumers' perceptions and willingness to pay for organically grown vegetables. International journal of vegetable science. 2011;17(4):349-62.
35. Kaaya N. DITHANEM-45 RESIDUES IN TOMATOES ON UGANDAN MARKETS MAY BE ABOVE SAFE LEVELS. African Journal of Food, Agriculture, Nutrition and Development. 2004;4(1).
36. Huang CL. Simultaneous ‐ equation model for estimating consumer risk perceptions, attitudes, and willingness ‐ to ‐ pay for residue ‐ free produce. Journal of Consumer Affairs. 1993;27(2):377-96.

Click here to access/download;Figure;Fig 1.tif.tif

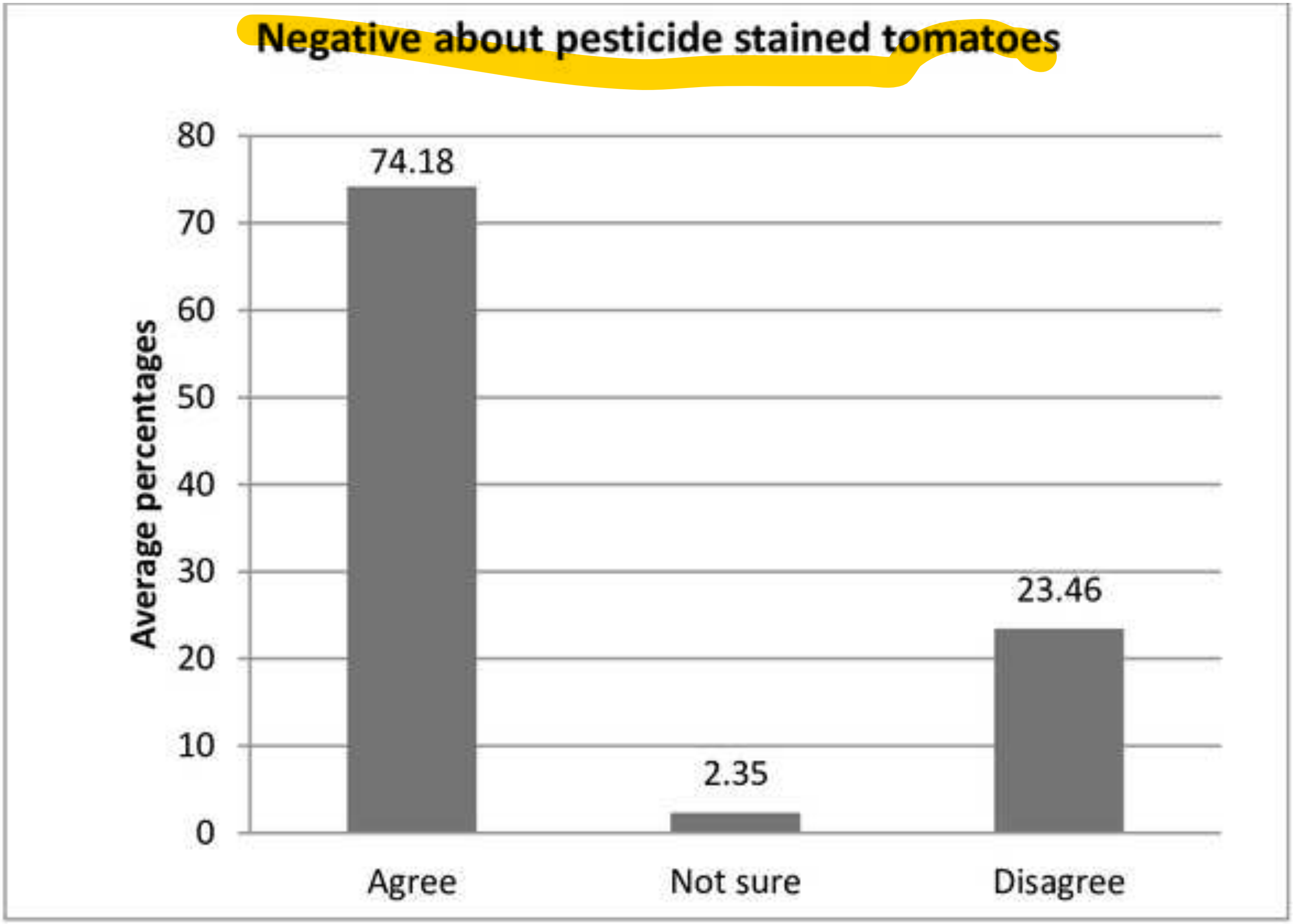

Fig 1. Average negative perceptions of consumers about pesticide stained tomatoes.

 Click here to access/download;Figure;Fig 3.tif.tif

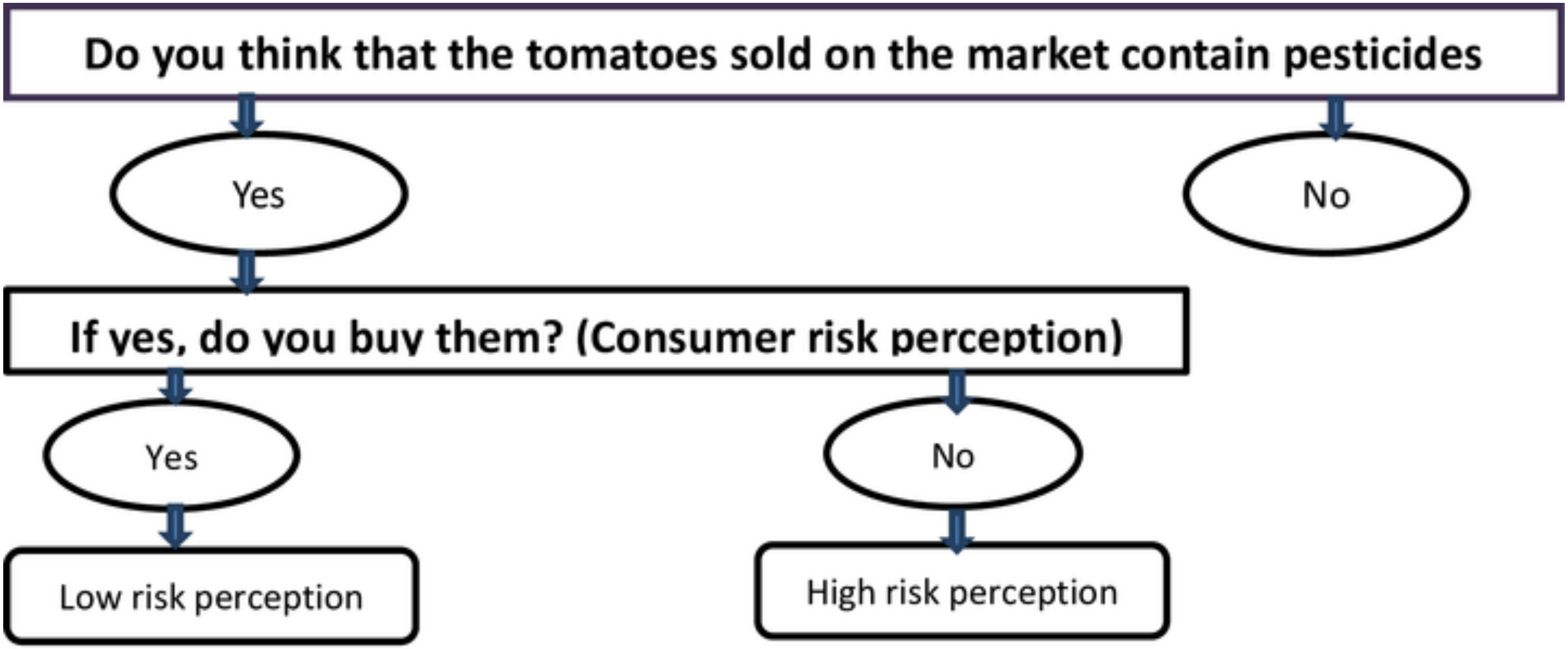

Fig 1. Model for determining consumer risk perception

 Click here to access/download;Figure;Fig 4.tif.tif

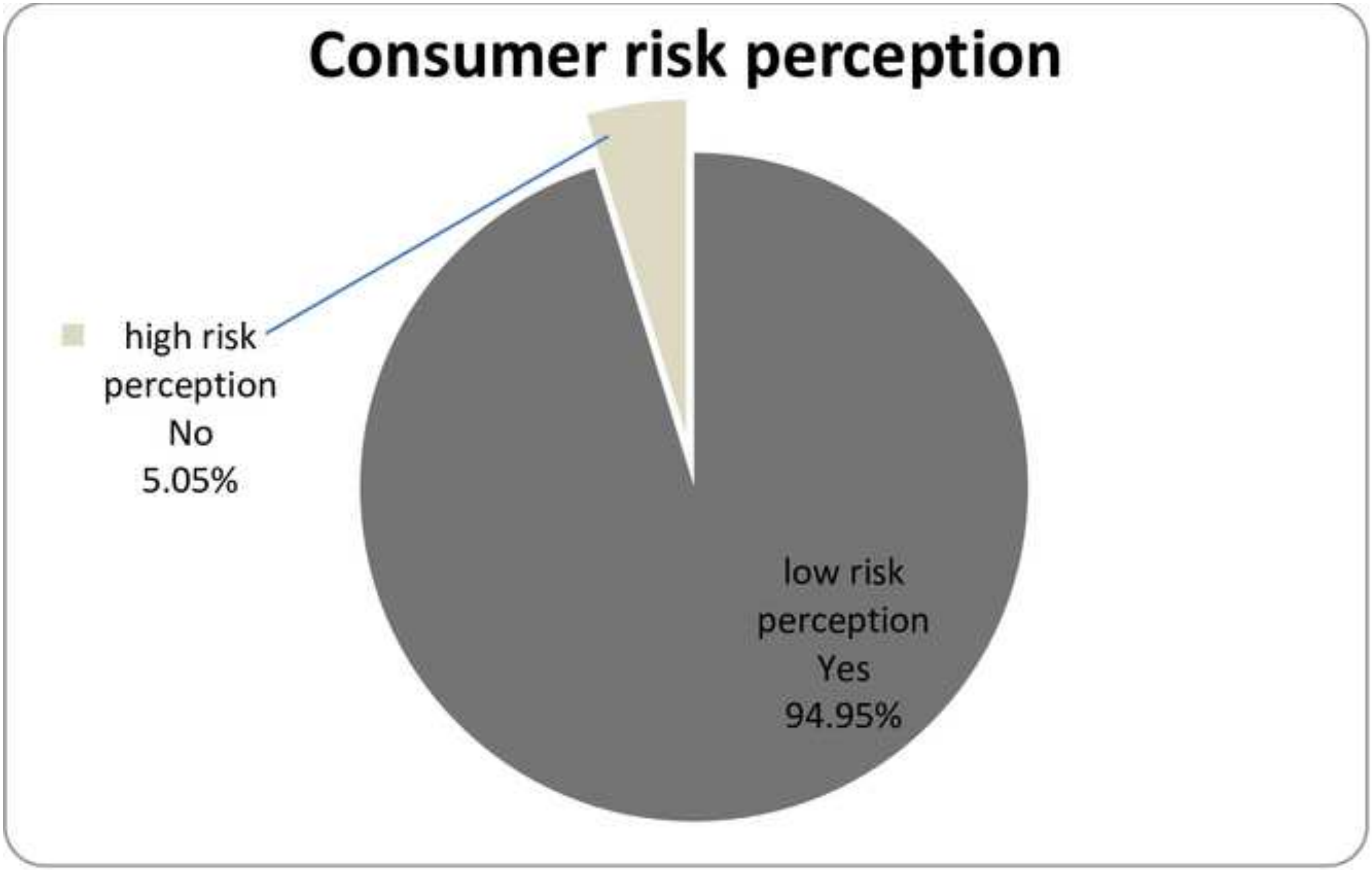

Fig 4. Level of consumer risk perception towards pesticide stained tomatoes

 Click here to access/download;Figure;Fig 2.tif.tif

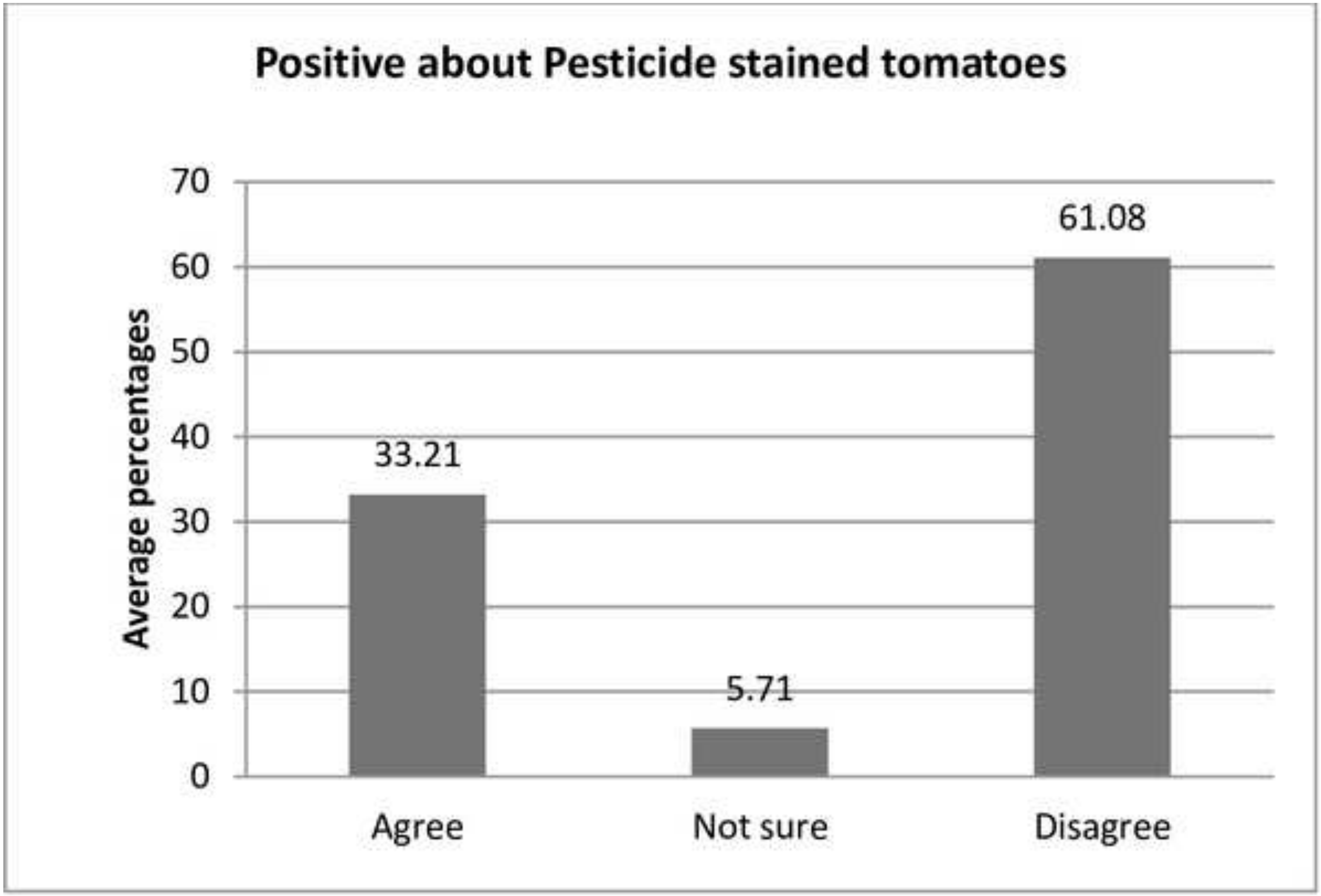

Fig 2. Average positive perceptions of consumers on pesticide stained tomatoes in percentages.

Supporting Information 2

Click here to access/download
Supporting Information
S2 File.pdf

Supporting Information 6

Click here to access/download
Supporting Information
S6 File.pdf

Click here to access/download
Supporting Information
S3 File.pdf

Click here to access/download
Supporting Information
S7 File.pdf

Click here to access/download
Supporting Information
S1 File.pdf

Click here to access/download
Supporting Information
S5 File.pdf

Click here to access/download
Supporting Information
S4 File.pdf

database

Click here to access/download
Supporting Information
consumer riskperception coded database.dta

Consumer risk perception towards pesticide stained tomatoes in Uganda.

[1*]Daniel Sekabojja, [1]Aggrey Atuhaire, [1]Victoria Nabankema, [1]Deogratias Sekimpi, [2,3,4]Erik Jors

[1]Uganda National Association of Community and Occupational Health (UNACOH)

[2]NGO Diálogos, Copenhagen, Denmark

[3]Clinical Institute, Faculty of Medicine, University of Southern Denmark

[4]Clinic of Occupational Medicine Odense University Hospital, Denmark

*Corresponding author

Email: sdaniel199@gmail.com (DS)

Author contribution

[1*]Designed the study, collected data, analyzed data and merged the manuscript together,

[1&234] contributed equally to; conceptualization of the study, methodology, analysis plan, manuscript review and editing.

Membership list of supporters is provided in the acknowledgement section

Abstract

Background: Tomatoes are consumed daily. Unfortunately, abuse of pesticides application by vegetable growers in Uganda increases risks to pesticide residues exposure among consumers, as they may be above European Union Maximum Residue Limits (used as

a standard in Uganda). This study was aimed to determine consumer attitudes and risk perception towards pesticide stained tomatoes in Uganda.

Methods: A mixed methods cross-sectional study sampled 468 household consumers in four regions of Uganda, in each region selecting one district (project interventional area). In each district, 60 household members from three randomly selected Sub Counties were interviewed in addition to 9 tomato handlers (3 tomato farmers, 3 tomato retailers and 3 tomato wholesalers) who participated in Focus Group Discussion (FGDs) per district. Collected data was entered into MS-Excel 13 and exported into STATA SE 14.0 for cleaning and analysis at 5% level of significance and 95% Confidence Intervals (CI). Proportion of risk perceptions and attitudes were computed and presented as percentages while factors associated with risk perception were determined using Fisher exact test. Qualitative data was analyzed using thematic content analysis.

Results: More than half, 54.2% (253/468), of the respondents were females, mean age was 37 years (SD=13.13, ranging from 18 to 88 years). Half of the respondents, 50.9% (238/467), were farmers by occupation and 40.3% (188/468) had completed upper primary education. Only 5.0% (20/396) of consumers reported a high risk perception towards tomatoes stained with pesticide residues, the rest, 95.0% (376/396), were buying pesticide stained tomatoes despite their awareness about the possible health effects. The main reason for buying the pesticide stained tomatoes was that a majority, 59.0% (230/390), lacked an alternative to stained tomatoes. However, consumers generally had a negative attitude towards pesticide stained tomatoes, with 67.0% (313/468) of the consumers disagreeing to a statement that tomatoes sold on the market are safe. Consumer risk perception was significantly associated with consumer awareness about residues in the tomatoes; where the proportion of consumers who were aware of the risk of pesticide stained tomatoes were

42.8 times more likely not to buy stained tomatoes compared to the proportion of those who were not aware. OR, 42.8 (95% CI: 10.76-170.28). However, after Fisher-Exact tests analysis, level of education P(0.975), gender P(0.581) and age-group P(0.680) were not associated with consumer risk perception (95% CI and 5% level of significance).

Conclusion: Consumer risk perception on pesticide stained tomatoes among Ugandan consumers ranked low with majority of the consumers buying tomatoes stained with pesticide residues due to lack of an alternative.

Key words: Consumer Risk Perception, Food Safety, Pesticide Residues.

Introduction:

Globally, there has been an increase in the inquiry on the knowledge of dangers of chemicals in food which has aroused consumer concerns on food safety(1, 2). This follows from consumer reports on health effects of pesticides from their inappropriate use, exposing consumers to high amounts of pesticide residues in harvested foods (3-5). Pesticide residues in food are directly related to the irrational application of pesticides on growing crops and to a lesser extent from residues remaining in the soil. Accumulated pesticide residues in food products are associated with human health hazards ranging from acute to chronic toxic effects (4, 6-8).

In the developing countries, almost all fruits and vegetables grown on a commercial scale are sprayed with pesticides to combat pests and diseases. A study done in 2014 at the two largest horticultural produce markets in Africa showed that 91% of the fruit and vegetable samples collected between 2012 and 2014 had pesticide residues although these were compliant with the Maximum Residue Limits (MRLs) (9).

For numerous decades, the use of pesticides has intensified globally in agriculture, homes and industries aiming at increasing productivity and reducing on the losses (4, 10-12)portant cost of production(13). In the Sub Saharan Africa with tropical climate that favors the growth and rapid multiplication of pests, pesticides are usually used at all levels of agricultural production including on farms to shield plants from pest attack and damage, to control weeds and parasites in livestock as well as in post-harvest control measures. It is now nearly impossible to produce food in tropical regions without using agro-chemicals, however, although this is considered to be among considerations have been made in the climate change mitigation strategies (14).

In most low-income countries like Uganda, pesticide regulation enforcement and support of the agricultural extension staff to guide farmers on pesticide application standards and dosage, is essentially lacking. Fresh produce sold at local markets is not usually ~~not~~ analyzed for agricultural chemical residues unlike export products. This raises concerns about the perceived safety levels of local food supplies in contrast to exported products (9). For instance, a study in Uganda showed that 24.5% of farmers were not aware of any health risks of spraying tomatoes close to harvest time, almost 50% of farmers (45.8%) sprayed their tomatoes less than a week ~~up~~ to harvest time, 29.2% sprayed their tomatoes on~~at~~ harvesting, with intentions to extend the shelf-life while 50% did so to attract consumers (15, 16).

Another study in 2015, shows how farmers sprayed tomatoes 6 times the manufacturer recommended dosage, and harvested these tomatoes 2-3 days after last spraying session compared to recommended pre-harvest interval of 4-7days (16). These phyto-sanitary practices increase pesticide residues in tomatoes, if there are ~~with~~ no control measures in place as ~~since~~ Uganda lacks a pesticide residue monitoring plan for conventionally grown food, ~~but also~~and given that the health sector has not prioritized prevention and control measures for pesticides poisoning (17).

Although developed countries use 75% of global pesticides; these countries apply them with strict regulations compared to developing countries which lack the regulation enforcement ~~or the regulations~~. Al~~T~~though developing countries use the least quantities of pesticides, they use the most toxic ones (18, 19) resulting in increased risks of acute poisoning. The inappropriate use of pesticides in developing countries increases pesticide exposure and health risks to the consumers. Approximately 25% of developing countries lack regulations and 50% of the WHO-region countries lack sufficient resources to enforce their pesticide-

related regulations (20-22). Also, under existing international laws, highly toxic, banned or unregulated pesticides are always exported to developing countries (23-26), posing health risks to consumers.

Although Uganda is in its transition of establishing a pesticide residue monitoring program, this haiis moveding on a slow pace and made protectioning of public health unrealisedmay take time to be realized. This study therefore aimed at understanding the attitudes and risk perception of consumers towards pesticide stained tomatoes, the associated, as well as their attitudes towards pesticide stained tomatoes to justify the need for with factors a plan of establishing evidence for the need of a pesticide residue monitoring program in for Uganda.

Methods

Study area and population

This study was conducted in the 4 districts as project interventional areas, each selected, from each of one of the four regions of Uganda namely; Northern (Nebbi), Western (Masindi), Eastern (Bugiri) and Central (Ssembabule). From each district, three sub counties were randomly selected and consumers sampledelected systematically at household level for interviews. The above districts were Pesticide Use, Health and Environment (PHE) Project intervention areas where with a tropical climate where tomatoes, cabbages, passion fruits, oranges, mangoes, okra, green peper, amarathus, eggplant among others, are commonly grown and intensively sprayed with pesticides and with agriculture as their first priority is a day to day business. The commonly used method of pesticide application is a knapsack sprayer which is worn as a backpack and mechanically operated as a hand pump.

The Uganda National census 2014 estimates average population for the above districts as follows; Nebbi (385,220), Masindi (94,622), Bugiri (426,000) and Ssembabule (219,600).

Study design

A cross-sectional study design conducted in June 2019 employed mixed methods of both qualitative and quantitative ~~methods of~~ data gathering. Working through already existing branches of the District Farmers Association (DFA) in each of the four districts, three sub counties were randomly sampled clustered into urban, peri-urban and rural. In each of the districts, at household level, at total of ~~on average~~ 468 ~~117~~ participants were randomly selected and interviewed from the fours districts as follows; 117 participants from Nebbi District, 102 participants from Sembabule district, 119 participants from Bugiri district and 130 participants from amasindi district~~making an overall total of 468 participants in the four districts~~. In addition, purposive sampling for Focus Group Discussions (FGDs) was done by the DFA focal person, forming a total of 9 participants per group per district. Each FGD was composed of three tomato farmers, three tomato retail vendors and three tomato wholesalers totaling to 36 participants for ~~in~~ the four districts.

Materials

Pretested and standardized structured questionnaires adopted from a survey "*A monitor on consumer confidence in food safety*" developed (by Janneke de Jorge, 2008) for monitoring consumer safety in a Canadian population were modified and used for data collection (details provided in the S5 File). All questionnaires were translated into the participant local

languages of participants and translated back into English for quality assurance purposes. Focus Group Discussion Guides were used to collect the qualitative data and also administered in the local language by trained Research Assistants (RAs).(details of the guides are provided in S6 File).

Consumer risk perception was assessed using a series of three questions in the order; 1) Are pesticide residues harmful to human health? 2) Are you aware that tomatoes sold on local markets contain pesticide residues? 3) Do you buy pesticide stained tomatoes? Attitudes were measured on a three-Likert scale (responses ranging from agree, not sure and disagree). with questionnaire included questions three sections on optimism, pessimism and trust. On the side ofunder optimism section, questions assessed the safety, confidence and satisfaction about the pesticide residues on tomatoes. Section on while pessimism assessed the worrisomeness, suspicion and discomfort caused by the pesticides residues on the tomatoes, while section on trust assessed the consumer's trust in whether the tomato vendors had the characters of trust such as the competence to control safety of tomatoes, the knowledge to guarantee tomato safety, honesty about the safety of the tomatoes, sufficiently open about tomato safety and giving special attention to control the safety of tomatoes (Assessment results provided in S2 File).

Data collection and analysis

Research Assistants (RAs) were trained on the objective of the study in a one day training per district, questionnaire were pretested with the RAs and supervision done on a daily basis, every filled-in questionnaire was reviewed to ensure that they were fully filled in and that ethical considerations were not bleached.

Trained RAs from the respective District Farmer Associations were enumerated to gather quantitative data in each of the 3 sub counties per district, and one RA per district assisted in but also where necessary used as translatorsdoing translations during FGDs for qualitative data collection. A total of 36 respondents were involved in the FGD, 9 per district, all their responses recorded on audio and data gathered on tapes. Sample size for the FGDs was based on the level of saturation of the responses.

Quantitative Collected data was gathered and entered into Microsoft Excel Version 2013 and exported into Stata SE.14 for cleaning and analysis. A total of 468 entries were achieved. Categorical variables like Risk Perception (measured as a binary outcome, high risk or low risk perception) and Attitude, age group, occupation, gender, and level of education were presented as frequencies with their respective percentages, while continuous variable such as age presented as means with their respective (SD) and ranges.

Bivariable analysis for risk perception (measured as a binary outcome), was computed by gender, level of education, age categories, residence (rural, urban and peri-urban) and the chi-square plus the respective p-values reported under (95% CI, 5% Level of significance) reported. Awareness about the pesticide residues was computed by level of education and by the practice of buying tomatoes and their Chi-square, p-values under (95% CI and 5% Level of significance) reported. Fisher exact test was used to determine the factors associated with consumer risk perception and the factors for buying of pesticide stained tomatoes under (95% CI, 5% Level of significance), details provided in the S3 File. Finally, simple logistic regression was used to compute the odds of high risk perception by consumers who were aware of pesticide residues on the tomatoes and their respective Odds Ratio with the p-values reported under with (95% CI and 5% Level of significance.

Qualitative data collected among the 36 participants in the four districts was transcribed and analysis done thematically based on the study objectives ~~using deductive approach of reasoning, making~~ and conclusions based on participant responses. These conclusions were later used to support a discussion with quantitative data findings.

Ethical consideration

This study sought ethical approval from Makerere University School of Public Health, Higher Degree Research and Ethics Committee (MakSPH HDREC) with an approval reference registration number 686. Informed consent was sought from all participants before the interviews, for anonymity, participants initials were used instead of their names on the questionnaire and ~~they~~ participants were free to withdraw from the study at any point when they felt like not continuing with the interviews.

Results

Demographic characteristics of consumers

The study registered 100%all response from consumers, equally sampled by residence (rural, urban and peri-urban)., interviewed from the three sub counties in each of the four districts of Uganda; (Northern region: Nebbi district, Eastern Region: Bugiri District, Central Region: Sembabule district and Western region: Masindi district).

As indicated in Table 1, Slightly more than half, 54.1% (253/468), of respondents were females, a majority, ≈51.0% (238/468) practiced farming as an occupation (*refer to S1* File), a majority, 84.4% (273/468), had attained lower level of education. The mean age of participants was 37.7years (SD±13.1, ranging from 18-88) with majority and 54.7% (256/468) belonging to were falling into the age group below the mean age. From the qualitative results, interviews involved categories of tomato farmers 33.3% (12/36), tomato retail vendors 33.3% (12/36), and tomato wholesalers 33.3% (12/36) sampled in equal proportions in the 4 districts. i.e. three persons per category per district.

Table 1: Demographic characteristics of consumers

Variable	Category	Frequency (%)
Gender<br>(n=468)	Male<br>Female	214 (45.8)<br>253 (54.2)
Age group	Below mean Age<br>Above mean Age	256 (54.7)<br>212 (45.3)
	**Mean Age**	37.7 (SD±13.1)

Level of education<br><br>(n=468)	No formal education<br><br>Lower level<br><br>upper level	39 (8.3)<br><br>395 (84.4)<br><br>34 (7.3)

General consumer attitudes towards pesticide stained tomatoes

Consumer negative attitude (pessimism) towards pesticide stained tomatoes.

Basing on the ~~computed~~ percentages of pessimism, measured on a 3-Likert scale, a majority, 74.3%(347/467) of the consumers had a negative attitude (on the agreement side) towards the stains on the tomatoes compared to 2.4%(11/467) who were not sure and 23.5% (109.6/467) who felt positive about the stains on the tomatoes in terms of worrisomeness, discomfort and suspicion caused by ~~that~~ the residues. ~~cause~~ (details provided in S2 File and Fig1).

Fig 1. Average negative perceptions of consumers on pesticide stained tomatoes in percentage.

Consumer positive attitude (optimism) towards pesticide stained tomatoes.

Basing on the ~~computed~~ percentages of optimism, measured on a 3-Likert scale, Consumer positive attitude towards the pesticide stains was low. On average, only 33.2% (155.3/468) of the consumers agreed to the statement that tomatoes sold on the Ugandan market are safe,

compared to a majority 61.1%(285.6/468) who disagreed with the statement while 5.7% (26.7/468) were not sure as provided in Fig2. and indicated S2 File.

Fig 2: Average positive perceptions of consumers on pesticide stained tomatoes in percentages.

Consumer level of trust in pesticide stained tomatoes

In terms of trust, based on the 3-scale resized ~~computed~~ averages for trust, about 77.7 % (362/466) of consumers lacked trust and disagreed that tomato vendors have the characters of trust such as the competence to control safety of tomatoes, the knowledge to guarantee tomato safety, honesty about the safety of the tomatoes, sufficiently open about tomato safety and gave~~iving~~ special attention to control the safety of tomatoes compared to 8.1% (37.6/466) who were not sure and 14.1%(65.4/466) who trusted tomato vendors (details provided in the S2 File).

Consumer risk perception level towards pesticide stained tomatoes

Consumer risk perception was measured using a model with questions as provided in Fig 3.

Fig 3. Model for determining consumer risk perception

Consumer risk perception

Among consumers who were aware and knowledgeable about sold tomatoes containing pesticide residues, ≈95.0% (376/396) of them bought these pesticide stained tomatoes (i.e. had a low risk perception) compared to only 5.0% (20/396) who perceived tomatoes to be of high risk to their health and withdrew from buying them (i.e. had a high risk perception) as provided in Fig 4.

Fig 4. level of consumer risk perception towards pesticide stained tomatoes

Reasons for buying pesticide stained tomatoes.

The main reasons for buying pesticide stained tomatoes was that, 59.0% (230/390) of the consumers had no alternative choice to buying pesticide stained tomatoes, followed by 27.2% (106/390) who had to prepare these tomatoes to reduce on the residues, 9.2% (36/390) who perceived no health risks of buying stained tomatoes and 4.6% (18/390) falling in the other categories which included the preference of tomato attributes ~~of the tomatoes~~ like size, ripeness among other than the pesticide residues.

Consumer confidence on the safety of tomatoes sold on Ugandan Market.

On a general scale, more than half, 66.9% (313/468), of the consumers disagreed to the fact that tomatoes sold on the Ugandan markets are safe, consumers' general confidence on the safety of tomatoes sold on Ugandan Markets outweighed their counterparts with nearly half, 49.6%(231/466), of the consumers being confident about the safety of tomatoes sold on the Ugandan markets while only 14.4%(168/466) were not confident and 14.4% (167/466) were not sure.

Factors associated with consumer risk perception and buying of stained tomatoes.

From the Fisher-exact tests, Consumer risk perception was not associated with the level of education P(0.975), Residence P(0.462), gender P(0.581), age-group P(0.680) and marital status P(0.581), as indicated in the S3 File. Further simple logistic regression analysis revealed that consumer risk perception was significantly associated with consumer awareness about residues in the sold tomatoes; where the proportion of consumers who were aware that tomatoes contain pesticide residues being 42.8 fold more of a high risk perception compared to the proportion of those who were not aware of the residues as provided in the S4 File.

In table 2, the consumer awareness about Pesticide residues was not associated with age-group and level of education but significantly associated with gender, where male consumers were 1.77 time more likely to be aware of the pesticide residues in the tomatoes compared to counter parts; the second factor was whether consumers ever obtained pesticide safety information where consumers who had never obtained pesticide safety information being 61% less likely to be aware of the pesticide residues compared to consumers who had obtained information on pesticide safety, OR 0.39 (95% CI: 0.24-0.64)

Table 2: Logistic Regression for Consumer awareness about pesticide residues on tomatoes and some consumer demographics (crude Odds ratio)

	**Aware of pesticide residues No (%) Yes (%)**	**Odds Ratio**	**Std. Err.**	**P>|z|**	**[95% CI]**	
**Gender**						
Female	54(21.3) 200(78.7)	1.0				

Male	28(13.2) 184(86.8)	1.77	.451	**0.024**	1.078	2.921
**Age category**						
Below mean age	46(18.0) 210(82.0)	1.0				
above mean age	36(17.1) 174(82.9)	1.06	.259	0.816	.655	1.711
**Education category**						
None	10(25.6) 29(74.4)	1.0				
Lower level Education	69(17.6) 324(82.4)	1.62	.631	0.217	.754	3.477
Upper level Education	3(8.8) 31(91.2)	3.56	2.52	0.072	.891	14.249
**Ever obtained information about pesticide safety**						
Yes	34(12.1) 247(87.9)	1.0				
No	48(25.9) 137(74.1)	0.39	.098	**0.000**	.244	.639

Consumer awareness about the pesticide stained tomatoes was not associated with consumer level of education P(>0.05) but significantly associated with consumer risk perception P(<0.05) and the practice of buying stained tomatoes P(<0.05) as indicated in table 3 below.

Table 3: showing the fisher exact tests for ~~associations between~~ awareness about pesticide residues in tomatoes verses~~with~~ the consumer level of education and practice of buying stained tomatoes.

**Buy stained tomatoes**	**Aware of pesticide residues in the tomatoes Freq (%)**			**Fishers-exact p-values**
	**Yes**	**No**	Not sure	**0.000**
No	14/20(70.0)	6/20(30.0)	0 /20(0.0)	

Yes	370/376(98.4)	4/376(1.1)	2/376(0.5)	
**Consumer risk perception**				**0.000**
High risk perception	13/19(68.4)	6/19(31.6)	0/19(0.0)	
Low risk perception	371/377(98.4)	4/377(1.1)	2/377(0.5)	
**Education level**				0.095
None	29/39(74.4)	7/39(17.9)	3/39(7.7)	
primary	216/272(79.4)	35/272(12.9)	21/272(7.7)	
secondary	108/121(89.3)	7/121(5.8)	6/121(5.0)	
tertiary	31/34(91.2)	1/34(2.9)	2/34(5.9)	

Qualitative findings

Theme	Main claim	Underlying issue	Preposition	Argument/disagreement
**Pesticide effects on human health**	From our qualitative findings, most participants, 41.2% (7/17) claimed that pesticide stained tomatoes are poisonous, harmful to human health and create fear for the consumer's health. *"To me it is real poison because even on the pesticide label the manufacturer cautions the users to put on protective gears. This puts me at risk as a farmer and puts fears to the final consumer that is not safe", one of the Farmers from Masindi district said. These residues are harmfull to our health because from the time of planting to harvesting a farmer uses over 10 chemical types in order to bring out good results meaning that these chemicals get dissolved inside the tomato juice", one of the wholesaler from Sembabule district said.*	Some participants claimed that spraying tomatoes with pesticides was fine as long as they are washed before eating. However, some claimed that spraying high dosages leads to more residues/stains on tomatoes which may affect the consumers. Some participants were of the opinion that residues indicate high doses of pesticide spraying before sale to the market rendering tomatoes unsafe for consumption.	Participants mainly suggested that tomatoes need to be washed before sale in the market and consumption to ensure safety, while other participants argued that tomatoes can be soaked in water for one (1) hour before consumption or the outer cover peeled off in order to reduce exposure in consumed food.	However, there was a diversion in perception about the harmfulness of pesticides, Some Participants argued that Pesticides are only harmful to pests on plants and not humans, they protect tomatoes from damage, keep them safe and ready for consumption, *"Since the pesticides are used to kill pests, the chemical is only harmful to the pests affecting tomatoes."* narrated by a farmer from Olyeko village, Nebbi municipality.<br><br>Some participants were more interested in the appearance of pesticide residues/stains on tomatoes claiming that it assured them of the market since customers preferred such tomatoes. Participants discussed that the residues are both on top and inside, preserve the tomatoes and reduce losses and customers had to wash the tomatoes clean of pesticides. Participants also

					mentioned that they spray because of environmental related issues claiming that the environment can no longer support proper tomato growth without use of pesticides and Pesticides are used to increase yields. Unfortunately, participants were aware that farmers don't follow instructions on labels and rarely use any protective equipment as these are not easily accessible and affordable.

**Perception of residue and impact on consumer health**	Most of the Participants 47.1% (8/17) said that pesticides negatively affect consumer health. This is mainly because most of them had experienced a sign of pesticide poisoning, for instance some claimed itching, irritations, stomachaches, and restlessness. Some participants were aware of the effects from the pesticide labels while some claimed that the smell of pesticides indicates that they have a negative effect on health.<br><br>Most participants 70.6% (12/17) claimed pesticides were harmful due to the health problems they cause whereas some, 29.4% (5/17) were somehow optimistic and argued that pesticide residues are not very harmful because the effects are observed after a very long time.	Some participants proposed that Consumers need to be sensitized on pesticide dangers, including the effects of the residues. Some participants argued that due to the low market prices and limited resources, farmers tend to delay harvesting tomatoes by spraying them to keep them longer until there is a better market price.	Despite the impact, some participants argued that Farmers overdose tomatoes with pesticides to meet of customers' demand of storing tomatoes for a longer time. Customers in market shun pesticides without pesticide residues; they demand those with residues because they believe they are healthy and last longer. Some attributed this practice to the tomato varieties on market which require a lot of spraying throughout the growth stages to maximize yields. Participants argued that farmers can't interpret the labels on pesticide containers because most of them are not educated and therefore may overdose or under dose the tomatoes with pesticides. In addition, some participants claimed that pesticides are not very bad if used properly but farmers don't observe pre-harvest interval.<br><br>Some participants recommended that the government plays its role to obliterate some pesticides including counterfeits from the market, while others recognized that farmers also have a role to play by following the prescription made by the manufacturer from the labels.	
**Considerations before**	Some participants claimed they don't pay attention to pesticide residues and buy	Participants recommend that		Some participants claimed that tomatoes are

**buying tomatoes**	tomatoes with pesticides because they last longer. Some participants consider tomato size (prefer big to small), tomato ripeness (prefer not so ripe tomatoes), freshness and customer needs.<br>*"We look for general appearance of tomato but at sight I look at the pesticide residue on tomato not the size. I would better go with small tomato with pesticide than a big one."* answered a farmer from Masindi district.<br>However, a few wholesalers consider Place of tomato selling. They say that usually tomatoes from non-mulched gardens spoil faster especially during rainy seasons. So they would like to make loses by discarding most of the damaged tomatoes. *"Sure deal, traders consider the pesticide residues on the tomato, and the quality of the tomatoes is determined by the life expectancy of that tomato. A good tomato turns red not yellow in appearance and doesn't have disease spots when ready. Traders consider the glittering cover, and size. For tomatoes sprayed from the store, the residue is just on top and can easily be rubbed off with your mere hands but a tomato which has been sprayed earlier can't rub off the residues even if they are being seen."* explained a tomato whole seller from Masindi district.<br>*"When the buyers are many in his garden they don't*	Researchers share information with local people to understand the health impact of pesticides and how to reduce exposure.		sprayed shortly before harvesting to stores, because some buyers may book the tomatoes and fail to pick up on the exact promised day.<br>All Participants however, claimed no further spraying is done at the stalls because they are already sprayed well and need to preserve quality and harden skin.

		consider or mind anything they just collect all except the damaged ones" said a farmer from Nebbi district.			

~~Pesticide effects on human health~~

~~Main claim~~

~~From our qualitative findings, most participants, 41.2% (7/17) claimed that pesticide stained tomatoes are poisonous, harmful to human health and create fear for the consumer's health.~~

~~*"To me it is real poison because even on the pesticide label the manufacturer cautions the users to put on protective gears. This puts me at risk as a farmer and puts fears to the final consumer that is not safe", one of the Farmers from Masindi district said.*~~

~~*These residues are harmfull to our health because from the time of planting to harvesting a farmer uses over 10 chemical types in order to bring out good results meaning that these chemicals get dissolved inside the tomato juice", one the wholesaler from Sembabule district said.*~~

~~Underlying Issues~~

~~Some participants claimed that spraying tomatoes with pesticides was fine as long as they are washed before eating. However, some claimed that spraying high dosages leads to more residues/stains on tomatoes which may affect the consumers.~~

~~Some participants were of the opinion that residues indicate high doses of pesticide spraying before sale to the market rendering tomatoes unsafe for consumption.~~

Proposition:

Participants mainly suggested that tomatoes need to be washed before sale in the market and consumption to ensure safety, while other participants argued that tomatoes can be soaked in water for one (1) hour before consumption or the outer cover peeled off in order to reduce exposure in consumed food.

Argument /disagreement:

However, there was a diversion in perception about the harmfulness of pesticides, Some Participants argued that Pesticides are only harmful to pests on plants and not humans, they protect tomatoes from damage, keep them safe and ready for consumption, "*Since the pesticides are used to kill pests, the chemical is only harmful to the pests affecting tomatoes*." narrated by a farmer from Olyeko village, Nebbi municipality.

Some participants were more interested in the appearance of pesticide residues/stains on tomatoes claiming it assured them of the market since customers preferred such tomatoes. Participants discussed that the residues are both on top and inside, preserve the tomatoes and reduce losses and customers had to wash the tomatoes clean of pesticides.

Participants also mentioned that they spray because of environmental related issues claiming that the environment can no longer support proper tomato growth without use of pesticides and Pesticides are used to increase yields.

Unfortunately, participants were aware that farmers don't follow instructions on labels and rarely use any protective equipment as these are not easily accessible and affordable.

Perception on impact of stains/pesticide residues on consumer health

Underlying issues

Main claim

Most of the Participants 47.1% (8/17) said that pesticides negatively affect consumer health. This is mainly because most of them had experienced a sign of pesticide poisoning, for instance some claimed itching, irritations, stomachaches, and restlessness. Some participants were aware of the effects from the pesticide labels while some claimed that the smell of pesticides indicates that they have a negative effect on health.

Proposition

Some participants proposed that Consumers need to be sensitized on pesticide dangers, including the effects of the residues.

Argument

Despite the impact, some participants argued that Farmers overdose tomatoes with pesticides to meet of customers' demand of storing tomatoes for a longer time. Customers in market shun pesticides without pesticide residues; they demand those with residues because they believe they are healthy and last longer.

Some attributed this practice to the tomato varieties on market which require a lot of spraying throughout the growth stages to maximize yields.

Participants argued that farmers can't interpret the labels on pesticide containers because most of them are not educated and therefore may overdose or underdose the tomatoes with pesticides.

Formatted: Centered

In addition, some participants claimed that pesticides are not very bad if used properly but farmers don't observe pre-harvest interval.

Perceived danger of stains

Main claim

Most participants 70.6% (12/17) claimed pesticides were harmful due to the health problems they cause whereas some, 29.4% (5/17) were somehow optimistic and argued that pesticide residues are not very harmful because the effects are observed after a very long time.

Underlying issues

Some participants argued that due to the low market prices and limited resources, farmers tend to delay harvesting tomatoes by spraying them to keep them longer until there is a better market price.

Proposition

Some participants recommended that the government plays its role to obliterate some pesticides including counterfeits from the market, while others recognized that farmers also have a role to play by following the prescription made by the manufacturer from the labels.

Perception on considerations before buying tomatoes

Main claim

Some participants claimed they don't consider pesticide residues and buy tomatoes with pesticides because they last longer. Some participants consider tomato size (prefer big to small), tomato ripeness (prefer not so ripe tomatoes), freshness and customer needs.

"We look for general appearance of tomato but at sight I look at the pesticide residue on tomato not the size. I would better go with small tomato with pesticide than a big one." answered a farmer from Masindi district.

However, a few wholesalers consider Place of tomato selling. They say that usually tomatoes from non-mulched gardens spoil faster especially during rainy seasons. So they would like to make loses by discarding most of the damaged tomatoes. *"Sure deal, traders consider the pesticide residues on the tomato, and the quality of the tomatoes is determined by the life expectancy of that tomato. A good tomato turns red not yellow in appearance and doesn't have disease spots when ready. Traders consider the glittering cover, and size. For tomatoes sprayed from the store, the residue is just on top and can easily be rubbed off with your mere hands but a tomato which has been sprayed earlier can't rub off the residues even if they are being seen."* explained a tomato whole seller from Masindi district.

"When the buyers are many in his garden they don't consider or mind anything they just collect all except the damaged ones" said a farmer from Nebbi district.

Proposition

Participants recommend that Researchers share information with local people to understand the health impact of pesticides and how to reduce exposure.

Argument

Some participants claimed that tomatoes are sprayed shortly before harvesting to stores, because some buyers may book the tomatoes and fail to pick up on the exact promised day. All Participants however, claimed no further spraying is done at the stalls because they are already sprayed well and need to preserve quality and harden skin.

Discussion

Very little information is available about the pesticide residue in tomatoes in Uganda and consequently the risk of exposures to this. The focus of this study was~~is~~ to determine the consumer risk perception towards pesticide stained tomatoes and pesticide residues, and the attitudes (pessimism vs optimism) towards the safety of the pesticide stained tomatoes. It employed a cross-sectional study design with a total of 468 consumers as respondents equally sampled by residence and interviewed from each of the four districts (Northern region: Nebbi district, Eastern Region: Bugiri District, Central Region: Sembabule district and Western region: Masindi district), thus a good representation of Ugandan consumers.

Characteristics of the respondents show that slightly more than half of respondents were females as expected since the majority of these stay home to take care of home chores, similar to findings by (27) where 58% of women were involved in the purchase of meat, more than three quarters were married women as interviews were conducted at homesteads and consent sought from adults making it more likely for the married women to be interviewed. Half of the respondents were farmers given that Uganda as a country has ~~more than~~nearly three quarters of its population engaged in farming, and also~~given that~~ this study was carried out in a rural setting, involving vendors, buyers and tomato growers. A majority had completed lower level of Education and a majority belonged to age group <30 years as the Ugandan population is composed of 75% youths as the majority(28), 80% of these residing in rural areas(29). From these characteristics we can definitely report that the consumers interviewed in this study were a good representation of tomato consumers in Uganda(28). Respondents of this study represent majority adult female consumers experienced with tomato farming and with adequate level of education (majority with

Ordinary level of education) to well express well their risk perception towards pesticide stained tomatoes.

On a general note, cConsumer risk perception ranked low with a majority of consumers (95%) buying tomatoes what is well known to be stained with pesticide residues, a majority giving reasons that they have no alternative. Results from this study highly deviate from similar studies conducted in developed countries such as among Californian consumers where 80% were safety cautious and checked the food items to see if they were opened or damaged (30). While in Georgia, 89% considered testing of pesticide residues in food to be very important or somewhat important(31) and in Boston, consumers had a high risk perception of conventionally grown produce compared to public health hazards(32), most of these were mainly triggered by the health effects of the contaminants such as pesticide residues and biological contaminants in the sold produce.

From the Turkish perspective, consumers' willingness to pay for reduced pesticide residues in tomatoes was mainly determined by their risk perception about the residues which is explained by the label on the purchased apples(33). In this case due to the different situations, in Uganda unfortunately tomatoes are not labeled with the residual contents and benefits of low pesticide residual levels. This could be the reason why a majority of the consumers in this study bought the tomatoes. The markets vegetables on the market due to lack of such information which could have triggered consumersthem to make their choices. This is a gap to be closed by authorities in charge of food safety. Uganda lacking a stad alone and comprehensive food safety policy puts public health at stake for the pesticide exposures. Results from this study are a vivid evidence to be used as part of the advocacy statements in finding ways of establishing a a stand a lone National Food Safety Policy.

A study on consumers’ willingness to pay for pesticide-free vegetables indicated how consumer awareness about the residues in vegetables and the residual effects on human health greatly influenced their willingness to pay for these vegetables. Consumers in this study were willing to pay 50% more for the pesticide-free vegetables (34). In the Ugandan context, the consumer's low risk perception on pesticide-stained tomatoes indicates a risk of increasing exposures to pesticide residues if these lack an alternative. As reported in other findings (35), our qualitative results from~~by~~ vendors and tomato farmers from the FGDs, indicate that the highly stained tomatoes are due to poor hybrid tomato seeds that need frequent spraying.~~;~~ ~~v~~Vendors' demand from tomato growers to spray tomatoes before ~~they~~ sell. But ~~-~~also a low level of literacy to understand the pesticide label information coupled with a wrong perception of tomato farmers that, Mancozeb pesticide can harden the outer skin and increase tomato shelf life. It is from this misconception that tomato vendors only buy stained tomatoes presuming that these tomatoes will stay long on shelf, are healthy & free from the microbial contaminants.

Unlike other studies(31) from Georgia, where consumers prioritize microbial contamination followed by pesticide residues as the first consideration before making a choice to buy vegetables, tomato consumers in Uganda partly have a feeling that pesticide stained tomatoes are free from pathogens and thus healthy, giving not much priority to pesticide residues. As reported some think that pesticides are selective and only meant to kill plant pests and cure plant diseases. However, our logistic regression analysis indicates how consumer awareness about pesticide residues increases chances of a high risk perception, protecting consumers from residue exposure, ~~but~~ although this is just ~~to~~ for a few individuals.

From our focus group discussion of vendors and farmers, all farmers claimed that vendors would only buy tomatoes with pesticide residues as these are thought healthy. On the other

hand, vendors attest that stained tomatoes are healthy, look good, take long to go stale and have a high resale value on the market.

From these findings, Ministry of Agriculture Animal Industry and Fisheries needs to sensitize farmers as well as improve coordination and regulations on the sale and use of agro-inputs. Agro-input dealers who are the immediate information providers to the farmers, need to be trained in Pesticide safe-use training. A recent unpublished survey done by UNACOH in 2020, reports that only 6% of the agro-input dealers in 12 districts to have obtained the safe-use training, a training required to be undertaken by all agro-input distributors before ~~starting~~ opening up their business.

Tomato residues are given less attention by the consumers probably because consumers lack knowledge of the dangers that the residues may impact on their health which in most cases takes time. From a model by Huang Chung (36) estimating the relationship between consumer perceptions, attitudes and behavioral intentions (S7 File), choices (behavioral intentions) on buying food is influenced by perceptions and attitudes, which influence each other in addition to knowledge (information) from personal experience, evaluative criteria and social demographics. All based on the consumer awareness about the pesticide's potential ill effects but also on a larger extent the social economic status of the consumer. Consumers of a higher social economic status are most likely to have a high educational level and consequently easily access all the necessary information about the effects of pesticide residues. These will tend to have a high risk perception about the pesticide stained tomatoes and not likely to buy tomatoes which are stained with pesticides. However, this was not so with our finding. Consumer risk perception was not directly associated with level of education although associated with awareness about pesticide residues, where consumers

who were aware of pesticide residues were 42.8 times more likely to be of high risk perception compared to those who were not aware. From our results the low proportions (5%) of high risk perception consumers may be largely attributed to our sample containing low percentages of highly educated consumers (upper level education, table 1).

On the other hand, pesticide residue knowledge among the general public in Uganda is a new topic and studies conducted along these lines are few, most of the time not intended for creating awareness among the public on the potential ill effects of residues in food. From other related findings in this study, consumers have no access to sources of information on pesticide residues in food with most of the information on pesticide residues acquired through radio and television media, followed by health professionals, depicting a big gap in information accessibility, but also its availability.

Conclusion

Consumer risk perception among Ugandan consumers was ranked low with a majority of tomato consumers buying tomatoes stained with pesticides regardless of their level of education, age and gender, this all linked to lack of alternative organic tomatoes on the market. However awareness that tomatoes contain pesticide residues was associated with consumer risk perception where the proportion of consumers who were aware of tomatoes containing pesticide residues was 42.8 fold more of high risk perception than consumers who were not aware.

There is a need by the government through its line of Ministry of Agriculture Animal Industry and Fisheries (MAAIF) and other health information dissemination Civil Society Organization to sensitize the Ugandan population on the effects of pesticide residues, the

farmers on the dosage and the pre-harvest intervals as well as train Agro-input Dealers from which farmers buy pesticides.

MAAIF should also hasten the establishment of the National Pesticide Residue Monitoring Program to protect Public Health from this chronic exposure to pesticide residues in agricultural produce.

Acknowledgments

The author would like to acknowledge the support from Diálogos participants and all efforts rendered by the Pesticides use, Health and Environment (PHE) Project team, and the respective District Framers' Association with which the study was conducted. DFAs supported highly in questionnaire translation into the respective local languages and in data collection.

Supportive information

Data-set used to yield the above results is available upon request from the author. However, separate supportive information has been attached as highlighted below.

S1 File. Other Demographic characteristics of consumers. PDF

S2 File. Consumer attitudes towards pesticide stained tomatoes. PDF

S3 File. Fisher-exact tests for the factors associated with consumer risk perception. PDF

S4 File. Simple logistic regression of consumer risk perception vs awareness about pesticide residues. PDF

S5 File. Questionnaire: Consumers risk perception towards pesticide stained tomatoes in Uganda. PDF

S6 File. Focus Group Discussion guide for Consumers risk perception towards Pesticides stained tomatoes in Uganda. PDF

S7 File. A model for estimating the relationship between consumer perceptions, attitudes and behavioral intentions. PDF

Reference

1. Winter CK. Pesticide residues in imported, organic, and "suspect" fruits and vegetables. Journal of agricultural and food chemistry. 2012;60(18):4425-9.
2. Winter CK, Katz JM. Dietary exposure to pesticide residues from commodities alleged to contain the highest contamination levels. Journal of toxicology. 2011;2011.
3. Codex Pesticides Residues in Food Online Database [Internet]. © FAO/WHO, 2019. 2016 [cited 22/02/2019]. Available from: http://www.fao.org/fao-who-codexalimentarius/codex-texts/dbs/pestres/en/.
4. WHO. Pesticide residues in food 2018 [16/04/2019]. Available from: https://www.who.int/en/news-room/fact-sheets/detail/pesticide-residues-in-food.
5. Hamilton D, Ambrus Á, Dieterle R, Felsot A, Harris C, Petersen B, et al. Pesticide residues in food—acute dietary exposure. Pest Management Science: formerly Pesticide Science. 2004;60(4):311-39.

6. Kim K-H, Kabir E, Jahan SA. Exposure to pesticides and the associated human health effects. Science of the Total Environment. 2017;575:525-35.
7. Jones JL, Hanson DL, Dworkin MS, Alderton DL, Fleming PL, Kaplan JE, et al. Surveillance for AIDS-defining opportunistic illnesses, 1992-1997. MMWR CDC Surveill Summ. 1999;48(2):1-22.
8. Hansen MRH, Jørs E, Sandbæk A, Sekabojja D, Ssempebwa JC, Mubeezi R, et al. Exposure to cholinesterase inhibiting insecticides and blood glucose level in a population of Ugandan smallholder farmers. Occupational and Environmental Medicine. 2020.
9. Mutengwe MT, Chidamba L, Korsten L. Monitoring pesticide residues in fruits and vegetables at two of the biggest fresh produce markets in Africa. Journal of food protection. 2016;79(11):1938-45.
10. Wilson C, Tisdell C. Why farmers continue to use pesticides despite environmental, health and sustainability costs. Ecological economics. 2001;39(3):449-62.
11. Zhang W, Jiang F, Ou J. Global pesticide consumption and pollution: with China as a focus. Proceedings of the International Academy of Ecology and Environmental Sciences. 2011;1(2):125.
12. WHO Suicide Fact Sheet [Internet]. 2018 [cited 11/08/2018]. Available from: http://www.who.int/en/news-room/fact-sheets/detail/suicide.
13. Cooper J, Dobson H. The benefits of pesticides to mankind and the environment. Crop Protection. 2007;26(9):1337-48.
14. Majaliwa J, Mukwaya P, Isubikalu P, editors. Climate change adaptation strategies in the semi-arid region of Uganda. 2 nd Ruforum Biennial meeting; 2010.
15. Atuhaire A, Ocan D, Jørs E. Knowledge, Attitudes, and Pratices of Tomato Producers and Vendors in Uganda. Advances in Nutrition and Food Science. 2016;1(1):1-7.
16. Kaye E, Nyombi A, Mutambuze IL, Muwesa R. Mancozeb residue on tomatoes in Central Uganda. Journal of Health Pollution. 2015;5(8):1-6.
17. Sekabojja D, Atuhaire A, Nabankema V, Sekimpi D, Bainomugisa C, Jørs E. Acute Pesticide Poisoning Case Registration in Uganda's Health Care Facilities. Journal of Environmental & Analytical Toxicology. 2020;10(2).
18. Oesterlund AH, Thomsen JF, Sekimpi DK, Maziina J, Racheal A, Jørs E. Pesticide knowledge, practice and attitude and how it affects the health of small-scale farmers in Uganda: a cross-sectional study. African health sciences. 2014;14(2):420-33.
19. Binukumar B, Bal A, Sunkaria A, Gill KD. Mitochondrial energy metabolism impairment and liver dysfunction following chronic exposure to dichlorvos. Toxicology. 2010;270(2-3):77-84.
20. Matthews G, Zaim M, Yadav RS, Soares A, Hii J, Ameneshewa B, et al. Status of legislation and regulatory control of public health pesticides in countries endemic with or at risk of major vector-borne diseases. Environmental health perspectives. 2011;119(11):1517-22.
21. Handford CE, Elliott CT, Campbell K. A review of the global pesticide legislation and the scale of challenge in reaching the global harmonization of food safety standards. Integrated environmental assessment and management. 2015;11(4):525-36.
22. Ogada DL. The power of poison: pesticide poisoning of Africa's wildlife. Annals of the New York Academy of Sciences. 2014;1322:1-20.
23. Phung DT, Connell D, Miller G, Rutherford S, Chu C. Pesticide regulations and farm worker safety: the need to improve pesticide regulations in Viet Nam. Bulletin of the World Health Organization. 2012;90:468-73.
24. Dinham B. Growing vegetables in developing countries for local urban populations and export markets: problems confronting small-scale producers. Pest management science. 2003;59(5):575-82.

25. Ecobichon DJ. Pesticide use in developing countries. Toxicology. 2001;160(1-3):27-33.
26. Smith C, Root D. The export of pesticides: shipments from U.S. ports, 1995-1996. International journal of occupational and environmental health. 1999;5(2):141-50.
27. Spais GS, Vasileiou KZ. An ordinal regression analysis for the explanation of consumer overall satisfaction in the food-marketing context: The managerial implications to consumer strategy management at a store level. Journal of Database Marketing & Customer Strategy Management. 2006;14(1):51-73.
28. UNFPA. UGANDA'S YOUTHFUL POPULATION. 2020.
29. USAID. COMMUNITY HEALTH SYSTEMS CATALOG
COUNTRY PROFILE: UGANDA 2017.
30. Bruhn CM, Schutz HG. Consumer food safety knowledge and practices. Journal of food safety. 1999;19(1):73-87.
31. Misra SK, Huang CL, Ott SL. Consumer willingness to pay for pesticide-free fresh produce. Western Journal of Agricultural Economics. 1991:218-27.
32. Williams PR, Hammitt JK. Perceived risks of conventional and organic produce: pesticides, pathogens, and natural toxins. Risk analysis : an official publication of the Society for Risk Analysis. 2001;21(2):319-30.
33. Akgungor S, Miran B, Abay C. Consumer willingness to pay for reduced pesticide residues in tomatoes: the Turkish case. 1999.
34. Coulibaly O, Nouhoheflin T, Aitchedji C, Cherry A, Adegbola P. Consumers' perceptions and willingness to pay for organically grown vegetables. International journal of vegetable science. 2011;17(4):349-62.
35. Kaaya N. DITHANEM-45 RESIDUES IN TOMATOES ON UGANDAN MARKETS MAY BE ABOVE SAFE LEVELS. African Journal of Food, Agriculture, Nutrition and Development. 2004;4(1).
36. Huang CL. Simultaneous‐equation model for estimating consumer risk perceptions, attitudes, and willingness‐to‐pay for residue‐free produce. Journal of Consumer Affairs. 1993;27(2):377-96.

Dear Editor:

I have reviewed an article titled "Consumer risk perception towards pesticide stained tomatoes in Uganda" The article data depict some interesting information; however, the scientific writing and presentation of data need to improve. This article needs substantial revision with proper sequence, I would advise to the authors please, read and follow some good articles to present your data.

Introduction

1. The introduction needs to be improved with proper flow.
   Response: introduction flow has been improved. See revised document with track changes page 3-5

2. Please elaborate qualitative and quantitative mode of your study too, why you have select both?
   Response: to make a robust study and for comparison of results. Triangulation of methods was done. Qualitative methods helped us to dive into new findings on perceptions and pesticide stained tomatoes while quantitative methods helped us to quantify these findings.

   See the following articles and cite where needed.
   I. Saeed, M.F., Shaheen, M., Ahmad, I., Zakir, A., Nadeem, M., Chishti, A.A., Shahid, M., Bakhsh, K. and Damalas, C.A., 2017. Pesticide exposure in the local community of Vehari District in Pakistan: An assessment of knowledge and residues in human blood. Science of the Total Environment, 587, pp.137-144.
   II. Hayat, K., Afzal, M., Aqueel, M.A., Ali, S., Saeed, M.F., Qureshi, A.K., Ullah, M.I., Khan, Q.M., Naseem, M.T., Ashfaq, U. and Damalas, C.A., 2019. Insecticide toxic effects and blood biochemical alterations in occupationally exposed individuals in Punjab, Pakistan. Science of The Total Environment, 655, pp.102-111.
3. Elaborate what fruits and vegetables and what type of orchard are managed in this region. **Response:** type of fruit and vegetables grow included. see page 6 in the document with track changes.
4. What type sparing equipment's used.
   Response: Comment has been addressed by stating the commonly used spray equipment see page 6.
   What are climatic conditions? **Response:** comment addressed by stating the climate type, See page 6.
5. Your aim of stud is more generalized Please elaborate objectives of your study categorically and most specifically
   Response: objectives have been elaborated see page

Materials and Methods:

1. **House hold level, on average 117 participants were randomly selected and interviewed,** please explained in tabular form how many people were interviewed in each district?
2. Why have you expressed on average participants? Write the exact number even though they are different from one another by regions, making average is not appropriate and scientific in social sciences studies

   Response: presentation has been made in terms of whole number of samples per district. See page 7.

Results

1. The study registered 100% response from consumers What do you mean that?

   Response: the above confusing sentence which meant that all planned participants were interviewed has been rephrased. See page 10

2. 37.7years (SD±13.1, ranging from 18-88), it would be more interesting if you categorized the age, it may give you some differences among young and middle and old age people?

   Response: categorization was done fr below and above the mean age, but we preferred to report by the continuous variable here. As a side work, categorization was done at multi-variable analysis stage but it was not significant.

3. From the qualitative results, interviews involved tomato farmers 33.3% (12/36), tomato retail vendors 33.3% (12/36), and tomato wholesalers 33.3% (12/36) sampled in equal proportions in the 4 districts. i.e. three persons per category per district.

   This is very interesting for me Why you focused 12 participants from each, you can find more than this, it could be a potential participant, possibly you would have missed, what do you say??

   Response: we sampled purposively 9 participants not 12, and the reason for sampling is in the principles for conducting Focus Group Discussions (FGD), we were resource constrained and FGD require between 8 to 12 people, so 9 participants were just enough. We never missed what we wanted because data collection was based on the level of saturation of information received from the participants, which meant that interviews per questions were done until no new information was being collected from the participants.

4. Lower level, upper level, what do you mean by this? Please be more specific?

   Response: knowledge has been made specific by defining the two categories. Using bloom's knowledge taxonomy, a mean knowledge score was used as a cutoff value where a score below the mean was categorized as low knowledge and that above the mean as higher level of knowledge.
5. Discomfort and suspicion that the residues cause (details provided in S2 File and Fig1)., this is incomplete information??

   Response: sentence has been rephrased see page 12
6. Basing on the computed percentages, What do you mean?

Response: Computed here simply means calculated by stata, but to make it simple, the word 'computed' has been omitted from all phrases. See page 12

7. Fig 1 and 2 Average perceptions, What do you mean by average percentage??? I think, there is some serious mistake, please check, You have to present your data in percentage which is most appropriate method, making average of percentage in confusing???
   Response: average percentages have been used in a number of so long as the total numbers are the same.

8. Table 3: showing associations between awareness about pesticide residues in tomatoes with the consumer level of education and practice of buying stained tomatoes. Data presentation is confusing, make it more clear??
   Response: table title has been redefined see page 16.

9. residues/stains, I think most suitable term is residues??
   Response: well, residue is more compound but when we talk about the mancozeb stains are more visible than the dichlorvos residues which need chromatography test.

10. **Qualitative findings**, I would recommend presenting this part in a table form, it is difficult to follow it??
    Response: qualitative data has been presented in a tabular form.
11. The presentation of data needs to improve.
    Response: data presentation has been improved and arranged as per the requirements set by plosone journal

Discussion

1. Respondents of this study represent adult consumers experienced with tomato farming and with adequate level of education to well express their risk perception towards pesticide stained tomatoes., please rewrite this be specific what education levels are exactly?
   Response: education of participants has been well specified.
2. On a general note, Do not use this type of sentences?
   Response: sentence has been omitted from the writeup.
3. Stained is not right term to use in your whole article, avoid from.
   Response: We preferred to use the word stain in the context of the visibility of the pesticides residues to the consumer.
4. In this case due to the different situations, in Uganda unfortunately tomatoes are not labeled with the residual contents and benefits of low pesticide residual levels. Is there any type of labeling law, legislation exist, or commodities are sold openly in market???? For this you have to discuss something about overall characteristics of local people to explain in what category the study area lies with respect to education???
   Response: there are national laws requiring labeling the contents of products being sold on the market, although tomatoes are not formally covered. Discussion for this matter has been made on the basis of the local people.

5. Uganda lacking a food safety policy puts public health at stake for the pesticide exposures., Please make sure if there is really ***lacking a food safety policy???***
   Response: a food safety policy is lacking in Uganda, it's the public health act which talks about food safety and consumer protection but it's not detailed**.** The food and drug act covers also a few this where tomatoes and a number of vegetables are no covered.

6. our qualitative results by vendors and tomato farmers from the FGDs, indicate that the highly stained tomatoes are due to poor hybrid tomato seeds that need frequent spraying, vendors' demand from tomato growers to spray tomatoes before they sell but also a low level of literacy to understand the pesticide label information coupled with a wrong perception of tomato farmers that Mancozeb pesticide can harden the outer skin and increase tomato shelf life. It is from this misconception that tomato vendors only buy stained tomatoes presuming that these tomatoes will stay long on shelf, are healthy & free from the microbial contaminants., **Can you find out how this perception has been build-up????**
   Response: this may require another study, a phrase has been included in the manuscript suggesting a study to look through this.
