# Supplementary material for: Consumer risk perception towards pesticide-stained tomatoes in Uganda
Source: PLoS One. 2023 Dec 15;18(12):e0247740. doi: 10.1371/journal.pone.0247740 (PMC10723735; doi:10.1371/journal.pone.0247740)
Supplement: Attachment — Submitted filename: Review-Report_Uganda.docx [file pone.0247740.s009.docx]

**Dear Editor:**

I have reviewed an article titled “Consumer risk perception towards pesticide stained tomatoes in Uganda” The article data depict some interesting information; however, the scientific writing and presentation of data need to improve. This article needs substantial revision with proper sequence, I would advise to the authors please, read and follow some good articles to present your data.

Introduction

1. The introduction needs to improve with proper flow.
2. Please elaborate qualitative and quantitative mode of your study too, why you have select both?

See the following articles and cite where needed.

I. Saeed, M.F., Shaheen, M., Ahmad, I., Zakir, A., Nadeem, M., Chishti, A.A., Shahid, M., Bakhsh, K. and Damalas, C.A., 2017. Pesticide exposure in the local community of Vehari District in Pakistan: An assessment of knowledge and residues in human blood. Science of the Total Environment, 587, pp.137-144.
II. Hayat, K., Afzal, M., Aqueel, M.A., Ali, S., Saeed, M.F., Qureshi, A.K., Ullah, M.I., Khan, Q.M., Naseem, M.T., Ashfaq, U. and Damalas, C.A., 2019. Insecticide toxic effects and blood biochemical alterations in occupationally exposed individuals in Punjab, Pakistan. Science of The Total Environment, 655, pp.102-111.
3. Elaborate what fruits and vegetables and what type of orchard are managed in this region
4. What type sparing equipment’s used
5. What are climatic conditions?
6. Your aim of stud is more generalized Please elaborate objectives of your study categorically and most specifically

Materials and Methods:

1. **household level, on average 117 participants were randomly selected and interviewed,** please explained in tabular form how many people were interviewed in each district?
2. Why have you expressed on average participants? Write the exact number even though they are different from one another by regions, making average is not appropriate and scientific in social sciences studies

Results

1. The study registered 100% response from consumers What do you mean that?
2. 37.7years (SD±13.1, ranging from 18-88), it would be more interesting if you categorized the age, it may give you some differences among young and middle and old age people?
3. From the qualitative results, interviews involved tomato farmers

33.3% (12/36), tomato retail vendors 33.3% (12/36), and tomato wholesalers 33.3% (12/36)

sampled in equal proportions in the 4 districts. i.e. three persons per category per district.

**This is very interesting for me Why you focused 12 participants from each, you can find more than this, it could be a potential participant, possibly you would have missed, what do you say??**

4. Lower level, upper level, what do you mean by this? Please be more specific?
5. discomfort and suspicion that the residues cause (details provided in S2 File and Fig1)., this is incomplete information??
6. Basing on the computed percentages, What do you mean?
7. Fig 1 and 2 Average perceptions, What do you mean by average percentage??? I think, there is some serious mistake, please check, You have to present your data in percentage which is most appropriate method, making average of percentage in confusing???
8. Table 3: showing associations between awareness about pesticide residues in

tomatoes with the consumer level of education and practice of buying stained

tomatoes. Data presentation is confusing, make it more clear??

9. residues/stains, I think most suitable term is residues??
10. **Qualitative findings**, I would recommend presenting this part in a table form, it is difficult to follow it??
11. The presentation of data needs to improve.

Discussion

1. Respondents of this study represent adult consumers experienced with tomato farming and

with adequate level of education to well express their risk perception towards pesticide

stained tomatoes., please rewrite this be specific what education levels are exactly?

2. On a general note, Do not use this type of sentences?
3. Stained is not right term to use in your whole article, avoid from it
4. In this case due to the different situations, in Uganda unfortunately tomatoes are not labeled with the residual contents and benefits of low pesticide residual levels. Is there any type of labeling law, legislation exist, or commodities are sold openly in market???? For this you have to discuss something about overall characteristics of local people to explain in what category the study area lies with respect to education???
5. Uganda lacking a food safety policy puts public health at stake for the pesticide

exposures., Please make sure if there is really ***lacking a food safety policy???***

6. our qualitative results by vendors and tomato farmers from the FGDs,

indicate that the highly stained tomatoes are due to poor hybrid tomato seeds that need

frequent spraying, vendors' demand from tomato growers to spray tomatoes before they

sell but also a low level of literacy to understand the pesticide label information coupled

with a wrong perception of tomato farmers that Mancozeb pesticide can harden the outer

skin and increase tomato shelf life. It is from this misconception that tomato vendors only

buy stained tomatoes presuming that these tomatoes will stay long on shelf, are healthy &

free from the microbial contaminants., **Can you find out how this perception has been build-up????**
