# Supplementary material for: Consumer risk perception towards pesticide-stained tomatoes in Uganda
Source: PLoS One. 2023 Dec 15;18(12):e0247740. doi: 10.1371/journal.pone.0247740 (PMC10723735; doi:10.1371/journal.pone.0247740)
Supplement: Attachment — Submitted filename: Response to reviewers.docx [file pone.0247740.s010.docx]

**Dear Editor:**

I have reviewed an article titled “Consumer risk perception towards pesticide stained tomatoes in Uganda” The article data depict some interesting information; however, the scientific writing and presentation of data need to improve. This article needs substantial revision with proper sequence, I would advise to the authors please, read and follow some good articles to present your data.

Introduction

1. The introduction needs to be improved with proper flow.

**Response:** introduction flow has been improved. See revised document with track changes page 3-5

2. Please elaborate qualitative and quantitative mode of your study too, why you have select both?

**Response:** to make a robust study and for comparison of results. Triangulation of methods was done. Qualitative methods helped us to dive into new findings on perceptions and pesticide stained tomatoes while quantitative methods helped us to quantify these findings.

See the following articles and cite where needed.

I. Saeed, M.F., Shaheen, M., Ahmad, I., Zakir, A., Nadeem, M., Chishti, A.A., Shahid, M., Bakhsh, K. and Damalas, C.A., 2017. Pesticide exposure in the local community of Vehari District in Pakistan: An assessment of knowledge and residues in human blood. Science of the Total Environment, 587, pp.137-144.
II. Hayat, K., Afzal, M., Aqueel, M.A., Ali, S., Saeed, M.F., Qureshi, A.K., Ullah, M.I., Khan, Q.M., Naseem, M.T., Ashfaq, U. and Damalas, C.A., 2019. Insecticide toxic effects and blood biochemical alterations in occupationally exposed individuals in Punjab, Pakistan. Science of The Total Environment, 655, pp.102-111.
3. Elaborate what fruits and vegetables and what type of orchard are managed in this region. **Response:** type of fruit and vegetables grow included. see page 6 in the document with track changes.
4. What type sparing equipment’s used.

**Response:** Comment has been addressed by stating the commonly used spray equipment see page 6.

What are climatic conditions? **Response:** comment addressed by stating the climate type, See page 6.

5. Your aim of stud is more generalized Please elaborate objectives of your study categorically and most specifically

**Response**: objectives have been elaborated see page

Materials and Methods:

1. **House hold level, on average 117 participants were randomly selected and interviewed,** please explained in tabular form how many people were interviewed in each district?
2. Why have you expressed on average participants? Write the exact number even though they are different from one another by regions, making average is not appropriate and scientific in social sciences studies

**Response:** presentation has been made in terms of whole number of samples per district. See page 7.

Results

1. The study registered 100% response from consumers What do you mean that?

**Response:** the above confusing sentence which meant that all planned participants were interviewed has been rephrased. See page 10

2. 37.7years (SD±13.1, ranging from 18-88), it would be more interesting if you categorized the age, it may give you some differences among young and middle and old age people?

**Response:** categorization was done fr below and above the mean age, but we preferred to report by the continuous variable here. As a side work, categorization was done at multi-variable analysis stage but it was not significant.

3. From the qualitative results, interviews involved tomato farmers

33.3% (12/36), tomato retail vendors 33.3% (12/36), and tomato wholesalers 33.3% (12/36) sampled in equal proportions in the 4 districts. i.e. three persons per category per district.

**This is very interesting for me Why you focused 12 participants from each, you can find more than this, it could be a potential participant, possibly you would have missed, what do you say??**

**Response:** we sampled purposively 9 participants not 12, and the reason for sampling is in the principles for conducting Focus Group Discussions (FGD), we were resource constrained and FGD require between 8 to 12 people, so 9 participants were just enough. We never missed what we wanted because data collection was based on the level of saturation of information received from the participants, which meant that interviews per questions were done until no new information was being collected from the participants.

4. Lower level, upper level, what do you mean by this? Please be more specific?

**Response:** knowledge has been made specific by defining the two categories. Using bloom’s knowledge taxonomy, a mean knowledge score was used as a cutoff value where a score below the mean was categorized as low knowledge and that above the mean as higher level of knowledge.

5. Discomfort and suspicion that the residues cause (details provided in S2 File and Fig1)., this is incomplete information??

**Response:** sentence has been rephrased see page 12

6. Basing on the computed percentages, What do you mean?

**Response:** Computed here simply means calculated by stata, but to make it simple, the word ‘computed’ has been omitted from all phrases. See page 12

7. Fig 1 and 2 Average perceptions, What do you mean by average percentage??? I think, there is some serious mistake, please check, You have to present your data in percentage which is most appropriate method, making average of percentage in confusing???

**Response:** average percentages have been used in a number of so long as the total numbers are the same.

8. Table 3: showing associations between awareness about pesticide residues in

tomatoes with the consumer level of education and practice of buying stained

tomatoes. Data presentation is confusing, make it more clear??

**Response:** table title has been redefined see page 16.

9. residues/stains, I think most suitable term is residues??

**Response:** well, residue is more compound but when we talk about the mancozeb stains are more visible than the dichlorvos residues which need chromatography test.

10. **Qualitative findings**, I would recommend presenting this part in a table form, it is difficult to follow it??

**Response:** qualitative data has been presented in a tabular form.

11. The presentation of data needs to improve.

**Response:** data presentation has been improved and arranged as per the requirements set by plosone journal

**Discussion**

1. Respondents of this study represent adult consumers experienced with tomato farming and

with adequate level of education to well express their risk perception towards pesticide

stained tomatoes., please rewrite this be specific what education levels are exactly?

**Response:** education of participants has been well specified.

2. On a general note, Do not use this type of sentences?

**Response:** sentence has been omitted from the writeup.

3. Stained is not right term to use in your whole article, avoid from.

**Response:** We preferred to use the word stain in the context of the visibility of the pesticides residues to the consumer.

4. In this case due to the different situations, in Uganda unfortunately tomatoes are not labeled with the residual contents and benefits of low pesticide residual levels. Is there any type of labeling law, legislation exist, or commodities are sold openly in market???? For this you have to discuss something about overall characteristics of local people to explain in what category the study area lies with respect to education???

**Response:** there are national laws requiring labeling the contents of products being sold on the market, although tomatoes are not formally covered. Discussion for this matter has been made on the basis of the local people.

5. Uganda lacking a food safety policy puts public health at stake for the pesticide

exposures., Please make sure if there is really ***lacking a food safety policy???***

**Response:** a food safety policy is lacking in Uganda, it’s the public health act which talks about food safety and consumer protection but it’s not detailed**.** The food and drug act covers also a few this where tomatoes and a number of vegetables are no covered.

6. our qualitative results by vendors and tomato farmers from the FGDs,

indicate that the highly stained tomatoes are due to poor hybrid tomato seeds that need

frequent spraying, vendors' demand from tomato growers to spray tomatoes before they

sell but also a low level of literacy to understand the pesticide label information coupled

with a wrong perception of tomato farmers that Mancozeb pesticide can harden the outer

skin and increase tomato shelf life. It is from this misconception that tomato vendors only

buy stained tomatoes presuming that these tomatoes will stay long on shelf, are healthy &

free from the microbial contaminants., **Can you find out how this perception has been build-up????**

**Response:** this may require another study, a phrase has been included in the manuscript suggesting a study to look through this.
