# Supplementary material for: Consumer risk perception towards pesticide-stained tomatoes in Uganda
Source: PLoS One. 2023 Dec 15;18(12):e0247740. doi: 10.1371/journal.pone.0247740 (PMC10723735; doi:10.1371/journal.pone.0247740)
Supplement: Attachment — Submitted filename: Reviewer comment.docx [file pone.0247740.s013.docx]

**Reviewer comment**

The author has presented a well written article (Consumer risk perception towards pesticide stained tomatoes in Uganda), detailed, and supported by solid analysis. However, reviewers must make some suggestions to improve this manuscript's quality, including:

1. The author needs to add novelty and importance to this study. In addition, the authors should mention the contribution of this study to theoretical and practical development.
2. The manuscript does not have a strong theoretical foundation. I recommend the author to add a theory section.
3. The author must include a research framework (in the form of figures) in the methods section.
4. The author must explain why the number of respondents in each district is different and disproportionate to the population in each region
5. The author needs to add reliability and validity tests
6. The author must adjust the structure of the results section according to the structure in the method (optimism, pessimism and trust)
7. The author states that there are 468 respondents, but the number of respondents in each discussion subsection is different. Please be consistent! The author has to calculate everything again and also make sure all percentages add up to 100%.
8. The author needs to add activities that have been conducted by the government and the community of Uganda to control the use of pesticides in the discussion section
9. The author needs to add limitations and further research in the conclusion section.

Finally, I hope these various suggestions can help you improve this manuscript quality and be published in the Plos One journal. Good luck!
