# Supplementary material for: Consumer risk perception towards pesticide-stained tomatoes in Uganda
Source: PLoS One. 2023 Dec 15;18(12):e0247740. doi: 10.1371/journal.pone.0247740 (PMC10723735; doi:10.1371/journal.pone.0247740)
Supplement: Attachment — Submitted filename: PLos One - Copy.docx [file pone.0247740.s014.docx]

**Title: Consumer risk perception towards pesticide-stained tomatoes in Uganda**

**Manuscript Number: PONE-D-21-04826R1**

The manuscript entitled Consumer risk perception towards pesticide-stained tomatoes in Uganda is a good contribution to the existing body of knowledge in the related discipline. The paper used a mixed methods cross-sectional dataset of 468 household consumers in four regions of Uganda. The paper concluded that Consumer risk perception on pesticide-stained tomatoes among Ugandan consumers ranked low with majority of the consumers buying tomatoes stained with pesticide residues due to lack of an alternative. I am satisfied with the paper to be published with the following minor changes.

- I found English and sentence structuring throughout the paper a bit week that is needed to be addressed.
- Keywords may be changed
- In the methodology section the functional form of the model used may be explained and give the reference of the model adapted from
- I think discussion section need more references/literature cited.
- All the results should be balanced with some references with prior studies and may be discussed in the discussion section.
