# Supplementary material for: Consumer risk perception towards pesticide-stained tomatoes in Uganda
Source: PLoS One. 2023 Dec 15;18(12):e0247740. doi: 10.1371/journal.pone.0247740 (PMC10723735; doi:10.1371/journal.pone.0247740)
Supplement: Attachment — Submitted filename: PONE-D-21-04826R1_reviewer-EM_Report.docx [file pone.0247740.s016.docx]

Dear Editor,

Thank you for considering me to review the manuscript entitled **“Consumer risk perception towards pesticide stained tomatoes in Uganda”, PONE-D-21-04826R1.**

I have reviewed the manuscript and note that the authors have addressed most of the comments raised in earlier review.

Nevertheless, I have a number of minor comments that I believe if addressed will add value to the article and clarify issues for readers. My comments are provided below and are also highlighted in the manuscript that I will attach.

I thank you and look forward to working with you in future.

Kind regards

Elijah N Muange (PhD)

Reviewer

3^rd^ October, 2022

Title: Could be edited to include **attitude**, thus “Consumer attitude and risk perception ...”

**Abstract**

1. It is indicated that “…European Union Maximum Residue Limits (used as a standard in Uganda)”. This
2. If this standard has been domesticated in Uganda, then authors could just say the pesticide residues are above the permitted MRLs in the country.
3. Total participants in FGD should also have been mentioned
4. It is good to mention the regions where the study was conducted
5. The project should be introduced very briefly so that readers may relate the study to the project. For example, what was the goal or objectives/activities?

**Introduction**

Page5

Should be specified that it is “pesticide” regulations

Page6

- Although the general problem has been articulated on a global scale, the case for Uganda is not very explicit. What are the literature gaps this study seeks to fill? What have previous studies done and what remains to be done?

**Methods**

Page6

- Did the study target only households that participated in the project? How were sampling frames constructed?
- As mentioned above (abstract), this project needs to be elaborated so that readers can relate it to the study (aims, activities, targeting, etc)

**Study design**

P7

- Which sub counties were involved?
- Were these numbers proportional to district population or aim was to have equal numbers per district?
- In the results section, the respondents are 17. No explanation is given on responses of the other 19 or why they are missing from the analysis.

**Results**

P11

- It may also have been good to describe the sample involved in FGD. Who were they - age, education level, gender, etc.
- Level of income could be a good determinant of preference and purchase decisions. It would have been interesting to capture it. alternately, there was a variable on occupation

Rable1:

- Male and female: these total to 467 and not 468, one is missing

P12

- Do authors use **stain** to mean **residues** contained in the tomato, or physical staining outside the tomato fruit? This should be clarified from the start and throughout the document. Earlier reviewer raised similar concerns. **Residuals** is also used elsewhere. It would be good to distinguish these terms and use them appropriately.

**Qualitative findings**

P18

- These should be 36 going by the information in the methods section

P20

- The highlighted area, is it *residue* or *stain*?

**Discussion**

- The second paragraph is merely descriptive of the sample characteristics and may not be necessary in the discussion section. The section should go straight to the main results

P23

- A study is mentioned, but the place it was carried out is not mentioned.

General: It could have been interesting to find out whether participating in the project had any effect on attitudes or risk perception.
