# Supplementary material for: Consumer risk perception towards pesticide-stained tomatoes in Uganda
Source: PLoS One. 2023 Dec 15;18(12):e0247740. doi: 10.1371/journal.pone.0247740 (PMC10723735; doi:10.1371/journal.pone.0247740)
Supplement: Attachment — Submitted filename: Response to Reviewers.docx [file pone.0247740.s017.docx]

Dear editorial manager,

I take this opportunity once again to so much thank the reviewers of this manuscript for the insights rendered and tireless corrections pointed out. These can never be taken for granted.

We have taken time responding to each of the comments raised by the different reviewers and happy to lay them to you for further guidance.

Please find below a list of comments and respective responses. Please make all your references to the “Revised manuscript with track changes” document for changes made.

Thank you!

**Reviewer 1 comments and response**

**Title: Consumer risk perception towards pesticide-stained tomatoes in Uganda**

**Manuscript Number: PONE-D-21-04826R1**

The manuscript entitled Consumer risk perception towards pesticide-stained tomatoes in Uganda is a good contribution to the existing body of knowledge in the related discipline. The paper used a mixed methods cross-sectional dataset of 468 household consumers in four regions of Uganda. The paper concluded that Consumer risk perception on pesticide-stained tomatoes among Ugandan consumers ranked low with majority of the consumers buying tomatoes stained with pesticide residues due to lack of an alternative. I am satisfied with the paper to be published with the following minor changes.

- I found English and sentence structuring throughout the paper a bit week that is needed to be addressed.

**Response:** the whole manuscript has been reviewed and English weakness addressed refer to revised manuscript with track changes .docx.

- Keywords may be changed

**Response:** keywords have been added and others maintained unless suggestions for change are provided by the reviewers.

- In the methodology section the functional form of the model used may be explained and give the reference of the model adapted from.

**Response:** research model used has been inserted with reference and model theory explained. see methodology section.

- I think discussion section need more references/literature cited.

**Response:** more references to the results have been inserted and discussed. See document with track changes page. References have been provided basing on what has been discussed unless the reviewer is specific on where references should be provided.

- All the results should be balanced with some references with prior studies and may be discussed in the discussion section.

**Response:** refer to response above.

**Reviewer 2 comments and responses**

The author has presented a well written article (Consumer risk perception towards pesticide stained tomatoes in Uganda), detailed, and supported by solid analysis. However, reviewers must make some suggestions to improve this manuscript's quality, including:

1. The author needs to add novelty and importance to this study. In addition, the authors should mention the contribution of this study to theoretical and practical development.

**Response:** a paragraph has been inserted stating on contributions made by this study to existing knowledge base of consumer risk perception and also results being used as a justification for the establishment of a nation pesticide residue monitoring program for Uganda. See last paragraph of introduction page 8.

2. The manuscript does not have a strong theoretical foundation. I recommend the author to add a theory section.

**Response:** theoretical section has been added to the manuscript see page9. At the beginning of the methodology section.

3. The author must include a research framework (in the form of figures) in the methods section.

**Response:** a research framework narrative section been added to the manuscript with a reference to figure 1 which has also been revised to include the knowledge question. See page 9 for the tracked document.

4. The author must explain why the number of respondents in each district is different and disproportionate to the population in each region.

**Response:** numbers being different was due to excessive sampling done in some sub counties. This has no effect on the results it instead increased accuracy since all the targets per sampling unit was met. Our sample size had a target of 423 and in the end 468 participants were interviewed, i.e more than 100% of the targeted.

5. The author needs to add reliability and validity tests.

**Response:** we couldn’t perform these tests as we felt that they are not a must.

6. The author must adjust the structure of the results section according to the structure in the method (optimism, pessimism and trust)

**Response:** results of the study have been presented in the same manner of optimism, pessimism and trust see page 13-15.

7. The author states that there are 468 respondents, but the number of respondents in each discussion subsection is different. Please be consistent! The author has to calculate everything again and also make sure all percentages add up to 100%.

**Response:** that only applies to question which were not supposed to be answered by the whole sample forexample for questions which followed a screening question as indicated in fig3. there has been consistency in the total number of respondents presented in each section.

8. The author needs to add activities that have been conducted by the government and the community of Uganda to control the use of pesticides in the discussion section.

**Response:** a paragraph has been stated on this see page..31

9. The author needs to add limitations and further research in the conclusion section.

**Response:** study limitation other research recommendations have been included see page 32

Finally, I hope these various suggestions can help you improve this manuscript quality and be published in the Plos One journal. Good luck!

Dear Editor,

Thank you for considering me to review the manuscript entitled **“Consumer risk perception towards pesticide stained tomatoes in Uganda”, PONE-D-21-04826R1.**

I have reviewed the manuscript and note that the authors have addressed most of the comments raised in earlier review.

Nevertheless, I have a number of minor comments that I believe if addressed will add value to the article and clarify issues for readers. My comments are provided below and are also highlighted in the manuscript that I will attach.

I thank you and look forward to working with you in future.

Kind regards

Elijah N Muange (PhD)

Reviewer

3^rd^ October, 2022

Title: Could be edited to include **attitude**, thus “Consumer attitude and risk perception ...”

**Abstract**

1. It is indicated that “…European Union Maximum Residue Limits (used as a standard in Uganda)”. **Response:** These MRL used only during the vetting process for exports of foods from Uganda.
2. If this standard has been domesticated in Uganda, then authors could just say the pesticide residues are above the permitted MRLs in the country.

**Response:** comment has been adopted and rectified refer to abstract.

3. Total participants in FGD should also have been mentioned.

**Response:** total FGD participants have been mentioned in the abstract.

4. It is good to mention the regions where the study was conducted

**Response**: regions have been inserted in the manuscript text and felt not necessary to still mention them in the summary abstract.

5. The project should be introduced very briefly so that readers may relate the study to the project. For example, what was the goal or objectives/activities?

**Response:** the project brief has been included in the manuscript but not in the abstract.

**Introduction**

Page5

Should be specified that it is “pesticide” regulations

**Response:** specification of the regulation for pesticide application has been inserted see page 8

Page6

- Although the general problem has been articulated on a global scale, the case for Uganda is not very explicit. What are the literature gaps this study seeks to fill? What have previous studies done and what remains to be done?

**Response:** literature gaps have been inserted with references. See introduction section

**Methods**

Page6

- Did the study target only households that participated in the project? How were sampling frames constructed?

**Response:** sampling procedure has been inserted refer to methodology, study design on page14

- As mentioned above (abstract), this project needs to be elaborated so that readers can relate it to the study (aims, activities, targeting, etc)

**Response:** this is not a must since the project did this study as a stand-alone for advocacy purposes on consumer protection.

**Study design**

P7

- Which sub counties were involved?

**Response:** Sub counties have been inserted; under study population subsection

- Were these numbers proportional to district population or aim was to have equal numbers per district?

**Response:** equal numbers per district was the aim, proportionate sampling was not employed.

- In the results section, the respondents are 17. No explanation is given on responses of the other 19 or why they are missing from the analysis.
- Response: in qualitative interviews, response saturation is followed, the 2 respondents didn’t have a different view of the question.

**Results**

P11

- It may also have been good to describe the sample involved in FGD. Who were they  - age, education level, gender, etc.

**Response:** sorry! these was not captured.

- Level of income could be a good determinant of preference and purchase decisions. It would have been interesting to capture it. alternately, there was a variable on occupation.

**Response:** this was capture but later found to be biased due to sensitivity of the question and implications since most respondents’ fear taxing income, they would give wrong income values. Yes occupation was captured and been used in the analysis and discussion.

Rable1:

- Male and female: these total to 467 and not 468, one is missing

**Response:** missing male has been added.

P12

- Do authors use **stain** to mean **residues** contained in the tomato, or physical staining outside the tomato fruit? This should be clarified from the start and throughout the document. Earlier reviewer raised similar concerns. **Residuals** is also used elsewhere. It would be good to distinguish these terms and use them appropriately.

**Response:** Residues and stains are used interchangeably, stains are a type of physical residue. This has been defined in the document in the introduction section.

**Qualitative findings**

P18

- These should be 36 going by the information in the methods section

**Response:** yes although not all the respondents had to answer the question answering depended much on level of saturation, until no more new responses were captured.

P20

- The highlighted area, is it *residue* or *stain*?

**Response:** please refer to comment above on pesticide residue and stain.

**Discussion**

- The second paragraph is merely descriptive of the sample characteristics and may not be necessary in the discussion section. The section should go straight to the main results

**Response:** the paragraph gives the reader a description of the sample under study

P23

- A study is mentioned, but the place it was carried out is not mentioned.

**Response:** study areas has been mentioned under study area and population under methodology section.

General: It could have been interesting to find out whether participating in the project had any effect on attitudes or risk perception.

**Response:** yes, though it was not the aim by then and therefore not captured, thank you!
