# Supplementary material for: Consumer risk perception towards pesticide-stained tomatoes in Uganda
Source: PLoS One. 2023 Dec 15;18(12):e0247740. doi: 10.1371/journal.pone.0247740 (PMC10723735; doi:10.1371/journal.pone.0247740)
Supplement: Attachment — Submitted filename: Response to Reviewers.docx..docx [file pone.0247740.s018.docx]

**Dear editorial manager,**

Thank you for taking up this role again in reviewing our article, it's unfortunate that some of the comments were not addressed. Please look at the corrections made this time round. I hope the correction will meet your expectations and that of the research fraternity.

We have taken time to respond to each of the comments raised by the different reviewers and are happy to lay them to you for further guidance.

Please find below a list of comments and respective responses for reviewers #5 and #7. Please make all your references to the “Revised manuscript with track changes” document for changes made.

Thank you!

**Title: Consumer risk perception towards pesticide-stained Tomatoes in Uganda**

**Manuscript Number: PONE-D-21-04826R1**

**RE:** **PLOS ONE Decision: Revision required [PONE-D-21-04826R2**

**Reviewer comments and response**

**Reviewer #5: Dear authors,**

Thank you for your efforts to improve and improve the quality of this manuscript. However, I am disappointed with your response that does not meet my suggestions.

1. I asked you to write a novelty for this manuscript, but I could not find it in your edited manuscript

**Response:** a paragraph has been edited, stating contributions made by this study to the existing knowledge base of consumer risk perception and also results being used as a justification for the establishment of a national pesticide residue monitoring program for Uganda. See the last paragraph of the introduction page 7.

2. You say added a theoretical section on page 9 (at the beginning of the methodology section). I have read this manuscript carefully and searched for the location of the theory according to your explanation, but I did not find it in either the track changes or no track changes of this manuscript. This is what makes this manuscript undirected because the selection of variables is not based on theory and the authors are unable to explain the contribution of this manuscript to the development of theory.

**Response:** A theoretical section has been inserted (just after introduction), please refer to page 7 of the tracked version.

3. I understand your sample very much. But you should be able to make arguments why the number of samples in each subregion is different. Is the population beneficiary of the program in each sub-region different so that the number of samples in 1 sub-region is higher than in other sub-regions?

**Response:** The sample deviated to the fact that some sub-counties had more participants than others, this was due to the difference in the social economic status and population of urban, rural and peri-urban areas selected from each district.

4. You stated the reliability and validity tests were not mandatory. How do you ensure that your questionnaire is valid and reliable?

**Response:** sorry about the oversight**,** the questionnaire had been adopted from another study, and we did not see it necessary to do the validity tests, maybe next time.

5. I cannot find any limitations to this study and recommendations for further studies

**Response:** A section on study limitations has been inserted on page 29 see tracked version of the document just before the conclusion section.

Reviewer #7:

Dear Editor,

The authors have addressed the comments raised satisfactorily and the document is much improved.

Just a small thing - the statistical software used in Stata, authors could delete Corp since StataCorp is the company.

**Response:** This has been done as shown in the tracked version of the manuscript pages 3 and 11.
