# Supplementary material for: Consumer risk perception towards pesticide-stained tomatoes in Uganda
Source: PLoS One. 2023 Dec 15;18(12):e0247740. doi: 10.1371/journal.pone.0247740 (PMC10723735; doi:10.1371/journal.pone.0247740)
Supplement: Peer review history

# Peer review: Decision Letter 0

**DOI:** 10.1371/journal.pone.0247740.r001

**Authors:** Pankaj B. Pathare

17 Aug 2021

PONE-D-21-04826

Consumer risk perception towards pesticide stained tomatoes in Uganda.

PLOS ONE

Dear Dr. Sekabojja,

Thank you for submitting your manuscript to PLOS ONE. After careful consideration, we feel that it has merit but does not fully meet PLOS ONE’s publication criteria as it currently stands. Therefore, we invite you to submit a revised version of the manuscript that addresses the points raised during the review process.

Please submit your revised manuscript by Oct 01 2021 11:59PM. If you will need more time than this to complete your revisions, please reply to this message or contact the journal office at plosone@plos.org. When you're ready to submit your revision, log on to https://www.editorialmanager.com/pone/ and select the 'Submissions Needing Revision' folder to locate your manuscript file.

Please include the following items when submitting your revised manuscript:

- A rebuttal letter that responds to each point raised by the academic editor and reviewer(s). You should upload this letter as a separate file labeled 'Response to Reviewers'.
- A marked-up copy of your manuscript that highlights changes made to the original version. You should upload this as a separate file labeled 'Revised Manuscript with Track Changes'.
- An unmarked version of your revised paper without tracked changes. You should upload this as a separate file labeled 'Manuscript'.

If you would like to make changes to your financial disclosure, please include your updated statement in your cover letter. Guidelines for resubmitting your figure files are available below the reviewer comments at the end of this letter.

If applicable, we recommend that you deposit your laboratory protocols in protocols.io to enhance the reproducibility of your results. Protocols.io assigns your protocol its own identifier (DOI) so that it can be cited independently in the future. For instructions see: http://journals.plos.org/plosone/s/submission-guidelines#loc-laboratory-protocols. Additionally, PLOS ONE offers an option for publishing peer-reviewed Lab Protocol articles, which describe protocols hosted on protocols.io. Read more information on sharing protocols at https://plos.org/protocols?utm_medium=editorial-email&utm_source=authorletters&utm_campaign=protocols.

We look forward to receiving your revised manuscript.

Kind regards,

Pankaj B. Pathare

Academic Editor

PLOS ONE

Journal Requirements:

When submitting your revision, we need you to address these additional requirements.

1. Please ensure that your manuscript meets PLOS ONE's style requirements, including those for file naming. The PLOS ONE style templates can be found at 

https://journals.plos.org/plosone/s/file?id=wjVg/PLOSOne_formatting_sample_main_body.pdf and 

https://journals.plos.org/plosone/s/file?id=ba62/PLOSOne_formatting_sample_title_authors_affiliations.pdf

2. In your Data Availability statement, you have not specified where the minimal data set underlying the results described in your manuscript can be found. PLOS defines a study's minimal data set as the underlying data used to reach the conclusions drawn in the manuscript and any additional data required to replicate the reported study findings in their entirety. All PLOS journals require that the minimal data set be made fully available. For more information about our data policy, please see http://journals.plos.org/plosone/s/data-availability.

Upon re-submitting your revised manuscript, please upload your study’s minimal underlying data set as either Supporting Information files or to a stable, public repository and include the relevant URLs, DOIs, or accession numbers within your revised cover letter. For a list of acceptable repositories, please see http://journals.plos.org/plosone/s/data-availability#loc-recommended-repositories. Any potentially identifying patient information must be fully anonymized.

Important: If there are ethical or legal restrictions to sharing your data publicly, please explain these restrictions in detail. Please see our guidelines for more information on what we consider unacceptable restrictions to publicly sharing data: http://journals.plos.org/plosone/s/data-availability#loc-unacceptable-data-access-restrictions. Note that it is not acceptable for the authors to be the sole named individuals responsible for ensuring data access.

We will update your Data Availability statement to reflect the information you provide in your cover letter.

Reviewers' comments:

Reviewer's Responses to Questions

**Comments to the Author**

1. Is the manuscript technically sound, and do the data support the conclusions?

The manuscript must describe a technically sound piece of scientific research with data that supports the conclusions. Experiments must have been conducted rigorously, with appropriate controls, replication, and sample sizes. The conclusions must be drawn appropriately based on the data presented. 

Reviewer #1: Partly

Reviewer #2: Yes

**********

2. Has the statistical analysis been performed appropriately and rigorously? 

Reviewer #1: I Don't Know

Reviewer #2: Yes

**********

3. Have the authors made all data underlying the findings in their manuscript fully available?

The PLOS Data policy requires authors to make all data underlying the findings described in their manuscript fully available without restriction, with rare exception (please refer to the Data Availability Statement in the manuscript PDF file). The data should be provided as part of the manuscript or its supporting information, or deposited to a public repository. For example, in addition to summary statistics, the data points behind means, medians and variance measures should be available. If there are restrictions on publicly sharing data—e.g. participant privacy or use of data from a third party—those must be specified.

Reviewer #1: Yes

Reviewer #2: Yes

**********

4. Is the manuscript presented in an intelligible fashion and written in standard English?

PLOS ONE does not copyedit accepted manuscripts, so the language in submitted articles must be clear, correct, and unambiguous. Any typographical or grammatical errors should be corrected at revision, so please note any specific errors here.

Reviewer #1: No

Reviewer #2: No

**********

5. Review Comments to the Author

Please use the space provided to explain your answers to the questions above. You may also include additional comments for the author, including concerns about dual publication, research ethics, or publication ethics. (Please upload your review as an attachment if it exceeds 20,000 characters)

Reviewer #1: Risk perception studies in agriculture are fascinating, especially when is combined with qualitaitve techniques. I did not fully understand that combination. It seems like there are two sub-research going on, but you need to figure out how one fully supports the other one findings.

You need to think more how to present your results, the way you did so is vague and hard to follow it.

Reviewer #2: Dear Authors:

I have reviewed an article titled “Consumer risk perception towards pesticide stained tomatoes in Uganda” The article data depict some interesting information; however, the scientific writing and presentation of data need to improve. This article needs substantial revision with proper sequence, I would advise to the authors please, read and follow some good articles to present your data.

Introduction

1. The introduction needs to improve with proper flow.

2. Please elaborate qualitative and quantitative mode of your study too, why you have select both?

See the following articles and cite where needed.

I. Saeed, M.F., Shaheen, M., Ahmad, I., Zakir, A., Nadeem, M., Chishti, A.A., Shahid, M., Bakhsh, K. and Damalas, C.A., 2017. Pesticide exposure in the local community of Vehari District in Pakistan: An assessment of knowledge and residues in human blood. Science of the Total Environment, 587, pp.137-144.

II. Hayat, K., Afzal, M., Aqueel, M.A., Ali, S., Saeed, M.F., Qureshi, A.K., Ullah, M.I., Khan, Q.M., Naseem, M.T., Ashfaq, U. and Damalas, C.A., 2019. Insecticide toxic effects and blood biochemical alterations in occupationally exposed individuals in Punjab, Pakistan. Science of The Total Environment, 655, pp.102-111.

3. Elaborate what fruits and vegetables and what type of orchard are managed in this region

4. What type sparing equipment’s used

5. What are climatic conditions?

6. Your aim of stud is more generalized Please elaborate objectives of your study categorically and most specifically

Materials and Methods:

1. household level, on average 117 participants were randomly selected and interviewed, please explained in tabular form how many people were interviewed in each district?

2. Why have you expressed on average participants? Write the exact number even though they are different from one another by regions, making average is not appropriate and scientific in social sciences studies

Results

1. The study registered 100% response from consumers What do you mean that?

2. 37.7years (SD±13.1, ranging from 18-88), it would be more interesting if you categorized the age, it may give you some differences among young and middle and old age people?

3. From the qualitative results, interviews involved tomato farmers

33.3% (12/36), tomato retail vendors 33.3% (12/36), and tomato wholesalers 33.3% (12/36)

sampled in equal proportions in the 4 districts. i.e. three persons per category per district.

This is very interesting for me Why you focused 12 participants from each, you can find more than this, it could be a potential participant, possibly you would have missed, what do you say??

4. Lower level, upper level, what do you mean by this? Please be more specific?

5. discomfort and suspicion that the residues cause (details provided in S2 File and Fig1)., this is incomplete information??

6. Basing on the computed percentages, What do you mean?

7. Fig 1 and 2 Average perceptions, What do you mean by average percentage??? I think, there is some serious mistake, please check, You have to present your data in percentage which is most appropriate method, making average of percentage in confusing???

8. Table 3: showing associations between awareness about pesticide residues in

tomatoes with the consumer level of education and practice of buying stained

tomatoes. Data presentation is confusing, make it more clear??

9. residues/stains, I think most suitable term is residues??

10. Qualitative findings, I would recommend presenting this part in a table form, it is difficult to follow it??

11. The presentation of data needs to improve.

Discussion

1. Respondents of this study represent adult consumers experienced with tomato farming and

with adequate level of education to well express their risk perception towards pesticide

stained tomatoes., please rewrite this be specific what education levels are exactly?

2. On a general note, Do not use this type of sentences?

3. Stained is not right term to use in your whole article, avoid from it

4. In this case due to the different situations, in Uganda unfortunately tomatoes are not labeled with the residual contents and benefits of low pesticide residual levels. Is there any type of labeling law, legislation exist, or commodities are sold openly in market???? For this you have to discuss something about overall characteristics of local people to explain in what category the study area lies with respect to education???

5. Uganda lacking a food safety policy puts public health at stake for the pesticide

exposures., Please make sure if there is really lacking a food safety policy???

6. our qualitative results by vendors and tomato farmers from the FGDs,

indicate that the highly stained tomatoes are due to poor hybrid tomato seeds that need

frequent spraying, vendors' demand from tomato growers to spray tomatoes before they

sell but also a low level of literacy to understand the pesticide label information coupled

with a wrong perception of tomato farmers that Mancozeb pesticide can harden the outer

skin and increase tomato shelf life. It is from this misconception that tomato vendors only

buy stained tomatoes presuming that these tomatoes will stay long on shelf, are healthy &

free from the microbial contaminants., Can you find out how this perception has been build-up????

**********

6. PLOS authors have the option to publish the peer review history of their article (what does this mean?). If published, this will include your full peer review and any attached files.

If you choose “no”, your identity will remain anonymous but your review may still be made public.

**Do you want your identity to be public for this peer review?** For information about this choice, including consent withdrawal, please see our Privacy Policy.

Reviewer #1: No

Reviewer #2: **Yes: **Dr. Muhammed Farhan Saeed, (Ph.D, Germany; Post-Doctorate, China)

Assistant Professor

Department of Environmental Sciences

COMSATS University Islamabad, Vehari Campus, 61100 Vehari (Pakistan)

[NOTE: If reviewer comments were submitted as an attachment file, they will be attached to this email and accessible via the submission site. Please log into your account, locate the manuscript record, and check for the action link "View Attachments". If this link does not appear, there are no attachment files.]

While revising your submission, please upload your figure files to the Preflight Analysis and Conversion Engine (PACE) digital diagnostic tool, https://pacev2.apexcovantage.com/. PACE helps ensure that figures meet PLOS requirements. To use PACE, you must first register as a user. Registration is free. Then, login and navigate to the UPLOAD tab, where you will find detailed instructions on how to use the tool. If you encounter any issues or have any questions when using PACE, please email PLOS at figures@plos.org. Please note that Supporting Information files do not need this step.

Attachment Submitted filename: Review-Report_Uganda.docx Click here for additional data file.

# Author comment: Author response to Decision Letter 0

**DOI:** 10.1371/journal.pone.0247740.r002

25 Mar 2022

We Thank the reviewers for the wonderful comments and insights, they really important in improving this piece of research.

Attachment Submitted filename: Response to reviewers.docx Click here for additional data file.

# Peer review: Decision Letter 1

**DOI:** 10.1371/journal.pone.0247740.r003

**Authors:** Alejandro Vega-Muñoz

5 Oct 2022

PONE-D-21-04826R1Consumer risk perception towards pesticide stained tomatoes in Uganda.PLOS ONE

Dear Dr. Sekabojja,

Thank you for submitting your manuscript to PLOS ONE. After careful consideration, we feel that it has merit but does not fully meet PLOS ONE’s publication criteria as it currently stands. Therefore, we invite you to submit a revised version of the manuscript that addresses the points raised during the review process.

Please submit your revised manuscript by Nov 19 2022 11:59PM. If you will need more time than this to complete your revisions, please reply to this message or contact the journal office at plosone@plos.org. When you're ready to submit your revision, log on to https://www.editorialmanager.com/pone/ and select the 'Submissions Needing Revision' folder to locate your manuscript file.

Please include the following items when submitting your revised manuscript:

- A rebuttal letter that responds to each point raised by the academic editor and reviewer(s). You should upload this letter as a separate file labeled 'Response to Reviewers'.
- A marked-up copy of your manuscript that highlights changes made to the original version. You should upload this as a separate file labeled 'Revised Manuscript with Track Changes'.
- An unmarked version of your revised paper without tracked changes. You should upload this as a separate file labeled 'Manuscript'.

If you would like to make changes to your financial disclosure, please include your updated statement in your cover letter. Guidelines for resubmitting your figure files are available below the reviewer comments at the end of this letter.

If applicable, we recommend that you deposit your laboratory protocols in protocols.io to enhance the reproducibility of your results. Protocols.io assigns your protocol its own identifier (DOI) so that it can be cited independently in the future. For instructions see: https://journals.plos.org/plosone/s/submission-guidelines#loc-laboratory-protocols. Additionally, PLOS ONE offers an option for publishing peer-reviewed Lab Protocol articles, which describe protocols hosted on protocols.io. Read more information on sharing protocols at https://plos.org/protocols?utm_medium=editorial-email&utm_source=authorletters&utm_campaign=protocols.

We look forward to receiving your revised manuscript.

Kind regards,

Alejandro Vega-Muñoz, Ph.D.

Academic Editor

PLOS ONE

[Note: HTML markup is below. Please do not edit.]

Reviewers' comments:

Reviewer's Responses to Questions

**Comments to the Author**

1. If the authors have adequately addressed your comments raised in a previous round of review and you feel that this manuscript is now acceptable for publication, you may indicate that here to bypass the “Comments to the Author” section, enter your conflict of interest statement in the “Confidential to Editor” section, and submit your "Accept" recommendation.

Reviewer #1: All comments have been addressed

Reviewer #3: (No Response)

Reviewer #4: (No Response)

Reviewer #5: All comments have been addressed

Reviewer #6: All comments have been addressed

Reviewer #7: (No Response)

Reviewer #8: (No Response)

**********

2. Is the manuscript technically sound, and do the data support the conclusions?

The manuscript must describe a technically sound piece of scientific research with data that supports the conclusions. Experiments must have been conducted rigorously, with appropriate controls, replication, and sample sizes. The conclusions must be drawn appropriately based on the data presented. 

Reviewer #1: Yes

Reviewer #3: Partly

Reviewer #4: Yes

Reviewer #5: Yes

Reviewer #6: Yes

Reviewer #7: Yes

Reviewer #8: Partly

**********

3. Has the statistical analysis been performed appropriately and rigorously? 

Reviewer #1: Yes

Reviewer #3: Yes

Reviewer #4: Yes

Reviewer #5: Yes

Reviewer #6: Yes

Reviewer #7: Yes

Reviewer #8: No

**********

4. Have the authors made all data underlying the findings in their manuscript fully available?

The PLOS Data policy requires authors to make all data underlying the findings described in their manuscript fully available without restriction, with rare exception (please refer to the Data Availability Statement in the manuscript PDF file). The data should be provided as part of the manuscript or its supporting information, or deposited to a public repository. For example, in addition to summary statistics, the data points behind means, medians and variance measures should be available. If there are restrictions on publicly sharing data—e.g. participant privacy or use of data from a third party—those must be specified.

Reviewer #1: Yes

Reviewer #3: Yes

Reviewer #4: Yes

Reviewer #5: Yes

Reviewer #6: Yes

Reviewer #7: Yes

Reviewer #8: Yes

**********

5. Is the manuscript presented in an intelligible fashion and written in standard English?

PLOS ONE does not copyedit accepted manuscripts, so the language in submitted articles must be clear, correct, and unambiguous. Any typographical or grammatical errors should be corrected at revision, so please note any specific errors here.

Reviewer #1: Yes

Reviewer #3: No

Reviewer #4: Yes

Reviewer #5: Yes

Reviewer #6: Yes

Reviewer #7: Yes

Reviewer #8: Yes

**********

6. Review Comments to the Author

Please use the space provided to explain your answers to the questions above. You may also include additional comments for the author, including concerns about dual publication, research ethics, or publication ethics. (Please upload your review as an attachment if it exceeds 20,000 characters)

Reviewer #1: Congratulations on this paper, you have made a great effort to bring up this neglected topic on tomates in our country. We would do well to take note on this, thak you.

Reviewer #3: The author has English problems as many grammatical and spelling mistakes were detected. In addition, inconsistency in the font type and size and misordering of the references (please find a reviewed copy of the manuscript).

Reviewer #4: The text submitted for review meets all of the criteria set by the editors (presents the results of the original work, is well prepared from the methodical and graphic aspects, contains substantive justification for the problem posed, etc.).

However, I would suggest the authors to make two corrections:

1) in the introductory section, complete the date of the conducted study (the year 2019 appears in the main text - please provide this information also in the abstract)

2) enrich the passage about the harmfulness of pesticide residues in tomatoes with the results of the literature review

Reviewer #5: The author has presented a well written article (Consumer risk perception towards pesticide stained tomatoes in Uganda), detailed, and supported by solid analysis. However, reviewers must make some suggestions to improve this manuscript's quality, including:

1. The author needs to add novelty and importance to this study. In addition, the authors should mention the contribution of this study to theoretical and practical development.

2. The manuscript does not have a strong theoretical foundation. I recommend the author to add a theory section.

3. The author must include a research framework (in the form of figures) in the methods section.

4. The author must explain why the number of respondents in each district is different and disproportionate to the population in each region

5. The author needs to add reliability and validity tests

6. The author must adjust the structure of the results section according to the structure in the method (optimism, pessimism and trust)

7. The author states that there are 468 respondents, but the number of respondents in each discussion subsection is different. Please be consistent! The author has to calculate everything again and also make sure all percentages add up to 100%.

8. The author needs to add activities that have been conducted by the government and the community of Uganda to control the use of pesticides in the discussion section

9. The author needs to add limitations and further research in the conclusion section.

Finally, I hope these various suggestions can help you improve this manuscript quality and be published in the Plos One journal. Good luck!

Reviewer #6: (No Response)

Reviewer #7: Please see comments as well as brief letter to the editor attached in the word document, and the reviewed manuscript.

Reviewer #8: The topic is interesting an contributes to the issue of healthy food. However, the authors should address some weaknesses of the manuscript. My specific points are detailed below:

1. I think the weakest part of the article is the methodology justification. The mixed-method approach should be explained in more detail. As far I can see, this is an explanatory sequential design. That is, a quantitative stage followed by a qualitative one. The rationale for the latter stage should be justified in detail. For example, what quantitative results are considered as key aspects to be explored by qualitative assessment? Also, qualitative analysis is normally addressed by means of the grounded theory. It would be beneficial to learn if the authors adopted this formal technique or an alternative one. In relation to the quantitative stage, I suggest adding a table with the Likert-scale statements employed in the questionnaire and a brief justification for the selection of them.

2. The result section could be improved by using less subheadings and more narrative.

3. Regarding positive vs. negative attitudes towards pesticide stained tomatoes, I am confused. Why not to join them as attitudes only? This is because the authors are using a Likert-scale implying that it explicitly captures respondents who have positive attitudes (i.e. agree) and negative attitudes (disagree).

4. The discussion section is reasonable, although I suggest adding more implications for policymakers and farmers. This will help visualising more possible options to address the problem of pesticide.

5. Conclusions should contain, at the end, limitations of the research and possible extensions for future investigations.

**********

7. PLOS authors have the option to publish the peer review history of their article (what does this mean?). If published, this will include your full peer review and any attached files.

If you choose “no”, your identity will remain anonymous but your review may still be made public.

**Do you want your identity to be public for this peer review?** For information about this choice, including consent withdrawal, please see our Privacy Policy.

Reviewer #1: **Yes: **Douglas Barraza

Reviewer #3: No

Reviewer #4: No

Reviewer #5: **Yes: **AGUS DWI NUGROHO

Reviewer #6: **Yes: **Muhammad Asad ur Rehman Naseer

Reviewer #7: **Yes: **Elijah N Muange

Reviewer #8: No

**********

[NOTE: If reviewer comments were submitted as an attachment file, they will be attached to this email and accessible via the submission site. Please log into your account, locate the manuscript record, and check for the action link "View Attachments". If this link does not appear, there are no attachment files.]

While revising your submission, please upload your figure files to the Preflight Analysis and Conversion Engine (PACE) digital diagnostic tool, https://pacev2.apexcovantage.com/. PACE helps ensure that figures meet PLOS requirements. To use PACE, you must first register as a user. Registration is free. Then, login and navigate to the UPLOAD tab, where you will find detailed instructions on how to use the tool. If you encounter any issues or have any questions when using PACE, please email PLOS at figures@plos.org. Please note that Supporting Information files do not need this step.

Attachment Submitted filename: PONE-D-21-04826_R1.docx Click here for additional data file.

Attachment Submitted filename: PONE-D-21-04826_R1.pdf Click here for additional data file.

Attachment Submitted filename: Reviewer comment.docx Click here for additional data file.

Attachment Submitted filename: PLos One - Copy.docx Click here for additional data file.

Attachment Submitted filename: PONE-D-21-04826_R1_reviewer_EM.pdf Click here for additional data file.

Attachment Submitted filename: PONE-D-21-04826R1_reviewer-EM_Report.docx Click here for additional data file.

# Author comment: Author response to Decision Letter 1

**DOI:** 10.1371/journal.pone.0247740.r004

30 Mar 2023

Dear editorial manager,

I take this opportunity once again to so much thank the reviewers of this manuscript for the insights rendered and tireless corrections pointed out. These can never be taken for granted.

We have taken time responding to each of the comments raised by the different reviewers and happy to lay them to you for further guidance.

Please find below a list of comments and respective responses. Please make all your references to the “Revised manuscript with track changes” document for changes made.

Thank you!

Reviewer 1 comments and response

Title: Consumer risk perception towards pesticide-stained tomatoes in Uganda

Manuscript Number: PONE-D-21-04826R1 

The manuscript entitled Consumer risk perception towards pesticide-stained tomatoes in Uganda is a good contribution to the existing body of knowledge in the related discipline. The paper used a mixed methods cross-sectional dataset of 468 household consumers in four regions of Uganda. The paper concluded that Consumer risk perception on pesticide-stained tomatoes among Ugandan consumers ranked low with majority of the consumers buying tomatoes stained with pesticide residues due to lack of an alternative. I am satisfied with the paper to be published with the following minor changes.

• I found English and sentence structuring throughout the paper a bit week that is needed to be addressed. 

Response: the whole manuscript has been reviewed and English weakness addressed refer to revised manuscript with track changes .docx.

• Keywords may be changed 

Response: keywords have been added and others maintained unless suggestions for change are provided by the reviewers.

• In the methodology section the functional form of the model used may be explained and give the reference of the model adapted from.

Response: research model used has been inserted with reference and model theory explained. see methodology section.

• I think discussion section need more references/literature cited. 

Response: more references to the results have been inserted and discussed. See document with track changes page. References have been provided basing on what has been discussed unless the reviewer is specific on where references should be provided.

• All the results should be balanced with some references with prior studies and may be discussed in the discussion section.

Response: refer to response above.

Reviewer 2 comments and responses

The author has presented a well written article (Consumer risk perception towards pesticide stained tomatoes in Uganda), detailed, and supported by solid analysis. However, reviewers must make some suggestions to improve this manuscript's quality, including:

1. The author needs to add novelty and importance to this study. In addition, the authors should mention the contribution of this study to theoretical and practical development.

Response: a paragraph has been inserted stating on contributions made by this study to existing knowledge base of consumer risk perception and also results being used as a justification for the establishment of a nation pesticide residue monitoring program for Uganda. See last paragraph of introduction page 8.

2. The manuscript does not have a strong theoretical foundation. I recommend the author to add a theory section.

Response: theoretical section has been added to the manuscript see page9. At the beginning of the methodology section.

3. The author must include a research framework (in the form of figures) in the methods section.

Response: a research framework narrative section been added to the manuscript with a reference to figure 1 which has also been revised to include the knowledge question. See page 9 for the tracked document.

4. The author must explain why the number of respondents in each district is different and disproportionate to the population in each region.

Response: numbers being different was due to excessive sampling done in some sub counties. This has no effect on the results it instead increased accuracy since all the targets per sampling unit was met. Our sample size had a target of 423 and in the end 468 participants were interviewed, i.e more than 100% of the targeted.

5. The author needs to add reliability and validity tests.

Response: we couldn’t perform these tests as we felt that they are not a must.

6. The author must adjust the structure of the results section according to the structure in the method (optimism, pessimism and trust)

Response: results of the study have been presented in the same manner of optimism, pessimism and trust see page 13-15.

7. The author states that there are 468 respondents, but the number of respondents in each discussion subsection is different. Please be consistent! The author has to calculate everything again and also make sure all percentages add up to 100%.

Response: that only applies to question which were not supposed to be answered by the whole sample forexample for questions which followed a screening question as indicated in fig3. there has been consistency in the total number of respondents presented in each section.

8. The author needs to add activities that have been conducted by the government and the community of Uganda to control the use of pesticides in the discussion section.

Response: a paragraph has been stated on this see page..31

9. The author needs to add limitations and further research in the conclusion section.

Response: study limitation other research recommendations have been included see page 32

Finally, I hope these various suggestions can help you improve this manuscript quality and be published in the Plos One journal. Good luck!

Dear Editor,

Thank you for considering me to review the manuscript entitled “Consumer risk perception towards pesticide stained tomatoes in Uganda”, PONE-D-21-04826R1.

I have reviewed the manuscript and note that the authors have addressed most of the comments raised in earlier review. 

Nevertheless, I have a number of minor comments that I believe if addressed will add value to the article and clarify issues for readers. My comments are provided below and are also highlighted in the manuscript that I will attach. 

I thank you and look forward to working with you in future.

Kind regards

Elijah N Muange (PhD)

Reviewer

3rd October, 2022

Title: Could be edited to include attitude, thus “Consumer attitude and risk perception ...”

Abstract

1. It is indicated that “…European Union Maximum Residue Limits (used as a standard in Uganda)”. Response: These MRL used only during the vetting process for exports of foods from Uganda.

2. If this standard has been domesticated in Uganda, then authors could just say the pesticide residues are above the permitted MRLs in the country.

Response: comment has been adopted and rectified refer to abstract.

3. Total participants in FGD should also have been mentioned.

Response: total FGD participants have been mentioned in the abstract.

4. It is good to mention the regions where the study was conducted

Response: regions have been inserted in the manuscript text and felt not necessary to still mention them in the summary abstract.

5. The project should be introduced very briefly so that readers may relate the study to the project. For example, what was the goal or objectives/activities? 

Response: the project brief has been included in the manuscript but not in the abstract.

Introduction

Page5

Should be specified that it is “pesticide” regulations

Response: specification of the regulation for pesticide application has been inserted see page 8 

Page6

• Although the general problem has been articulated on a global scale, the case for Uganda is not very explicit. What are the literature gaps this study seeks to fill? What have previous studies done and what remains to be done? 

Response: literature gaps have been inserted with references. See introduction section

Methods

Page6

• Did the study target only households that participated in the project? How were sampling frames constructed?

Response: sampling procedure has been inserted refer to methodology, study design on page14 

• As mentioned above (abstract), this project needs to be elaborated so that readers can relate it to the study (aims, activities, targeting, etc) 

Response: this is not a must since the project did this study as a stand-alone for advocacy purposes on consumer protection. 

Study design

P7

• Which sub counties were involved?

Response: Sub counties have been inserted; under study population subsection

• Were these numbers proportional to district population or aim was to have equal numbers per district?

 Response: equal numbers per district was the aim, proportionate sampling was not employed.

• In the results section, the respondents are 17. No explanation is given on responses of the other 19 or why they are missing from the analysis.

• Response: in qualitative interviews, response saturation is followed, the 2 respondents didn’t have a different view of the question.

Results

P11

• It may also have been good to describe the sample involved in FGD. Who were they - age, education level, gender, etc.

Response: sorry! these was not captured.

• Level of income could be a good determinant of preference and purchase decisions. It would have been interesting to capture it. alternately, there was a variable on occupation.

Response: this was capture but later found to be biased due to sensitivity of the question and implications since most respondents’ fear taxing income, they would give wrong income values. Yes occupation was captured and been used in the analysis and discussion.

Rable1:

• Male and female: these total to 467 and not 468, one is missing

Response: missing male has been added.

P12

• Do authors use stain to mean residues contained in the tomato, or physical staining outside the tomato fruit? This should be clarified from the start and throughout the document. Earlier reviewer raised similar concerns. Residuals is also used elsewhere. It would be good to distinguish these terms and use them appropriately.

Response: Residues and stains are used interchangeably, stains are a type of physical residue. This has been defined in the document in the introduction section.

Qualitative findings

P18

• These should be 36 going by the information in the methods section

Response: yes although not all the respondents had to answer the question answering depended much on level of saturation, until no more new responses were captured.

P20

• The highlighted area, is it residue or stain?

Response: please refer to comment above on pesticide residue and stain.

Discussion

• The second paragraph is merely descriptive of the sample characteristics and may not be necessary in the discussion section. The section should go straight to the main results

Response: the paragraph gives the reader a description of the sample under study 

P23

• A study is mentioned, but the place it was carried out is not mentioned.

Response: study areas has been mentioned under study area and population under methodology section.

General: It could have been interesting to find out whether participating in the project had any effect on attitudes or risk perception.

Response: yes, though it was not the aim by then and therefore not captured, thank you!

Attachment Submitted filename: Response to Reviewers.docx Click here for additional data file.

# Peer review: Decision Letter 2

**DOI:** 10.1371/journal.pone.0247740.r005

**Authors:** Alejandro Vega-Muñoz

20 Apr 2023

PONE-D-21-04826R2Consumer risk perception towards pesticide-stained tomatoes in Uganda.PLOS ONE

Dear Dr. Sekabojja,

Thank you for submitting your manuscript to PLOS ONE. After careful consideration, we feel that it has merit but does not fully meet PLOS ONE’s publication criteria as it currently stands. Therefore, we invite you to submit a revised version of the manuscript that addresses the points raised during the review process. Please submit your revised manuscript by Jun 04 2023 11:59PM. If you will need more time than this to complete your revisions, please reply to this message or contact the journal office at plosone@plos.org. When you're ready to submit your revision, log on to https://www.editorialmanager.com/pone/ and select the 'Submissions Needing Revision' folder to locate your manuscript file.

Please include the following items when submitting your revised manuscript:

- A rebuttal letter that responds to each point raised by the academic editor and reviewer(s). You should upload this letter as a separate file labeled 'Response to Reviewers'.
- A marked-up copy of your manuscript that highlights changes made to the original version. You should upload this as a separate file labeled 'Revised Manuscript with Track Changes'.
- An unmarked version of your revised paper without tracked changes. You should upload this as a separate file labeled 'Manuscript'.

If you would like to make changes to your financial disclosure, please include your updated statement in your cover letter. Guidelines for resubmitting your figure files are available below the reviewer comments at the end of this letter.

If applicable, we recommend that you deposit your laboratory protocols in protocols.io to enhance the reproducibility of your results. Protocols.io assigns your protocol its own identifier (DOI) so that it can be cited independently in the future. For instructions see: https://journals.plos.org/plosone/s/submission-guidelines#loc-laboratory-protocols. Additionally, PLOS ONE offers an option for publishing peer-reviewed Lab Protocol articles, which describe protocols hosted on protocols.io. Read more information on sharing protocols at https://plos.org/protocols?utm_medium=editorial-email&utm_source=authorletters&utm_campaign=protocols.

We look forward to receiving your revised manuscript.

Kind regards,

Alejandro Vega-Muñoz, Ph.D.

Academic Editor

PLOS ONE

[Note: HTML markup is below. Please do not edit.]

Reviewers' comments:

Reviewer's Responses to Questions

**Comments to the Author**

1. If the authors have adequately addressed your comments raised in a previous round of review and you feel that this manuscript is now acceptable for publication, you may indicate that here to bypass the “Comments to the Author” section, enter your conflict of interest statement in the “Confidential to Editor” section, and submit your "Accept" recommendation.

Reviewer #4: All comments have been addressed

Reviewer #5: All comments have been addressed

Reviewer #7: All comments have been addressed

**********

2. Is the manuscript technically sound, and do the data support the conclusions?

The manuscript must describe a technically sound piece of scientific research with data that supports the conclusions. Experiments must have been conducted rigorously, with appropriate controls, replication, and sample sizes. The conclusions must be drawn appropriately based on the data presented. 

Reviewer #4: Yes

Reviewer #5: Partly

Reviewer #7: Yes

**********

3. Has the statistical analysis been performed appropriately and rigorously? 

Reviewer #4: Yes

Reviewer #5: I Don't Know

Reviewer #7: Yes

**********

4. Have the authors made all data underlying the findings in their manuscript fully available?

The PLOS Data policy requires authors to make all data underlying the findings described in their manuscript fully available without restriction, with rare exception (please refer to the Data Availability Statement in the manuscript PDF file). The data should be provided as part of the manuscript or its supporting information, or deposited to a public repository. For example, in addition to summary statistics, the data points behind means, medians and variance measures should be available. If there are restrictions on publicly sharing data—e.g. participant privacy or use of data from a third party—those must be specified.

Reviewer #4: Yes

Reviewer #5: Yes

Reviewer #7: Yes

**********

5. Is the manuscript presented in an intelligible fashion and written in standard English?

PLOS ONE does not copyedit accepted manuscripts, so the language in submitted articles must be clear, correct, and unambiguous. Any typographical or grammatical errors should be corrected at revision, so please note any specific errors here.

Reviewer #4: Yes

Reviewer #5: No

Reviewer #7: Yes

**********

6. Review Comments to the Author

Please use the space provided to explain your answers to the questions above. You may also include additional comments for the author, including concerns about dual publication, research ethics, or publication ethics. (Please upload your review as an attachment if it exceeds 20,000 characters)

Reviewer #4: The changes introduced by the Author/Authors significantly enriched the article. Of course, you can discover more weaknesses, but you just have to learn how to write scientific texts, preferably on your own mistakes. Therefore, I believe that the next editions of this Author/Authors will be much more technically, substantively and editorially correct.

Reviewer #5: Dear authors,

Thank you for your efforts to improve and improve the quality of this manuscript. However, I am disappointed with your response that does not meet my suggestions.

1. I asked you to write a novelty for this manuscript, but I could not find it in your edited manuscript

2. You say added a theoretical section on page 9 (at the beginning of the methodology section). I have read this manuscript carefully and searched for the location of the theory according to your explanation, but I did not find it in either the track changes or no track changes of this manuscript. This is what makes this manuscript undirected because the selection of variables is not based on theory and the authors are unable to explain the contribution of this manuscript to the development of theory.

3. I understand your sample very much. But you should be able to make arguments why the number of samples in each subregion is different. Is the population beneficiary of the program in each sub-region different so that the number of samples in 1 sub-region is higher than in other sub-regions?

4. You stated the reliability and validity tests were not mandatory. How do you ensure that your questionnaire is valid and reliable?

5. I cannot find any limitations to this study and recommendations for further studies

Reviewer #7: Dear Editor,

The authors have addressed the comments raised satisfactorily and the document is much improved.

Just a small thing - the statistical software used is Stata, authors could delete Corp since StataCorp is the company.

**********

7. PLOS authors have the option to publish the peer review history of their article (what does this mean?). If published, this will include your full peer review and any attached files.

If you choose “no”, your identity will remain anonymous but your review may still be made public.

**Do you want your identity to be public for this peer review?** For information about this choice, including consent withdrawal, please see our Privacy Policy.

Reviewer #4: No

Reviewer #5: No

Reviewer #7: **Yes: **Dr. Elijah Muange

**********

[NOTE: If reviewer comments were submitted as an attachment file, they will be attached to this email and accessible via the submission site. Please log into your account, locate the manuscript record, and check for the action link "View Attachments". If this link does not appear, there are no attachment files.]

While revising your submission, please upload your figure files to the Preflight Analysis and Conversion Engine (PACE) digital diagnostic tool, https://pacev2.apexcovantage.com/. PACE helps ensure that figures meet PLOS requirements. To use PACE, you must first register as a user. Registration is free. Then, login and navigate to the UPLOAD tab, where you will find detailed instructions on how to use the tool. If you encounter any issues or have any questions when using PACE, please email PLOS at figures@plos.org. Please note that Supporting Information files do not need this step.

# Author comment: Author response to Decision Letter 2

**DOI:** 10.1371/journal.pone.0247740.r006

15 Jul 2023

Dear editorial manager,

Thank you for taking up this role again in reviewing our article, it's unfortunate that some of the comments were not addressed. Please look at the corrections made this time round. I hope the correction will meet your expectations and that of the research fraternity.

We have taken time to respond to each of the comments raised by the different reviewers and are happy to lay them to you for further guidance.

Please find below a list of comments and respective responses for reviewers #5 and #7. Please make all your references to the “Revised manuscript with track changes” document for changes made.

Thank you!

Title: Consumer risk perception towards pesticide-stained Tomatoes in Uganda

Manuscript Number: PONE-D-21-04826R1 

RE: PLOS ONE Decision: Revision required [PONE-D-21-04826R2

Reviewer comments and response

Reviewer #5: Dear authors,

Thank you for your efforts to improve and improve the quality of this manuscript. However, I am disappointed with your response that does not meet my suggestions.

1. I asked you to write a novelty for this manuscript, but I could not find it in your edited manuscript

Response: a paragraph has been edited, stating contributions made by this study to the existing knowledge base of consumer risk perception and also results being used as a justification for the establishment of a national pesticide residue monitoring program for Uganda. See the last paragraph of the introduction page 7.

2. You say added a theoretical section on page 9 (at the beginning of the methodology section). I have read this manuscript carefully and searched for the location of the theory according to your explanation, but I did not find it in either the track changes or no track changes of this manuscript. This is what makes this manuscript undirected because the selection of variables is not based on theory and the authors are unable to explain the contribution of this manuscript to the development of theory.

Response: A theoretical section has been inserted (just after introduction), please refer to page 7 of the tracked version.

3. I understand your sample very much. But you should be able to make arguments why the number of samples in each subregion is different. Is the population beneficiary of the program in each sub-region different so that the number of samples in 1 sub-region is higher than in other sub-regions?

Response: The sample deviated to the fact that some sub-counties had more participants than others, this was due to the difference in the social economic status and population of urban, rural and peri-urban areas selected from each district.

4. You stated the reliability and validity tests were not mandatory. How do you ensure that your questionnaire is valid and reliable?

Response: sorry about the oversight, the questionnaire had been adopted from another study, and we did not see it necessary to do the validity tests, maybe next time. 

5. I cannot find any limitations to this study and recommendations for further studies

Response: A section on study limitations has been inserted on page 29 see tracked version of the document just before the conclusion section.

Reviewer #7: 

Dear Editor,

The authors have addressed the comments raised satisfactorily and the document is much improved.

Just a small thing - the statistical software used in Stata, authors could delete Corp since StataCorp is the company.

Response: This has been done as shown in the tracked version of the manuscript pages 3 and 11.

Thank you again!

Attachment Submitted filename: Response to Reviewers.docx..docx Click here for additional data file.

# Peer review: Decision Letter 3

**DOI:** 10.1371/journal.pone.0247740.r007

**Authors:** Alejandro Vega-Muñoz

3 Aug 2023

Consumer risk perception towards pesticide-stained tomatoes in Uganda.

PONE-D-21-04826R3

Dear Dr. Sekabojja,

We’re pleased to inform you that your manuscript has been judged scientifically suitable for publication and will be formally accepted for publication once it meets all outstanding technical requirements.

Within one week, you’ll receive an e-mail detailing the required amendments. When these have been addressed, you’ll receive a formal acceptance letter and your manuscript will be scheduled for publication.

An invoice for payment will follow shortly after the formal acceptance. To ensure an efficient process, please log into Editorial Manager at http://www.editorialmanager.com/pone/, click the 'Update My Information' link at the top of the page, and double check that your user information is up-to-date. If you have any billing related questions, please contact our Author Billing department directly at authorbilling@plos.org.

If your institution or institutions have a press office, please notify them about your upcoming paper to help maximize its impact. If they’ll be preparing press materials, please inform our press team as soon as possible -- no later than 48 hours after receiving the formal acceptance. Your manuscript will remain under strict press embargo until 2 pm Eastern Time on the date of publication. For more information, please contact onepress@plos.org.

Kind regards,

Alejandro Vega-Muñoz, Ph.D.

Academic Editor

PLOS ONE

Reviewers' comments:

Reviewer's Responses to Questions

**Comments to the Author**

1. If the authors have adequately addressed your comments raised in a previous round of review and you feel that this manuscript is now acceptable for publication, you may indicate that here to bypass the “Comments to the Author” section, enter your conflict of interest statement in the “Confidential to Editor” section, and submit your "Accept" recommendation.

Reviewer #4: All comments have been addressed

Reviewer #5: All comments have been addressed

Reviewer #7: All comments have been addressed

**********

2. Is the manuscript technically sound, and do the data support the conclusions?

The manuscript must describe a technically sound piece of scientific research with data that supports the conclusions. Experiments must have been conducted rigorously, with appropriate controls, replication, and sample sizes. The conclusions must be drawn appropriately based on the data presented. 

Reviewer #4: Yes

Reviewer #5: Yes

Reviewer #7: Yes

**********

3. Has the statistical analysis been performed appropriately and rigorously? 

Reviewer #4: Yes

Reviewer #5: Yes

Reviewer #7: Yes

**********

4. Have the authors made all data underlying the findings in their manuscript fully available?

The PLOS Data policy requires authors to make all data underlying the findings described in their manuscript fully available without restriction, with rare exception (please refer to the Data Availability Statement in the manuscript PDF file). The data should be provided as part of the manuscript or its supporting information, or deposited to a public repository. For example, in addition to summary statistics, the data points behind means, medians and variance measures should be available. If there are restrictions on publicly sharing data—e.g. participant privacy or use of data from a third party—those must be specified.

Reviewer #4: Yes

Reviewer #5: Yes

Reviewer #7: Yes

**********

5. Is the manuscript presented in an intelligible fashion and written in standard English?

PLOS ONE does not copyedit accepted manuscripts, so the language in submitted articles must be clear, correct, and unambiguous. Any typographical or grammatical errors should be corrected at revision, so please note any specific errors here.

Reviewer #4: Yes

Reviewer #5: No

Reviewer #7: Yes

**********

6. Review Comments to the Author

Please use the space provided to explain your answers to the questions above. You may also include additional comments for the author, including concerns about dual publication, research ethics, or publication ethics. (Please upload your review as an attachment if it exceeds 20,000 characters)

Reviewer #4: I make no further comments - further amendments will not change the content, structure and wording of the text. At the same time, the total remodeling of this text is not the intention of the reviewer

Reviewer #5: Dear authors

I am very satisfied with your revision and have approved this manuscript for publication.

Best regards

Reviewer #7: Thank you for addressing the comments provided. Just one thing, this statement in the last paragraph of your discussion is hanging:

"Although quality assurance measures were put in place and followed by research assistants to ensure that all

reported and recorded data from respondents was the truth and nothing but the truth. Like all studies...."

Consider revising it.

**********

7. PLOS authors have the option to publish the peer review history of their article (what does this mean?). If published, this will include your full peer review and any attached files.

If you choose “no”, your identity will remain anonymous but your review may still be made public.

**Do you want your identity to be public for this peer review?** For information about this choice, including consent withdrawal, please see our Privacy Policy.

Reviewer #4: No

Reviewer #5: **Yes: **Agus Dwi Nugroho

Reviewer #7: **Yes: **Elijah N. Muange

**********

# Editor report: Acceptance letter

**DOI:** 10.1371/journal.pone.0247740.r008

**Authors:** Alejandro Vega-Muñoz

17 Aug 2023

PONE-D-21-04826R3 

Consumer risk perception towards pesticide-stained tomatoes in Uganda. 

Dear Dr. Sekabojja:

I'm pleased to inform you that your manuscript has been deemed suitable for publication in PLOS ONE. Congratulations! Your manuscript is now with our production department. 

If your institution or institutions have a press office, please let them know about your upcoming paper now to help maximize its impact. If they'll be preparing press materials, please inform our press team within the next 48 hours. Your manuscript will remain under strict press embargo until 2 pm Eastern Time on the date of publication. For more information please contact onepress@plos.org.

If we can help with anything else, please email us at plosone@plos.org. 

Thank you for submitting your work to PLOS ONE and supporting open access. 

Kind regards, 

PLOS ONE Editorial Office Staff

on behalf of

Dr. Alejandro Vega-Muñoz 

Academic Editor

PLOS ONE
